# Comprehensive miRNA Expression Analysis in Peripheral Blood Can Diagnose Liver Disease

**DOI:** 10.1371/journal.pone.0048366

**Published:** 2012-10-31

**Authors:** Yoshiki Murakami, Hidenori Toyoda, Toshihito Tanahashi, Junko Tanaka, Takashi Kumada, Yusuke Yoshioka, Nobuyoshi Kosaka, Takahiro Ochiya, Y-h Taguchi

**Affiliations:** 1 Department of Hepatology, Graduate School of Medicine, Osaka City University, Osaka, Japan; 2 Department of Gastroenterology, Ogaki Municipal Hospital, Ogaki, Japan; 3 Department of Medical Pharmaceutics, Kobe Pharmaceutical University, Kobe, Japan; 4 Department of Epidemiology, Infectious Disease Control and Prevention, Hiroshima University Graduate School of Biomedical Sciences, Hiroshima, Japan; 5 Division of Molecular and Cellular Medicine, National Cancer Center Research Institute, Tokyo, Japan; 6 Department of Physics, Chuo University, Tokyo, Japan; MOE Key Laboratory of Environment and Health, School of Public Health, Tongji Medical College, Huazhong University of Science and Technology, China

## Abstract

**Background:**

miRNAs circulating in the blood in a cell-free form have been acknowledged for their potential as readily accessible disease markers. Presently, histological examination is the golden standard for diagnosing and grading liver disease, therefore non-invasive options are desirable. Here, we investigated if miRNA expression profile in exosome rich fractionated serum could be useful for determining the disease parameters in patients with chronic hepatitis C (CHC).

**Methodology:**

Exosome rich fractionated RNA was extracted from the serum of 64 CHC and 24 controls with normal liver (NL). Extracted RNA was subjected to miRNA profiling by microarray and real-time qPCR analysis. The miRNA expression profiles from 4 chronic hepatitis B (CHB) and 12 non alcoholic steatohepatitis (NASH) patients were also established. The resulting miRNA expression was compared to the stage or grade of CHC determined by blood examination and histological inspection.

**Principal Findings:**

miRNAs implicated in chronic liver disease and inflammation showed expression profiles that differed from those in NL and varied among the types and grades of liver diseases. Using the expression patterns of nine miRNAs, we classified CHC and NL with 96.59% accuracy. Additionally, we could link miRNA expression pattern with liver fibrosis stage and grade of liver inflammation in CHC. In particular, the miRNA expression pattern for early fibrotic stage differed greatly from that observed in high inflammation grades.

**Conclusions:**

We demonstrated that miRNA expression pattern in exosome rich fractionated serum shows a high potential as a biomarker for diagnosing the grade and stage of liver diseases.

## Introduction

MicroRNAs (miRNAs) are a gene family that is evolutionarily conserved and have important roles in the control of many biological processes, such as cellular development, differentiation, proliferation, apoptosis, and metabolism [1]. Aberrant expression of miRNAs in liver tissue has been implicated in the progression of liver fibrosis, and hepatocarcinogenesis [2], [3], [4]. Recently, two independent groups showed that miR-122 plays a critical role in the maintenance of liver homeostasis and anti-tumor formation [5], [6].

Exosome in one of the endoplasmic reticulum carries mRNAs and miRNAs [7]. Recently, it has become clear that exosome perform intercellular signaling through miRNA. miRNAs are released through a ceramide-dependent secretory machinery and are then transferred and become functional in the recipient cells [8]. In a prior study using human blood and cultured cells, several miRNAs were selectively packaged into microvesicle (MV) and actively secreted [9]. In another study, miRNAs originating from EBV was transported by exosome and then participated in the immune response of host cells [10]. In HCC cells as well, this type of exosome-mediated miRNA transfer is an important mechanism of intercellular communication [11].

It has also become clear that exosome can adjust to immune function, control infection or carry the virus itself. Exosomes of T, B and dendritic immune cells contain a repertoire of miRNAs that differ from that of their parent cells [12], [13]. Exosomes released from nasopharyngeal carcinoma cells harboring latent EBV were shown to contain LMP1, signal transduction molecules, and virus-encoded miRNAs [14]. Retroviruses evade adaptive immune responses by using nonviral or host exosome biogenesis pathways to form infectious particles and as a mode of infection [15].

Recent evidence has shown that the expression patterns of serum or plasma miRNAs are altered in several diseases, in particular heart disease, sepsis, malignancies, and autoimmune diseases (reviewed in [16]). Discoveries such as this is encouraging and has propelled further research leading to the hypothesis that circulating miRNAs are detectable in serum and plasma in a form sufficiently stable to serve as biomarkers [17], [18]. One such example is that tumour-associated miRNAs were found in the serum of diffuse large B-cell lymphoma patients [19]. In other examples, serum levels of miR-34a and miR-122 were associated with histological disease severity in patients with CHC or non-alcoholic fatty-liver disease (NAFLD) [20]. In fact, the serum level of miR-122 strongly correlates with serum ALT activity and with necro-inflammatory activity in patients with CHC and elevated ALT levels. However, there seems to be no significant correlation between fibrosis stage and functional capacity of the liver [21]. The expression levels of miR-122 and miR-194 correlated negatively with age in patients with CHB and HBV associated acute-on-chronic liver failure [22]. The expression level of miR-122 in serum was found to be closely related to non drug-induced acute liver injury [23]. Based on the above, it comes as no surprise that recently, the expression profile from extracellular miRNA is being used clinically to diagnose various diseases.

Here, in order to obtain data with high resolution that is reproducible, we extracted MVs from serum using exoquick and then performed a comprehensive microarray analysis. We attempted to diagnose HCV infection, and ascertain the degree of liver inflammation and fibrosis stage using exosome-rich fractioned miRNA. In short, we investigate if serum-derived miRNAs had the potential to serve as non-invasive bio-markers for various liver diseases.

## Results

### Reproducible Gene-analysis Using Microarray

**Figure 1:**
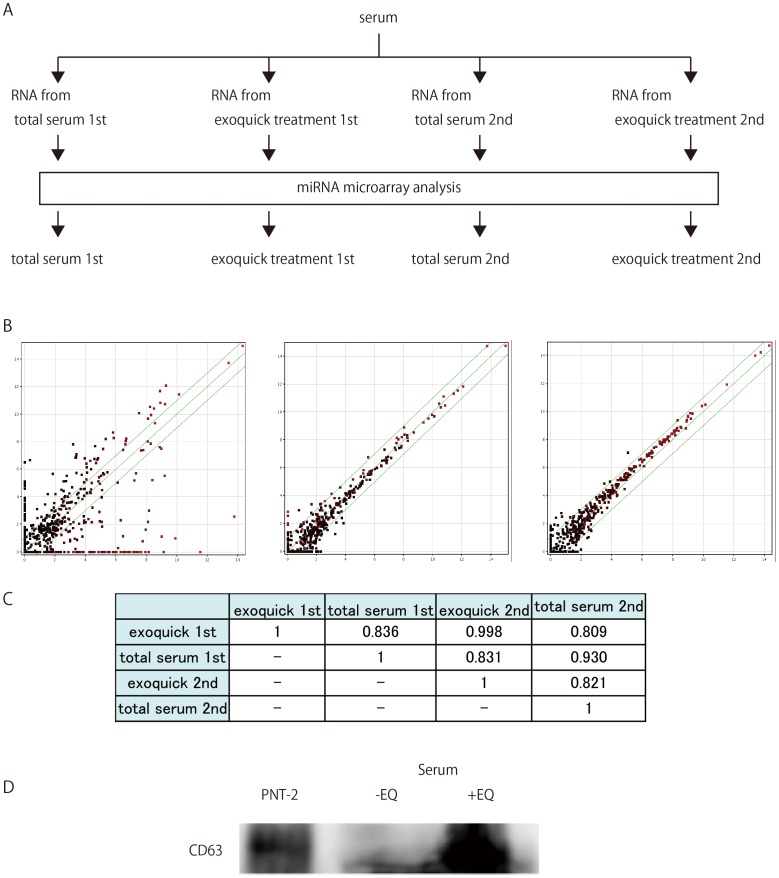
The method used to obtain reproducible data for microarray analysis conducted on serum-extracted samples. A. NL patients’ serum were sampled twice. In the first, RNA was extracted first from untreated serum, and then extracted again from serum treated with exoquick. In the second serum sample, RNA was also extracted from both untreated serum and serum treated with exoquick. Microarray analysis was conducted for RNA in a total of four samples. B. Reproducibility test of microarray data. Scatter plots comparing non- normalized signal intensities of miRNAs in two independent experiments from human total serum and exosome rich fraction. Red and black denotes high and low miRNA expressions respectively. Total serum extracted first, versus exosome rich fraction first (left), total serum extracted first versus second (middle), and exosome rich fraction extracted first versus second (right). C. Pearson’s pairwise correlations of signal intensities of miRNAs from human total serum and exosome rich fraction. D. Western blot was performed for untreated serum, serum extracted by exoquick and exosome fraction from PNT-2, using anti-CD63.

In microarray experiments, serum analysis is comparatively easy; however, the downside is that the accuracy and reproducibility of the results are usually not satisfactory. To circumvent this drawback, we devised a procedure that would give us higher accuracy and reproducibility. Serum samples from NL subjects were prepared and divided into two groups; for the first, RNA was extracted using exoquick treated serum, and in the second, RNA was extracted from total serum. Next, miRNA expression was analyzed using Agilent miRNA microarray. The above procedure was performed independently twice (**Fig. 1A**). We compared the miRNA expression pattern among the four microarray results (**Fig. 1B**) and found that miRNA expression analysis using exoquick was the more reliable and reproducible (**Fig. 1C**).

Exosome from normal human prostatic cell lines PNT-2, was yielded by the conventional ultra-centrifugation method [8]. We prepared serums with and without exoquick treatment and performed immunoblot analysis with anti-CD63 (**Fig. 1D**). Bands of the expected relative sizes were detected in serum treated with exoquick. We designated RNA extracted using exoquick treated serum as exosome-rich fractionated RNA.

### Unique Expression Pattern of miRNA in CHC

**Figure 2:**
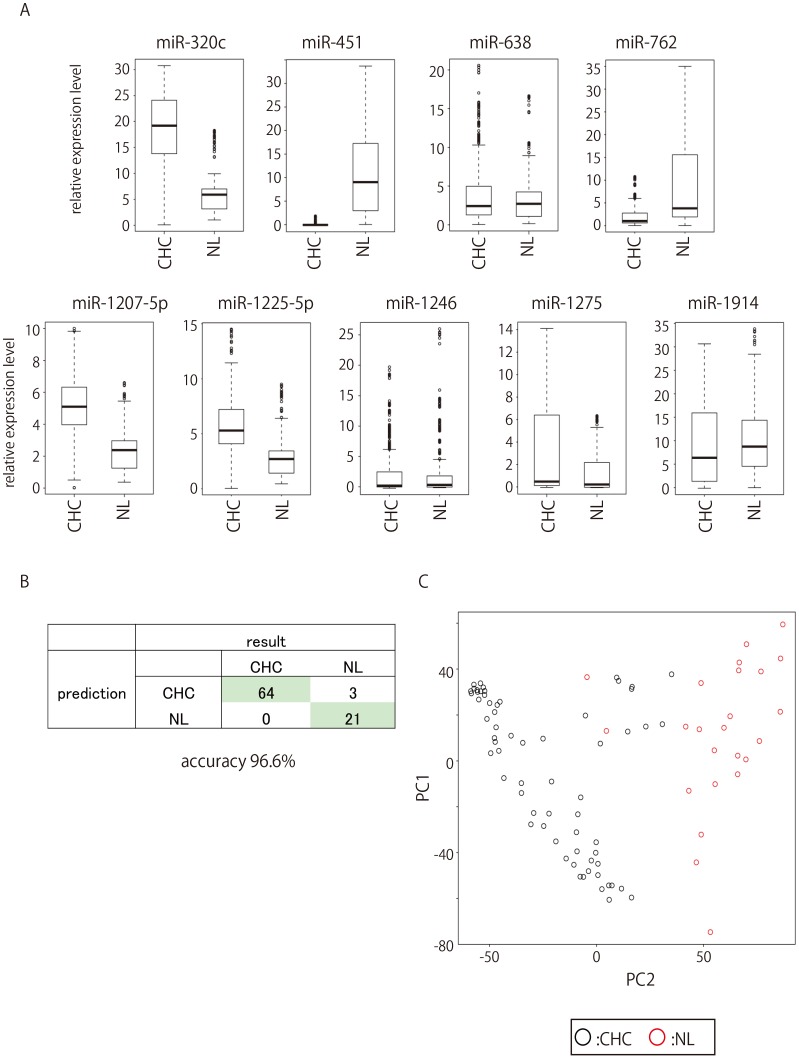
Expression patterns of miRNA used for discriminating between CHC and NL. A. Box plots of expression patterns of the nine miRNAs used for discriminating between CHC and NL. B. Classification of CHC and NL using LOOCV from miRNA expression profile. C. PCA in CHC and NL. The two dimensional embedding of CHC and NL by PCA. The first and second principal component scores computed (not selected for discrimination) of normalized miRNA expression were employed for this plot. Computation was done with ALL.

**Figure 3:**
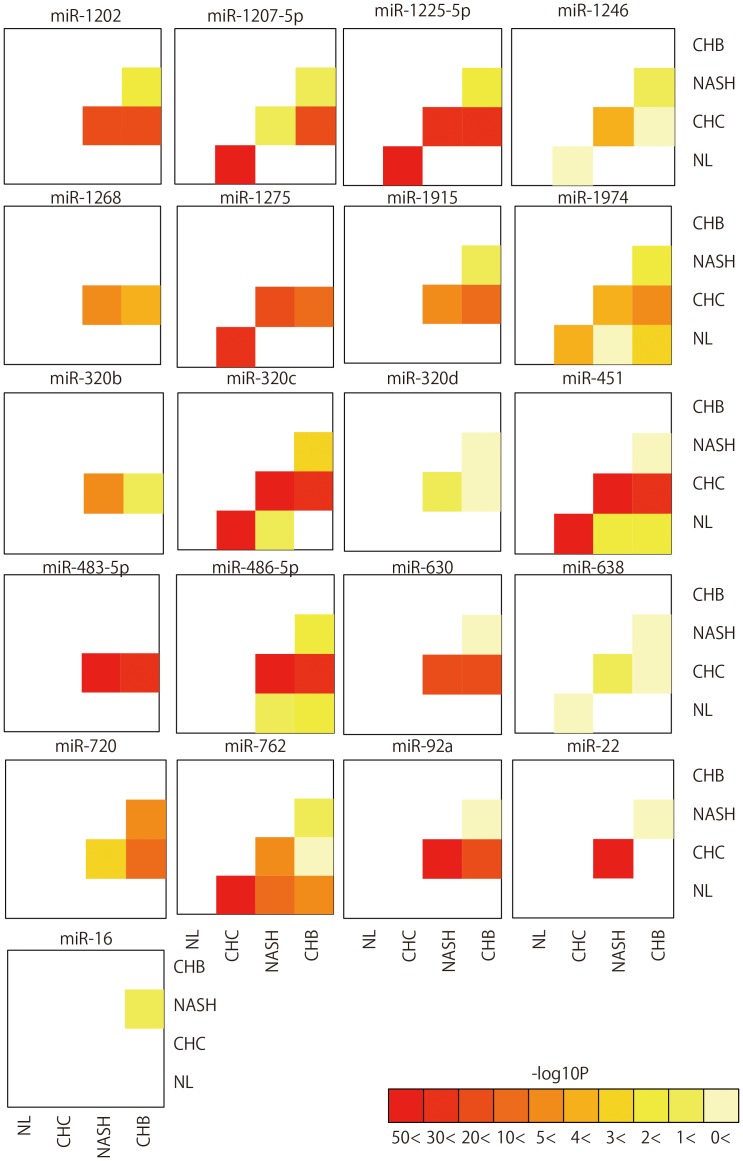
Pairwise heatmap of the miRNAs used for classifying two arbitrary groups. Pairwise heatmap showed the miRNAs and their p-value of two arbitrary groups.

**Figure 4:**
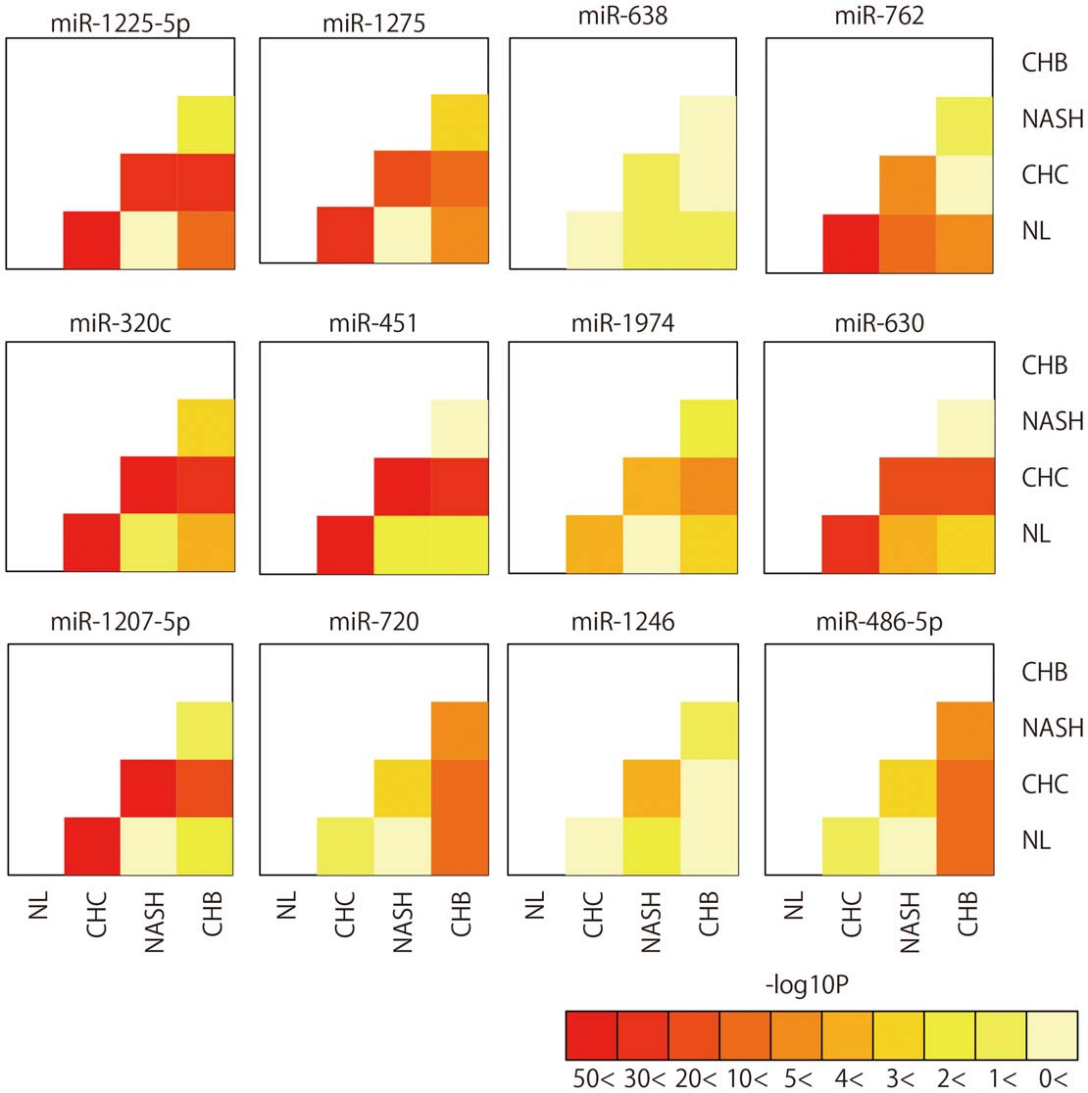
Pairwise heatmap of the miRNAs used for classifying among four groups.

We attempted to diagnose CHC using the miRNA expression pattern found in the peripheral blood samples from 64 CHC and 24 NL. The expression of nine miRNAs (miR-1225-5p, miR-1275, miR-638, miR-762, miR-320c, miR-451, miR-1974, miR-1207-5p, and miR-1246) allowed us to categorize patients as CHC or NL with 96.59% accuracy (**Fig. 2** **,** **3** Table 1 and Table S1). As shown in **Fig. 2C**, CHC and NL were well differentiated due to their distinct miRNA expression patterns. The expression pattern of 12 miRNAs led to the distinction of CHC, CHB, NASH, and NL with 87.50% accuracy (**Fig. 4** **, S1A,** and Table S1). The accuracy of determining whether samples were CHC or CHB, CHC or NASH, CHB or NASH was 98.35%, 97.37%, and 87.50%, respectively. The accuracy of judging whether samples were NL or CHB, NL or NASH, was 89.29% and 88.89%, respectively (**Fig. 3** **, S1B** and Table S1). Unlike CHC and NL, there were relatively fewer analyses done of CHB and NASH (due to a small sample size), therefore, we used *“in silico”* resampling to overcome any possible bias. With *“in silico”* we found that it was highly reproducible to determine with high accuracy whether samples were CHC, CHB, NASH, or NL, CHC or CHB, CHC or NASH, CHC or NL, CHB or NASH, CHB or NL, or finally NASH or NL (**Fig. S2 to S8** and Supporting Information).

In order to validate our above-mentioned classifications, we prepared a separate independent sample consisting of 31 CHC, 16 CHB, and 8 NASH. We established miRNA expression patterns using microarray for each of these chronic liver disease groups. We tried to discriminate among the classifications in the independent cohort using the semi-supervised learning method [24] based only on the labels in the original sample group and the selected miRNAs shown in Table S1. The accuracy of judging whether samples were CHB or CHC, CHC or NASH, CHB or NASH, was 74.47%, 87.18%, and 79.19%, respectively (**Fig. S9**, Table 1, and Supporting Information). During the process of obtaining these results, we noticed that different versions of the Feature Extraction (FE) Software provided slightly different results, however it was not possible to fully unify these versions of FE. This may explain the relatively lower performance of the independent group compared with the original samples that mostly used the same FE Software versions.

**Table 1:** Characteristics of subjects in this study of original samples and independent samples.

Original samples				
Characteristics	CHC	CHB	NASH	NL
Gender	F: 34/M: 30	F: 2/M: 2	F: 3/M: 9	F:11/M: 13
Age (years)	59.5±8.3	46.8±14.5	52.3±13.1	50.8±12.0
AST (IU/L)	50.1±29.8	83.3±53.7	46.2±16.0	N.D
ALT (IU/L)	57.6±40.6	167.8±170.3	74.5±34.9	N.D
WBC (x10^3^/mm^3^)	5.1±1.5	4.7±1.5	6.2±1.6	N.D
Platelet (x10^4^/mm^3^)	16.6±5.9	14.8±6.3	24.7±8.0	N.D
Total Bilirubin (mg/dl)	0.65±0.22	0.83±0.40	0.76±0.25	N.D
Weight (kg)	57.9±9.18	58.8±4.3	74.9±24.8	59.6±9.6
ALP (IU/L)	267.0±88.4	223.3±25.0	232.7±36.2	N.D
γGTP (IU/L)	46.9±42.3	77.3±82.2	58.4±20.9	N.D
Hemoglobin (g/dl)	13.8±1.2	14.5±0.59	14.7±1.6	N.D
Albumin (g/dl)	4.1±0.4	4.2±0.5	4.4±0.3	N.D
**Independent samples**				
**Characteristics**	**CHC**	**CHB**	**NASH**	
Gender	F: 18/M: 13	F: 10/M: 6	F: 6/M: 2	
Age (years)	59.5±8.3	46.8±14.5	54.8±12.7	
AST (IU/L)	50.1±29.8	83.3±53.7	80.9±50.0	
ALT (IU/L)	57.6±40.6	167.8±170.3	108.9±76.2	
WBC (x10^3^/mm^3^)	5.1±1.5	4.7±1.5	5.5±1.8	
Platelet (x10^4^/mm^3^)	16.6±5.9	14.8±6.3	19.3±7.6	
Total Bilirubin (mg/dl)	0.65±0.22	0.83±0.40	0.73±0.25	
Weight (kg)	57.9±9.18	58.8±4.3	66.4±9.9	
ALP (IU/L)	267.0±88.4	223.3±25.0	278.6±100.6	
γGTP (IU/L)	46.9±42.3	77.3±82.2	130.1±81.23	
Hemoglobin (g/dl)	13.8±1.2	14.5±0.59	13.6±1.4	
Albumin (g/dl)	4.1±0.4	4.2±0.5	3.8±0.3	

Abbreviations; CHC, chronic hepatitis C; CHB, chronic hepatitis B; NASH, non alcoholic steatohepatitis; NL, normal liver (healthy control); N.D, no data.

### miRNA Expression Correlates with the Grade of Liver Inflammation

**Figure 5:**
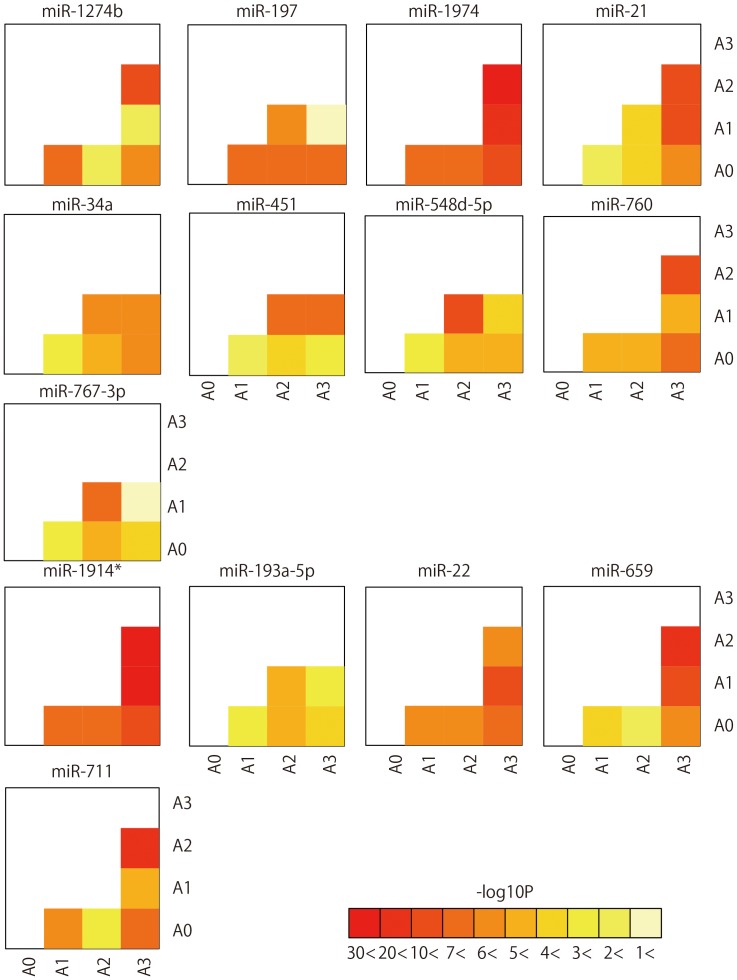
Significantly differentially expressed miRNAs according to liver inflammation grade. Pairwise heatmap showing the miRNAs and p-value of two arbitrary grades.

The grade of inflammation for CHC patients was ascertained by liver histological examination, and then samples were divided into four groups A0, A1, A2, and A3 based on their fibrosis stage. miRNA expression profiles were then established for CHC according to each of their inflammation grade. From the four groups (A0 to A3), a combination of six arbitrary pairs is possible. miRNAs which had significant differential expression in five or more of the six pairs were extracted (p<0.05). Five miRNAs (miR-1914*, miR-193a-5p, miR-22, miR-659, and miR-711) had expression levels that increased as the severity of liver inflammation progressed. On the other hand, the expression levels of nine miRNAs (miR-1274b, miR-197, miR-1974, miR-21, miR-34a, miR-451, miR-548d-5p, miR-760, and miR-767-3p) significantly decreased with the progression of liver inflammation (**Fig. 5** **, S10** and Table S2).

### The Grade of Liver Fibrosis Corresponded with the Expression Level of miRNAs

**Figure 6:**
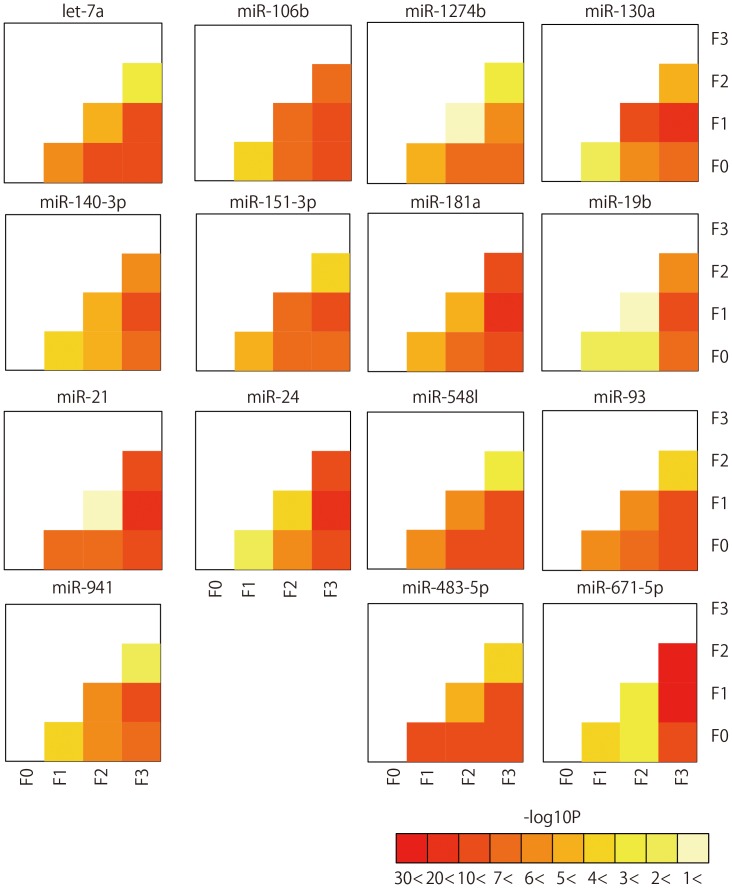
Significantly differentially expressed miRNA according to liver fibrotic stage. Pairwise heatmap showing the miRNAs and p-value of two arbitrary stages.

As previously noted, CHC samples were divided into F0, F1, F2, and F3 based on patients’ fibrotic stage. From these four fibrotic groups, a combination of six arbitrary pairs were possible. miRNAs that had significant differential expression in all six pairs were extracted (p<0.05). The expression levels of two miRNAs (miR-483-5p and miR-671-5p) significantly increased the higher the fibrotic stage and the expression level of 14 miRNAs (let-7a, miR-106b, miR-1274a, miR-130b, miR-140-3p, miR-151-3p, miR-181a, miR-19b, miR-21, miR-24, miR-375, miR-548l, miR-93, and miR-941) became progressively downregulated as liver fibrotic stage increased (**Fig. 6** **, S11** and Table S2).

### Classification of Liver Inflammation Grade and Fibrotic Stage Using miRNA Expression Pattern

**Figure 7:**
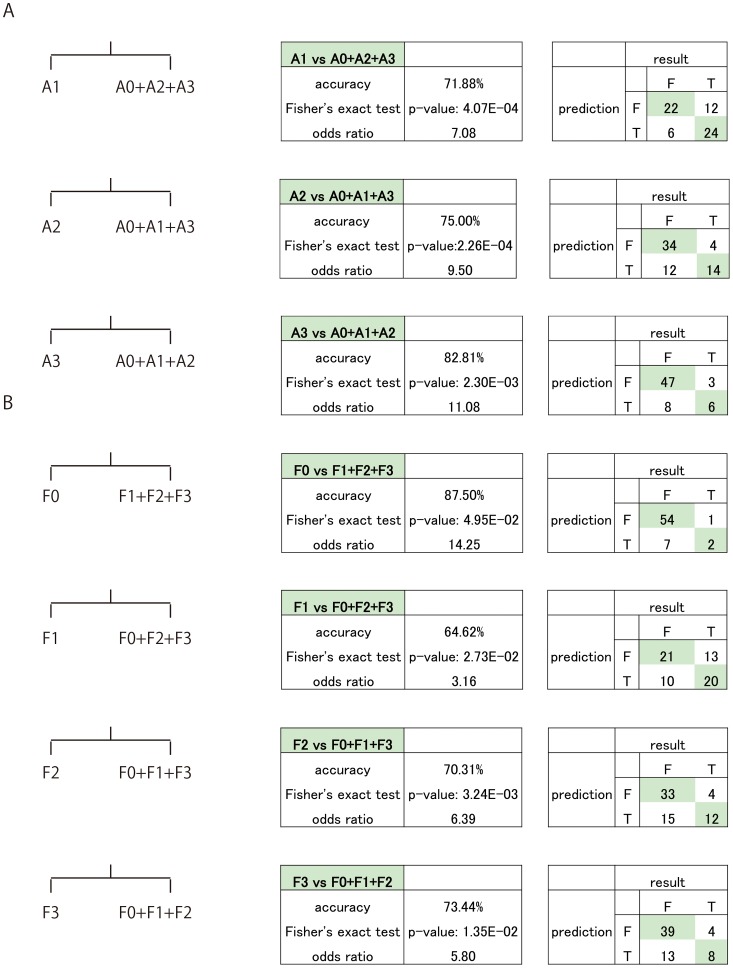
Determining liver inflammation grade and fibrotic stage using miRNA expression pattern in LOOCV analysis. A. In order to diagnose the grade of liver inflammation, A0 was identified first. Next A1, A2, and A3 were identified in a similar manner as A0. For each, the accuracy rate, P value, and the odds ratio are shown. B. For liver fibrosis stage, F0 was first diagnosed following which the other stages F1, F2, and F3 were diagnosed in a similar manner. For each group the accuracy rate, P value, and the odds ratio are shown.

We attempted to classify liver inflammation grade and fibrosis stage using miRNA expression pattern. Liver inflammation was diagnosed by Leave One Out Cross-Validation (LOOCV); the accuracy of determining A1 from other inflammation grade was 71.88% and its odds ratio was 7.08. The accuracy of determining A2 and A3 was 75.00% and 82.81%, and their odds ratios were 9.50 and 11.08, respectively. In our study, we were unable to accurately classify A0 because we were limited to only one sample for that grade (**Fig. 7A**). Diagnosis of liver fibrosis by LOOCV showed that determining F0 from the other fibrotic stages had an accuracy of 87.50% and an odds ratio of 14.25. The classification of F1, F2, and F3 had accuracy rates of 65.63%, 70.31%, and 73.44% and odds ratio of 3.16, 6.39 and 5.80, respectively (**Fig. 7B**).

### miRNA Expression Level Detected by Real-time qPCR Validated the Microarray Result

Four miRNAs (miR-1207-5p, miR-134, miR-1249, and miR-1183) with expression levels that differed among liver inflammation grades and liver fibrotic stages were chosen in order to confirm the microarray results using stem-loop based real-time qPCR. miRNAs that correlated with other clinical characteristics besides liver fibrosis and inflammation were listed using the Wilcoxon test. We performed two Wilcoxon tests and ranked miRNAs based on their p-value from smallest to largest and selected the miRNAs with the four smallest p-values that were common among the two Wilcoxon tests.

**Figure 8:**
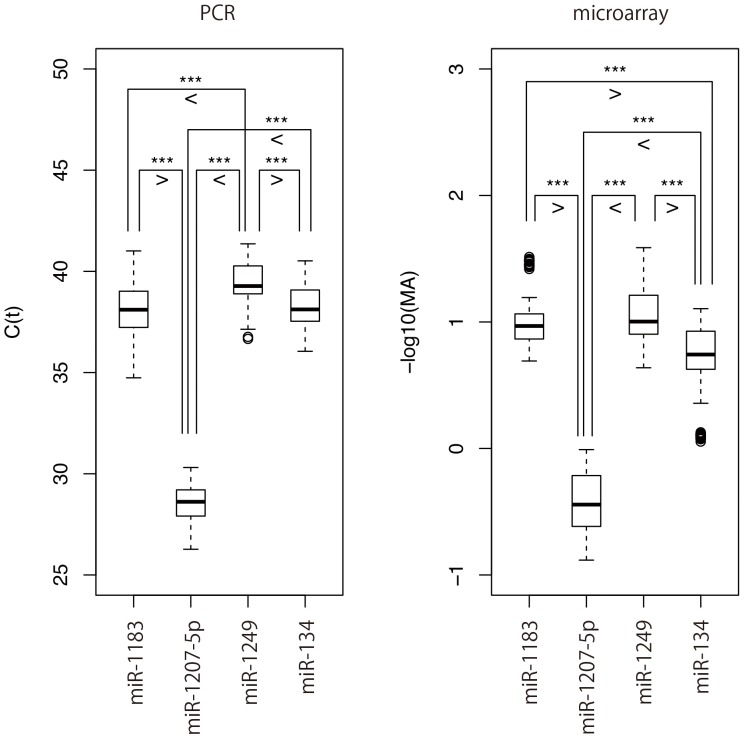
Real-time qPCR validation of microarray analysis. The microarray expression analysis result of four miRNAs was reproduced in real-time PCR analysis. The pairs with p<0.001 are marked by “***”.

The real-time qPCR result was consistent with the microarray analysis (**Fig. 8**). Here also, we applied *“in silico”* resampling to compensate for the small number of patients used in the real-time qPCR analysis. The results of the “*in silico*” resampling conferred with the results of the real-time qPCR (**Fig. S12**).

### miRNA Expression Pattern was Closely Related to Several Clinical Parameters in CHC

**Figure 9:**
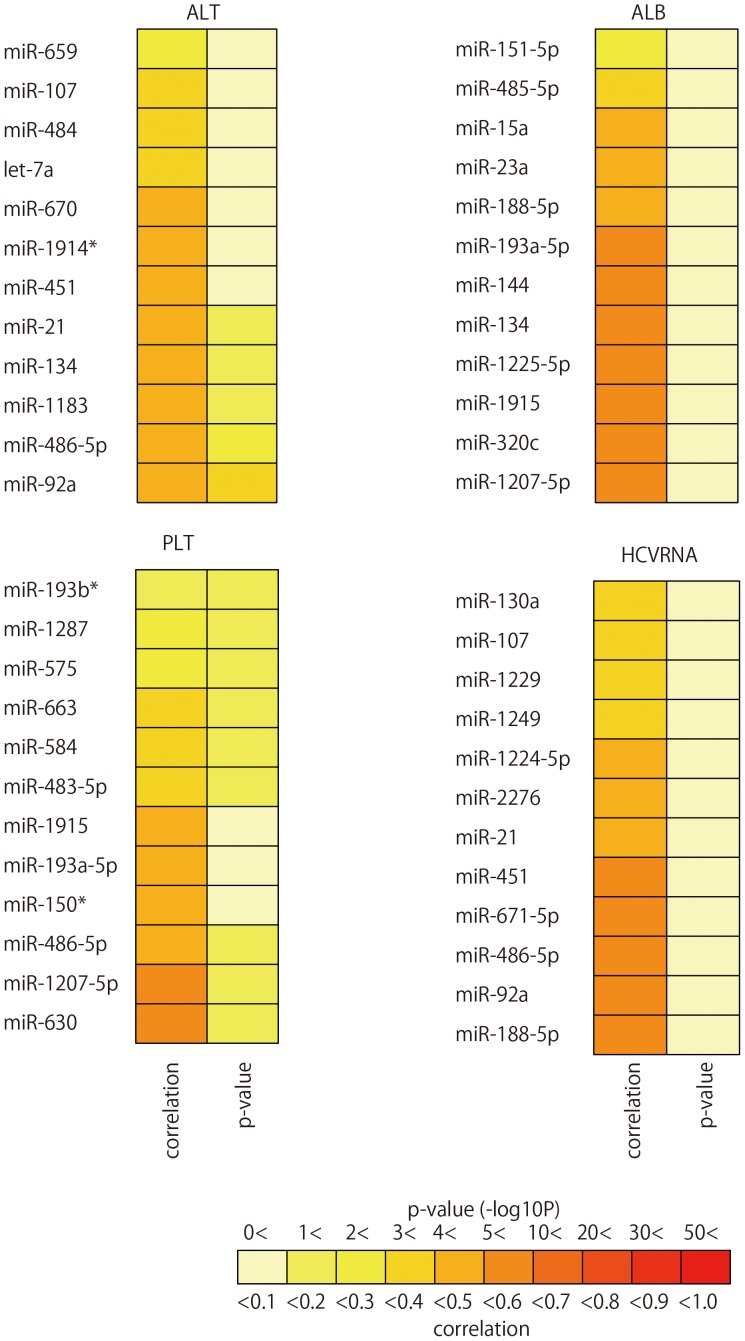
The list of miRNAs used to obtain the maximum correlation coefficient between miRNA expression level, and clinical characteristics. Pairwise heatmap showing miRNAs and their correlation coefficient and p-values.

Although we observed that miRNA expression correlated with ALT value, we were unable to identify miRNAs that displayed a strong correlation. 12 miRNAs were chosen sequentially from miRNAs with a high absolute correlation coefficient. One to 12 of these selected miRNAs were used to compare the canonical correlation coefficient of the above. When the expression patterns of six of the 12 miRNAs were compared with serum ALT value, the correlation coefficient and p-value were 0.44 and 4.91E−02, respectively. Similarly, when serum Albumin value was compared with the expression pattern of all 12 miRNAs, the correlation coefficient and p-value were 0.59 and 2.04E−02, respectively. Finally when the amount of serum HCVRNA was compared with the expression pattern of 12 miRNAs, the resulting correlation coefficient and p-value were 0.59 and 1.89E−02, respectively (**Fig. 9** **, S13** and Table S3).

### Expression Pattern of a Several miRNAs Correlated to Serum and Hepatic Tissue

In a previous report, we described the miRNA expression pattern found in liver tissues obtained from 105 CHC [2]. From this group, we analyzed the miRNA expression of hepatic tissue and serum in 60 samples. We observed that the expression pattern of three miRNAs (miR-134, miR-200b, miR-324-5p) in hepatic tissue negatively correlated with that in serum, and the expression pattern of miR-370 in hepatic tissue positively correlated with that in serum (p<0.05) (Table S4). However, there was no significant correlation between the expression pattern of miR-122 in the hepatic tissue and serum (**Fig. S14** and Table S4).

## Discussion

In this comprehensive miRNA analysis in various chronic liver diseases, we observed that aberrant expression of miRNAs was closely related to disease progression. Based on this, we believe that these miRNAs are potential readily accessible biomarkers, useful for diagnosing hepatic viral infection and for grading or staging liver diseases.

Many investigators have elected to use miRNA from serum instead of miRNA from exosome as the candidate for diagnosing diseases [18], [20], [22], [25], [26]. In our study, when exoquick was used, exosome could not be isolated therefore other MVs similar in size to exosome were also extracted. In other words, exoquick not only collected miRNAs contained in exosome, but also miRNA that were or were not combined with protein. Despite this, we found that exoquick delivered results that were superior to those obtained without exoquick. Therefore, although the process of analyzing miRNA from serum is simple, we chose to analyze miRNA from exosome rich fraction since it has a higher rate of reproducibility. Moreover, since exosome is closely related to intercellular signaling [14], [27], it is expected that data obtained by exosome analysis can clarify the mechanism of chronic infection and inflammation [28].

When we extended our analysis from miR-122 to all miRNAs, it became clear that the expression level of several miRNAs correlated with the progression of liver fibrosis. In fact, recent studies have stated that when the expression levels of adequate numbers of miRNAs is used to identify disease, diagnostic ability is significantly higher than using a single miRNA [29]. In this study, when liver fibrosis was diagnosed using miRNA expression, distinguishing between F0 and F1-3 was done with 87.50% accuracy. Since F0 cannot be distinguished from other stages of chronic liver disease using blood examination, we propose that using miRNA expression pattern may be useful for diagnosing chronic liver disease that is in the early stage.

Previous studies have shown that the level of miR-122 in blood plasma increased earlier than in ALT in the presence of toxic liver injury in rodents [30]. Serum levels of miR-122 in patients with CHC are frequently elevated compared with healthy individuals [21]. Bihrer et al. mentioned that variations in the concentration of miR-122 in serum or plasma tend to be more specific for liver diseases than ALT and AST. This is because miR-122 is almost exclusively expressed in the liver, whereas ALT and AST originate from skeletal muscles and other tissues, therefore their diagnostic value is low [31]. In our study, the expression level of miR-122 had a significant positive correlation with the grade of liver inflammation, serum albumin value, or serum HCVRNA value. However, miR-122 expression did not significantly correlate with liver fibrosis stage. Moreover, there was no correlation between the expression level of miR-122 in liver tissue, and that in serum in the same 60 samples (**Fig. S14**). The expression pattern of only four miRNAs out of total liver tissue miRNAs correlated with the expression patterns of miRNA found in the serum (Table S4). Most serum miRNA had expression patterns that differed from those observed in hepatic tissue samples. Moreover, we observed differences in miRNAs expression between various tissues [32]. These differences were observed even in tissues taken from the same subject; at present we are unclear as to the reason for this phenomenon.

In regards to the progression of liver fibrosis and the expression pattern of miR-21, previous studies concur with our result that miR-21 expression level significantly decreased in response to the progression of liver fibrosis [20]. Taken together, this suggests that any miRNAs that may have been emitted from liver tissue cannot be detected in serum after hepatic cell injury.

The expression pattern of many miRNAs in serum positively correlated with serum ALT, albumin, and HCVRNA levels in this study (**Fig. 9** **, S13** and Table S3). This result contradicts prior assumptions that no correlation exists between serum miR-122 and HCVRNA serum levels [21]. Three likely reasons for this difference in results are: 1) the detection method used (real-time qPCR versus microarray), 2) the difference in the subjects’ ages (the subjects in this study were older), and 3) the difference in the amount of miRNAs (multiple miRNAs vs. a single miRNA) used to identify the clinical parameters of the disease.

CHC and NL were classified with a high level of accuracy using the expression pattern of miRNA. In order to elucidate if the miRNA expression in CHC is common to other chronic liver diseases including CHB, we compared the miRNA expression pattern of CHC with those of NASH and CHB. The result of this analysis was that CHC could be clearly distinguished from both CHB and NASH. These results demonstrate that the varying forms of chronic liver disease have their own unique miRNA expression pattern. NASH is a histological diagnosis that rests on a combination of features and can only be confirmed by liver biopsy. Recently, NASH was diagnosed by first determining the existence of NAFLD from blood samples and then performing an ultrasound tomography. Finally, liver fibrosis stage was determined by Fibroscan (reviewed in [33]). However, when the results of these and other measures fail to yield a diagnosis then a pathology evaluation is necessary. Using “*in silico*” resampling to increase the reliability of our data, has led us to believe that NASH diagnosis may be possibly through blood examination.

We tested the reliability of our analysis in two ways and obtained reproducible results in both cases. First we enrolled an independent sample group, and second, we created virtual cohorts using in silico resampling to overcome our small sample size.

In this study we concluded that miRNA profiling is a promising alternative to diagnosing liver disease. This is based on our demonstration that the following evaluations could be performed using suitable miRNA expression profiles (1) determining the stage or grade of chronic liver disease, (2) ascertaining the clinical status of chronic liver diseases, and (3) distinguishing among various forms of chronic liver diseases. While these results suggest there is great potential and benefit of miRNA profiling, future studies in a larger population of CHC patients are warranted to fully elucidate the diagnostic potential of serum miRNA expression.

## Materials and Methods

### Patient Selection

A cohort of 64 CHC, 4 CHB, and 12 NASH patients who had undergone liver biopsy, as well as 24 healthy control subjects was enrolled. We also prepared independent samples consisting of 31 CHC, 12 CHB, and 8 NASH to validate our results. Patient characteristics are summarized in Table 1 and detailed clinical data is depicted in Table S5. The criteria for exclusion for CHC, CHB, and NASH were: co-infection with human immunodeficiency virus (HIV) types 1 and 2, decompensated liver disease, organ transplantation, immune suppression, autoimmune disorders, consumption of >20 g/day alcohol, and past history of intravenous drug abuse. Healthy controls were selected if they were not infected with HBV, HCV, nor HIV, had normal liver function tests, and had no history of liver disease.

All patients or their guardians provided written informed consent, and Ogaki Municipal Hospital and Kyoto University Graduate School and Faculty of Medicine’s Ethics Committee approved all aspects of this study in accordance with the Helsinki Declaration.

### Liver Histology and Blood Examination

A liver biopsy specimen was collected from each patient before anti-viral treatment. Histological grading and staging of CHC liver biopsy specimens were performed according to the Metavir classification system [34]. NASH was diagnosed histologically [35].

Serum HCV RNA was quantified before IFN treatment using Amplicor-HCV Monitor Assay (Roche Molecular Diagnostics Co., Tokyo, Japan), while serum HBV DNA was quantified before treatment using Amplicor HBV Monitor Assay (Roche). Pretreatment blood tests were conducted to determine each patient’s level of aspartate aminotransferase (AST), alanine aminotransferase (ALT), total bilirubin, alkaline phosphatase, gamma-glutamyl transpeptidase, white blood cell (WBC), platelets, and hemoglobin.

### Blood Sampling

Peripheral blood was collected from all subjects directly into serum tubes before anti-viral treatment. The tubes were centrifuged at 1,500 *g* for 10 min at 4°C, sera were aliquoted and additionally centrifuged at 2,000 *g* to completely remove any remaining cells. Sera were stored at −80°C until use.

### RNA Preparation

Total RNA from 200 ul of serum was prepared using miRNeasy mini kit (Qiagen, Hilden Germany) according to the manufacturer’s instruction. Exosome rich fractionated RNA was prepared using Exoquick (System Biosciences, CA, USA). Briefly, 900 ul of serum was mixed with 250 ul of Exoquick and incubated for 12 hr at 4°C. The tubes were centrifuged at 1500 g for 30 min at room temperature and then supernatant was discarded. The pellet was dissolved with 200 ul of PBS with vigorous vortex. RNA was extracted using miRNeasy mini kit (Qiagen).

### Immunoblot Analysis and Exosome Preparation

The procedure for exosome preparation has been previously described [8]. SDS-PAGE gels, SuperSep Ace 5–20% (194–15021) (Wako, Osaka, Japan), were calibrated with Precision Plus Protein Standards (161–0375) (Bio-Rad), and anti-CD63 (1∶200) was used as primary antibodies. The dilution ratio of each antibody is indicated in parentheses. Two secondary antibodies (peroxidase-labeled anti-mouse and anti-rabbit antibodies) were used at a dilution of 1∶5000. Bound antibodies were visualized by chemiluminescence using the ImmunoStar LD (Wako) and luminescent images were analyzed by a LuminoImager (LAS-3000; Fuji Film, Inc.). Only gels for CD63 (BD, NJ, USA) detection were run under non-reducing conditions. To exclude the albumin and IgG in serum, Albumin & IgG Depletion SpinTrap kit was used (GE health care, WI, USA). After aliquots isolation, exosome-contained fraction was isolated by Exoquick according to standard instructions.

### miRNA Microarray

To detect serum miRNA, 60 ng of RNA was labeled and hybridized using the Human microRNA Microarray Kit (Rel 14.0) (Agilent Technologies, CA, USA) according to the manufacturer’s protocol (protocol for use with Agilent microRNA microarrays Version 1.0). Hybridization signals were detected with a DNA microarray scanner G2505B (Agilent Technologies) and the scanned images were analyzed using Agilent feature extraction software (v9.5.3.1). We used raw data (gProcessedSignal) and normalized each expression so as to have zero mean and unit sample variance. The data presented in this manuscript have been deposited in NCBI’s Gene Expression Omnibus and are accessible through GEO Series access number GSE33857: http://www.ncbi.nlm.nih.gov/geo/query/acc.cgi?acc=GSE33857.

### Real-time qPCR for Human miRNA

To detect miRNA expression level by real-time qPCR, TaqMan® microRNA assay (Applied Biosystems) was used to quantify the relative expression levels of miR-1207-5p (assay ID. 241060), miR-134 (assay ID. 000459), miR-1183 (assay ID. 002841), and miR-1249 (assay ID. 002868). The expression level of miR-16 (assay ID. 000391) was also measured and used as an internal control. cDNA was synthesized using the Taqman miRNA RT Kit (Applied Biosystems). RNA (2 ng/ml) in 5 ml of nuclease free water was added to 3 ml of 5 × RT primer, 10×1.5 µl of reverse transcriptase buffer, 0.15 µl of 100 mM dNTP, 0.19 µl of RNase inhibitor, 4.16 µl of nuclease free water, and 50 U of reverse transcriptase in a total volume of 15 µl. The reaction was performed for 30 min at 16°C, 30 min at 42°C, and 5 min at 85°C. All reactions were run in triplicate. Chromo 4 detector (Bio-rad) was used to detect miRNA expression. To allow for the validation of microarray results with C(t) obtained by qPCR, raw gene expressions were transformed into logarithmic values. P-values were computed via one-sided t test. No averages over probes were taken for the microarray. The above procedures were also done with various packages/functions implemented in R (http://www.r-project.org/).

### Statistical Analysis

For symptoms having discrete values, grade pairs were compared with Wilcoxon rank sum test (one-sided); otherwise, P-values were computed from correlation coefficients. In both cases, false discovery rate (FDR) of less then 0.05 computed from the P-value was regarded as significant. Benjamini and Hochberg criterion was used for FDR estimation. All p-values shown are significant even though they are raw numbers. No average over probes was taken before correlation analyses.

### The Canonical Correlation Coefficients for miRNA Expression and Clinical Parameters

The canonical correlation coefficients were computed for ALT-miRNA, albumin-miRNA, and HCVRNA-miRNA correlations, using up to 12 miRNA with larger correlation coefficients (see Supporting Information).

### Classification Analyses for Liver Fibrosis/inflammation

P-values were computed via one-sided t test using the raw expression values of each miRNA from the samples of CHC and healthy controls. The logarithm of obtained P-values was then transformed into principal components scores via principal components analysis. Following this, grades were discriminated by linear discriminant analysis of CHC ages and the optimal number of principal components.

### Selection of miRNAs Required to Diagnose Several Liver Diseases

For specific pairs consisting of one liver disease and a healthy control, their normalized miRNAs expression was transformed into principal components scores via principal components analysis. miRNAs having the larger first and second principal component scores were selected. Following this, the principal component scores of each sample was computed based solely on the selected miRNA expressions. Liver diseases were classified using the optimal number of these principal component scores.

In order to compensate for the relative small number of NASH and CHB patients, we performed “*in silico*” patients resampling analysis of the microarray data (see Supporting Information). All the above procedures were done with various packages/functions implemented in R.

### “*In silico*” Resampling

*“In silico”* resampling is a tool often used to overcome the limitation of a small sample size. Using this technique, we combined the clinical traits of existing patients and created various virtual samples. Using these virtual cohorts, we were then able to increase the sample size (see Supporting Information).

In order to validate the *“in silico”* resampling results, we prepared another sample set and once again performed *“in silico”* resampling using the microarray data from 99 CHC liver tissue samples [36]. The results proved that “*in silico*” resampling can accurately reproduce an entire population using only a small number of existing samples (see Supporting Information).

### Reproducibility Test of Microarray Data

Data were analyzed using the GeneSpring GX10.0.2 (Agilent). Quality control (QC) was applied according to the manufacturer’s instructions, and all data were approved by GeneSpring. Following Agilent recommendations, no inter-array normalization was applied because the similarity in miRNA expression among sample arrays was unknown [37]. Scatter plots and Pearson’s pairwise correlations were performed with GeneSpring.

## Supporting Information

Figure S1 Expression patterns of miRNAs used for discriminating among CHC, NL, CHB, and NASH. Classifying CHC, NL, CHB, and NASH using LOOCV. Distinguishing between two arbitrary groups using LOOCV.
(TIF)

Figure S2 Expression patterns of miRNAs used to discriminate among CHC, CHB, NASH, and NL “in silico” resampling for disease discriminant studies reflected by BMI. A. Box plots of expression pattern of the miRNAs used to discriminate among CHC, CHB, NASH, and NL. B. Discriminating among four groups using LOOCV. Accuracy is 95.25%. C. Two dimensional embedding of CHC, CHB, NASH, and NL by the first and second principle component scores computed with 12 selected miRNAs
(TIF)

Figure S3 The same as Fig.3 for CHC and CHB. A. Box plot of 19 miRNAs used for the discrimination. B. Classification between CHC and CHB. Accuracy is 100%. C. The two dimensional embedding of CHB and CHC by the first and second principal component scores computed with19 selected miRNAs.
(TIF)

Figure S4 The same as Fig.S3 for CHC and NASH. A. Box plots of 20 miRNAs used for the discrimination. B. Classification between CHC and NASH. Accuracy is 100%. C. Two dimensional embedding of CHC and NASH by the first and second principal component scores computed with 19 selected miRNAs
(TIF)

Figure S5 The same as Fig.S3 for CHC and NL. A. Box plots of 9 miRNAs used for the discrimination. B. Classification between CHC and NL. Accuracy is 100%. C. Two dimensional embedding of CHC and NL by the first and second principal component scores computed with 9 selected miRNAs
(TIF)

Figure S6 The same as Fig.S3 for CHB and NL. A. Box plots of 4 miRNAs used for the discrimination. B. Classification between CHB and NL. Accuracy is 93.5%. C. Two dimensional embedding of CHB and NL by the first and second principal component scores computed with 4 selected miRNAs
(TIF)

Figure S7 The same as Fig.S3 for NASH and NL. A. Box plots of 5 miRNAs used for the discrimination. B. Distinguishing between NASH and NL with 84.0% accuracy. C. Two dimensional embedding of NASH and NL by the first and second principal component scores computed with 5 selected miRNAs
(TIF)

Figure S8 The same as Fig.S3 for CHB and NASH pair. A. Box plots of 17 miRNAs used for the discrimination. B. Distinguishing between CHB and NASH with 80.0% accuracy. C. Two dimensional embedding of CHB and NASH by the first and second principal component scores computed with 17 selected miRNAs
(TIF)

Figure S9 Classification of the independent sample using semi-supervised learning based on the labels in the original cohort. A. Classifying CHB and CHC. Accuracy is 74.47%. B. Classifying CHC and NASH. Accuracy is 87.18%. C. Classifying CHB and NASH. Accuracy is 79.19%.
(TIF)

Figure S10 miRNA expression pattern that correlated with the changes in clinical background. miRNAs that were differentially expressed according to the grade of liver inflammation
(TIF)

Figure S11 miRNA expression pattern that correlated with the changes in clinical background. miRNAs that were differentially expressed according to liver fibrosis stage
(TIF)

Figure S12 Real-time qPCR validation of microarray analysis “in silico” resampling for disease discrimination studies reflected by BMI. The result of microarray expression analysis of four miRNAs was reproduced using real-time PCR analysis. Pairs with p<0.001 are marked by “***”.
(TIF)

Figure S13 The relationship between the expression levels of several miRNAs and serum ALT, albumin, HCVRNA, respectively. Horizontal axis shows the number of miRNAs used in the analysis. Vertical axis shows the correlation index and p-values.
(TIF)

Figure S14 Summary of the relationship between the expression level of miR-122 and several clinical features. A. Expression level of miR-122 positively correlated with an increase in liver inflammatory grade. Asterisk denotes significant differences of p<0.05. B. Expression level of miR-122 positively correlated with the serum level of albumin. C. Expression level of miR-122 positively correlated with the amount of serum HCVRNA. D. Expression level of miR-122 in exosome rich fraction did not significantly correlate with that in liver tissues.
(TIF)

Table S1 The list of miRNAs used for classifying arbitrary 2 groups and 4 groups, and their p-values.
(DOCX)

Table S2 Significantly differentially expressed miRNAs according liver inflammation grade and liver fibrotic stage.
(DOCX)

Table S3 The list of miRNAs used to obtain the maximum correlation coefficient between expression level of miRNAs, and clinical characteristics.
(DOCX)

Table S4 List of miRNAs with expression that corresponded in liver tissue and serum.
(DOCX)

Table S5 Clinical background of original samples and independent samples in detail.
(DOCX)

Table S6 Accuracy of LDA for “in silico” resampling.
(DOCX)

Supplemental Information (DOCX)
