# Supplementary figures and images for: Comprehensive miRNA Expression Analysis in Peripheral Blood Can Diagnose Liver Disease
Source: PLoS One. 2012 Oct 31;7(10):e48366. doi: 10.1371/journal.pone.0048366 (PMC3485241; doi:10.1371/journal.pone.0048366)

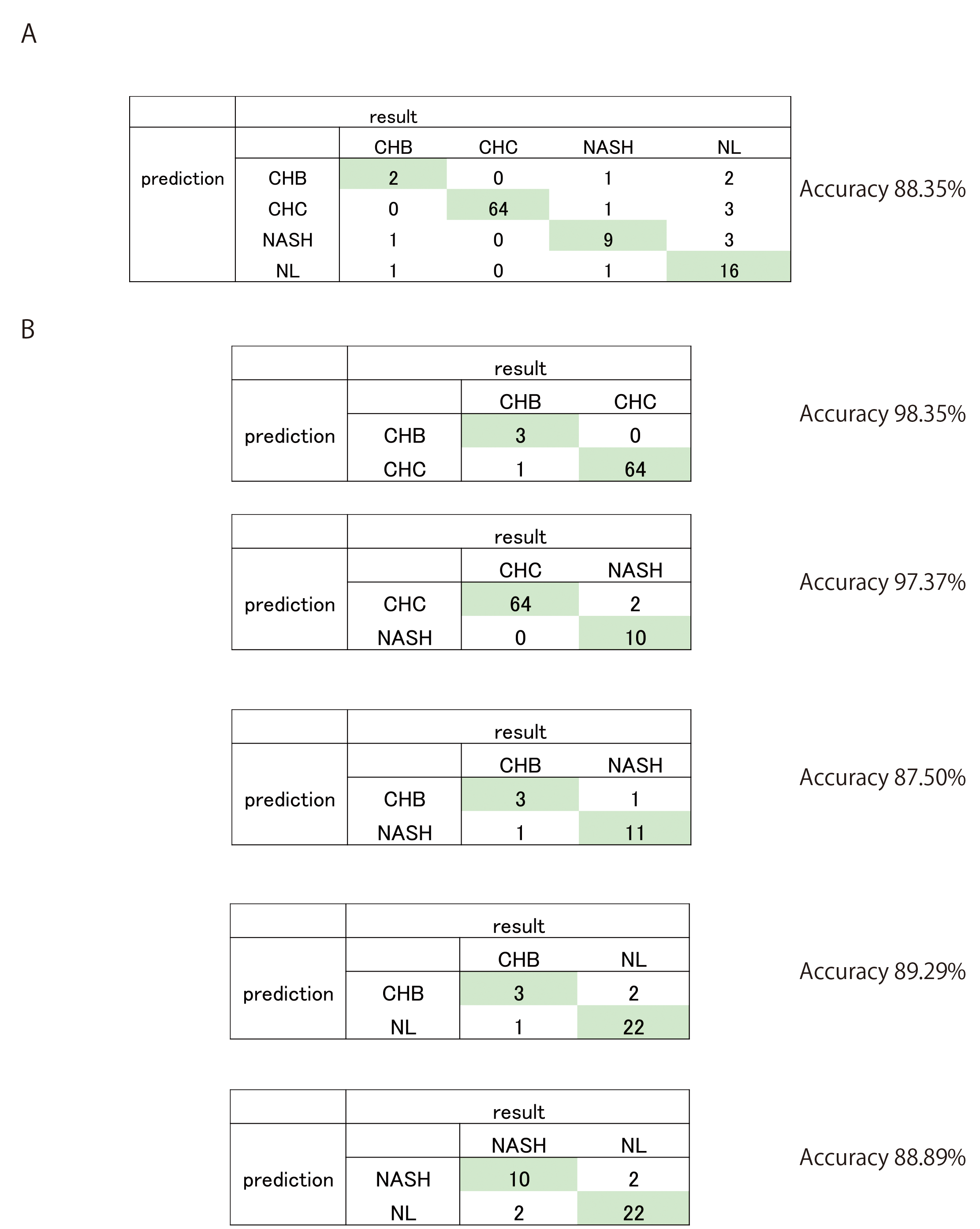

Supplement: Figure S1 — Expression patterns of miRNAs used for discriminating among CHC, NL, CHB, and NASH. Classifying CHC, NL, CHB, and NASH using LOOCV. Distinguishing between two arbitrary groups using LOOCV. (TIF) [file pone.0048366.s001.tif]

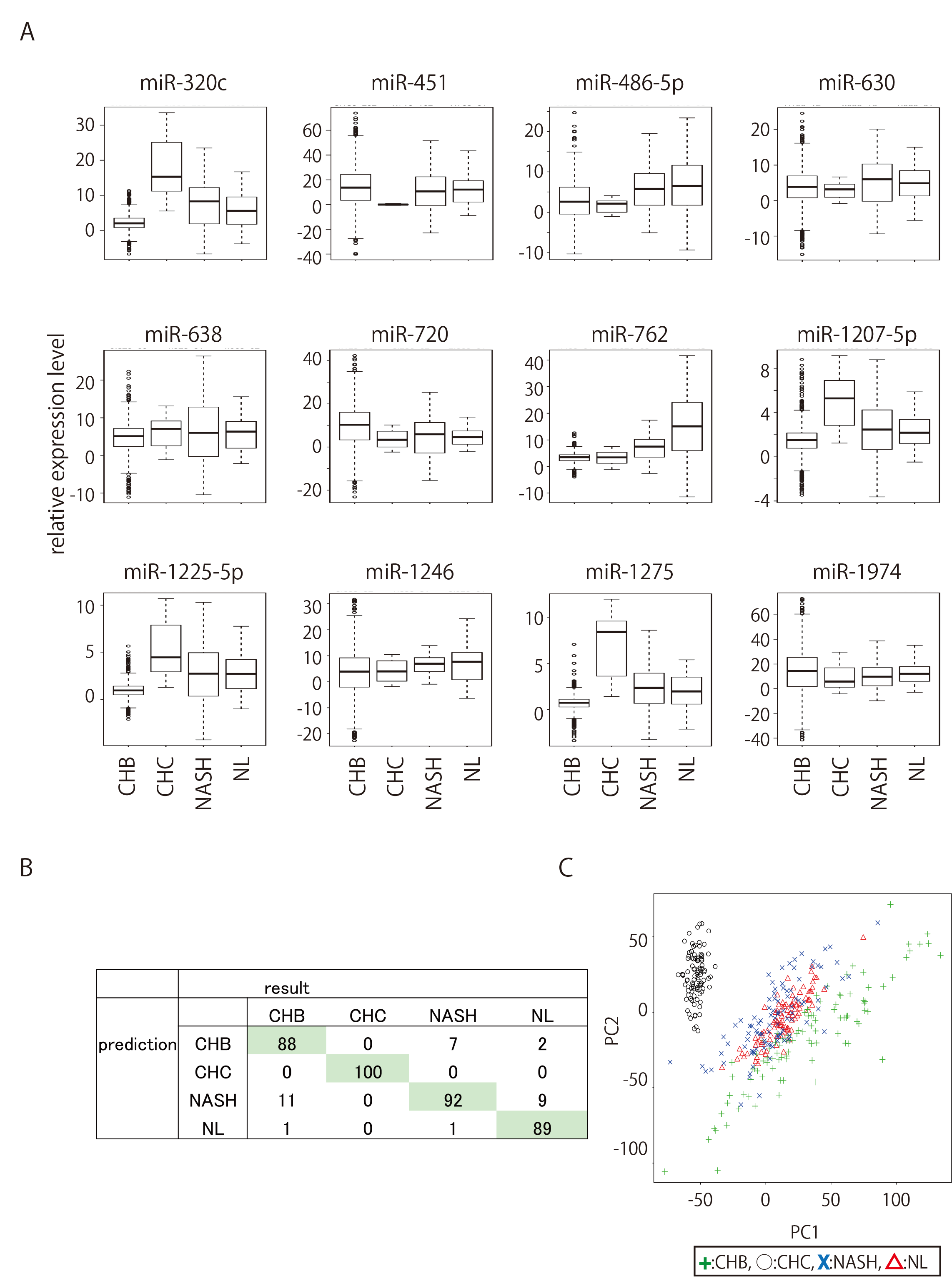

Supplement: Figure S2 — Expression patterns of miRNAs used to discriminate among CHC, CHB, NASH, and NL “in silico” resampling for disease discriminant studies reflected by BMI. A. Box plots of expression pattern of the miRNAs used to discriminate among CHC, CHB, NASH, and NL. B. Discriminating among four groups using LOOCV. Accuracy is 95.25%. C. Two dimensional embedding of CHC, CHB, NASH, and NL by the first and second principle component scores computed with 12 selected miRNAs (TIF) [file pone.0048366.s002.tif]

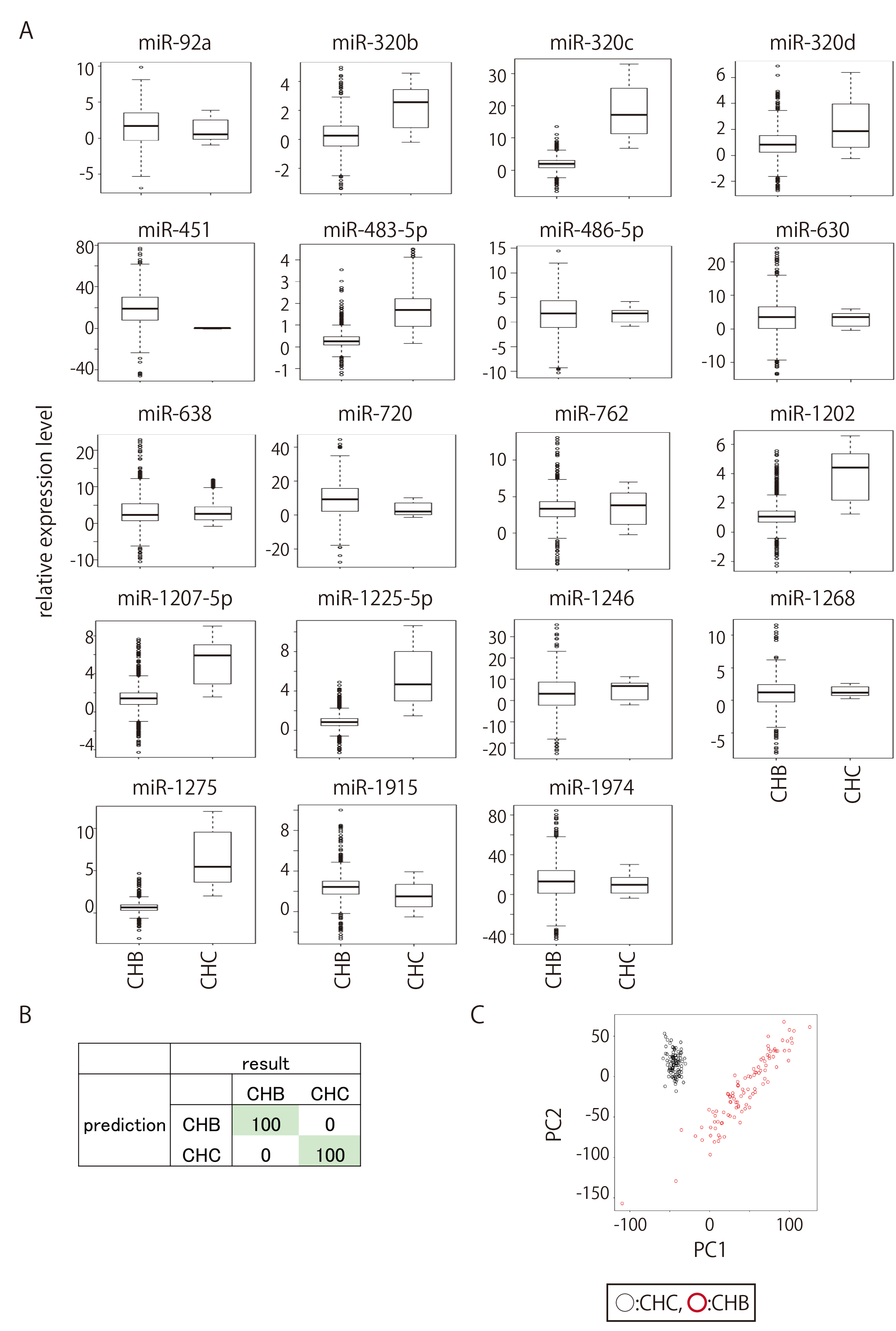

Supplement: Figure S3 — The same as Fig.3 for CHC and CHB. A. Box plot of 19 miRNAs used for the discrimination. B. Classification between CHC and CHB. Accuracy is 100%. C. The two dimensional embedding of CHB and CHC by the first and second principal component scores computed with19 selected miRNAs. (TIF) [file pone.0048366.s003.tif]

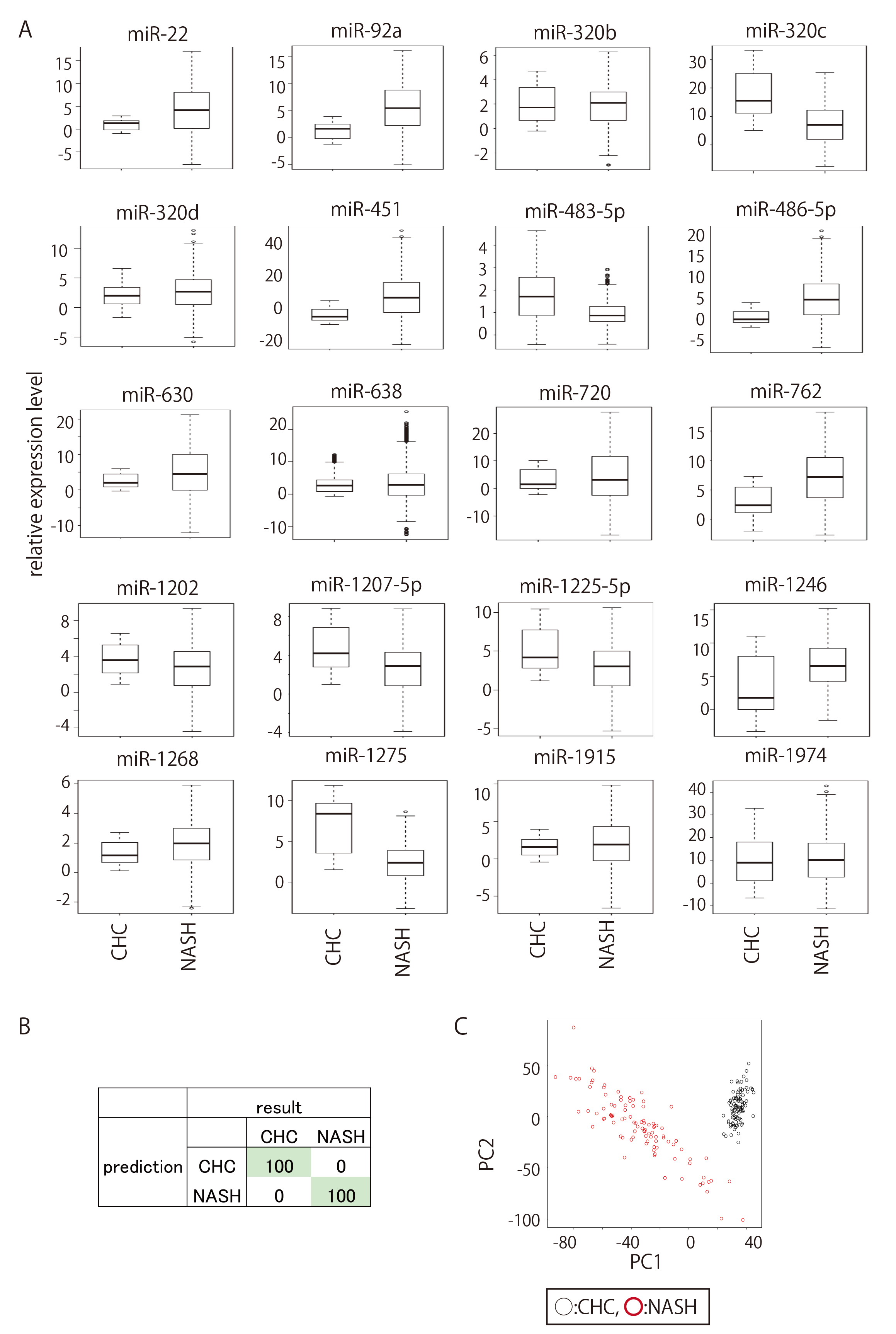

Supplement: Figure S4 — The same as Fig.S3 for CHC and NASH. A. Box plots of 20 miRNAs used for the discrimination. B. Classification between CHC and NASH. Accuracy is 100%. C. Two dimensional embedding of CHC and NASH by the first and second principal component scores computed with 19 selected miRNAs (TIF) [file pone.0048366.s004.tif]

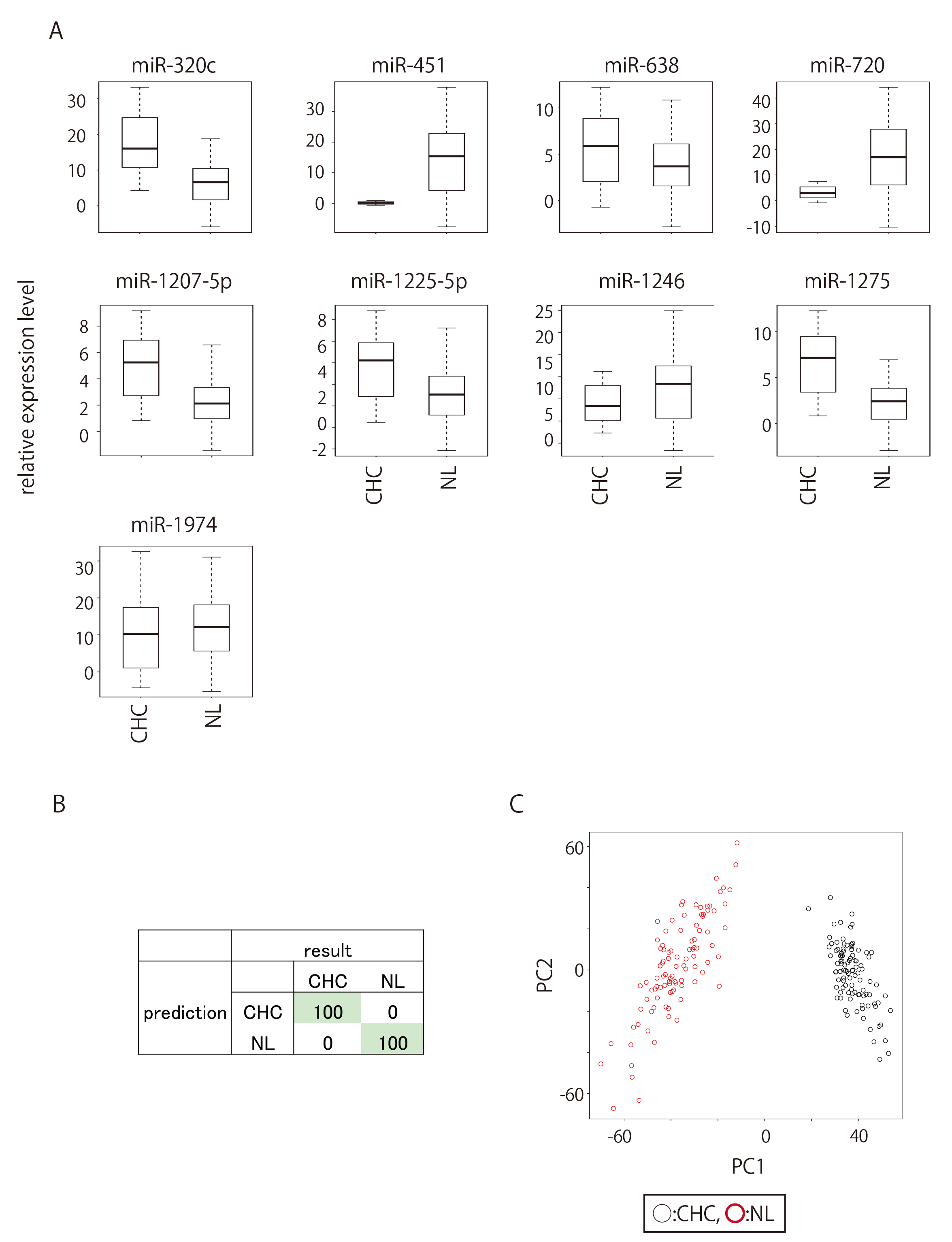

Supplement: Figure S5 — The same as Fig.S3 for CHC and NL. A. Box plots of 9 miRNAs used for the discrimination. B. Classification between CHC and NL. Accuracy is 100%. C. Two dimensional embedding of CHC and NL by the first and second principal component scores computed with 9 selected miRNAs (TIF) [file pone.0048366.s005.tif]

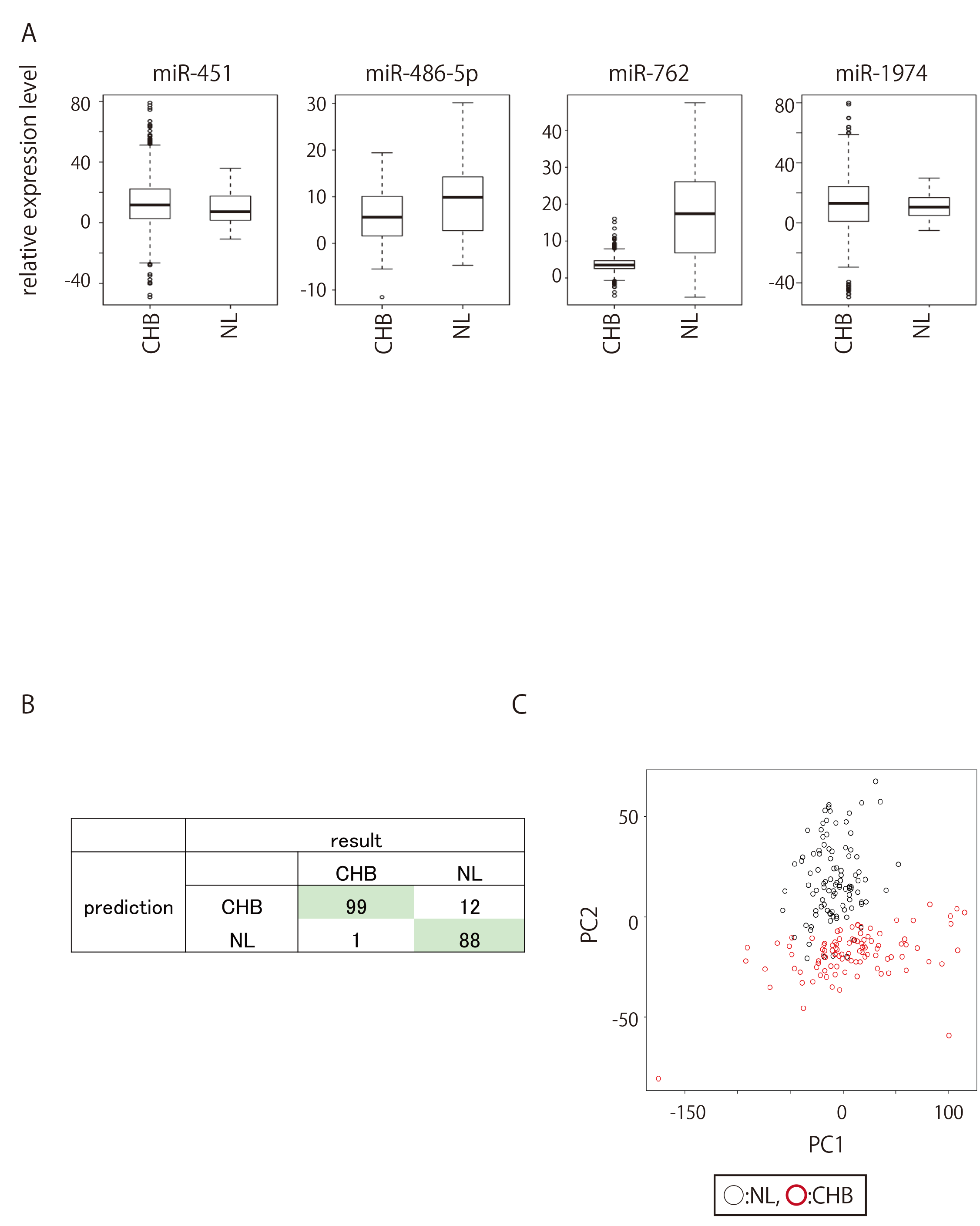

Supplement: Figure S6 — The same as Fig.S3 for CHB and NL. A. Box plots of 4 miRNAs used for the discrimination. B. Classification between CHB and NL. Accuracy is 93.5%. C. Two dimensional embedding of CHB and NL by the first and second principal component scores computed with 4 selected miRNAs (TIF) [file pone.0048366.s006.tif]

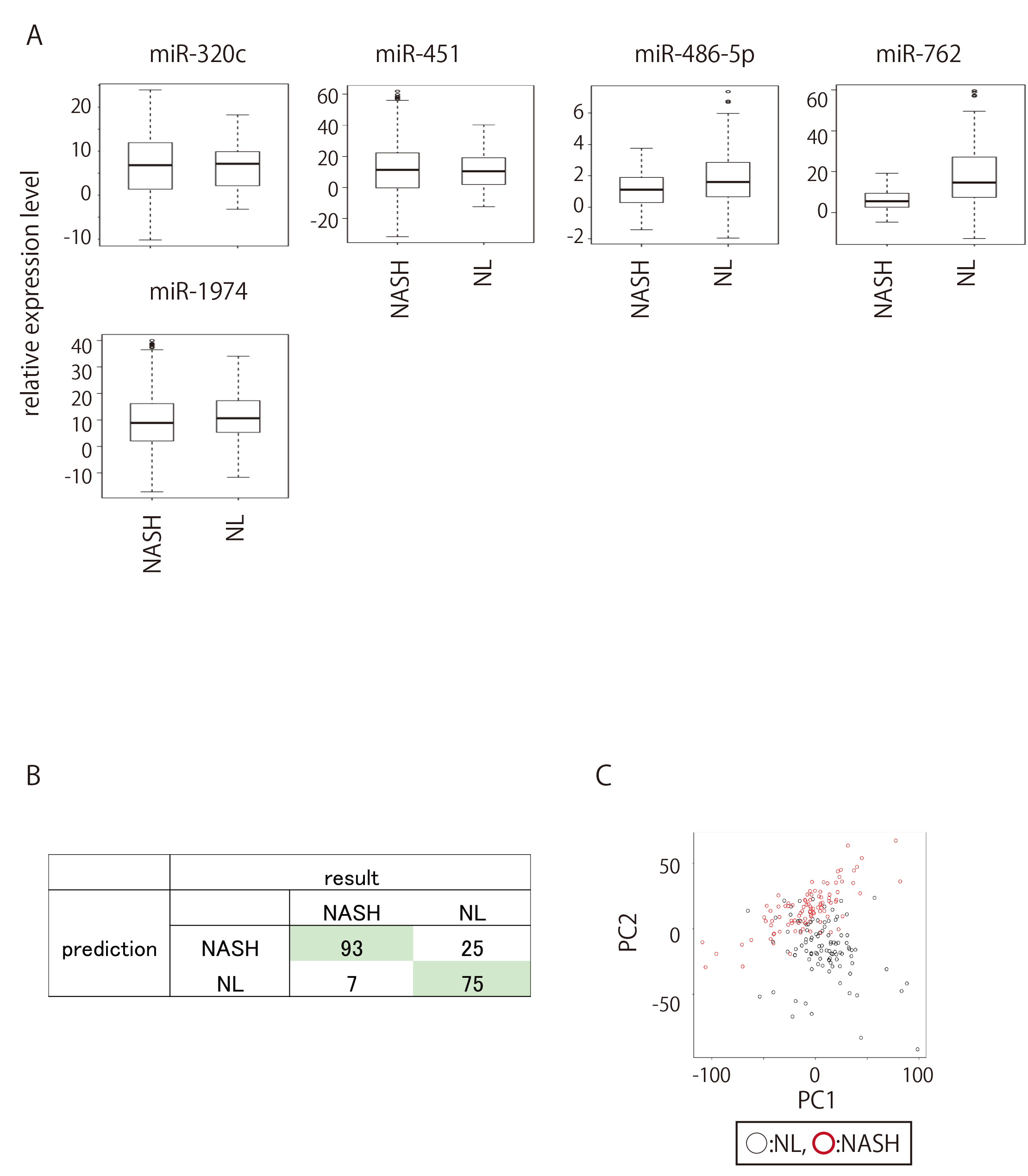

Supplement: Figure S7 — The same as Fig.S3 for NASH and NL. A. Box plots of 5 miRNAs used for the discrimination. B. Distinguishing between NASH and NL with 84.0% accuracy. C. Two dimensional embedding of NASH and NL by the first and second principal component scores computed with 5 selected miRNAs (TIF) [file pone.0048366.s007.tif]

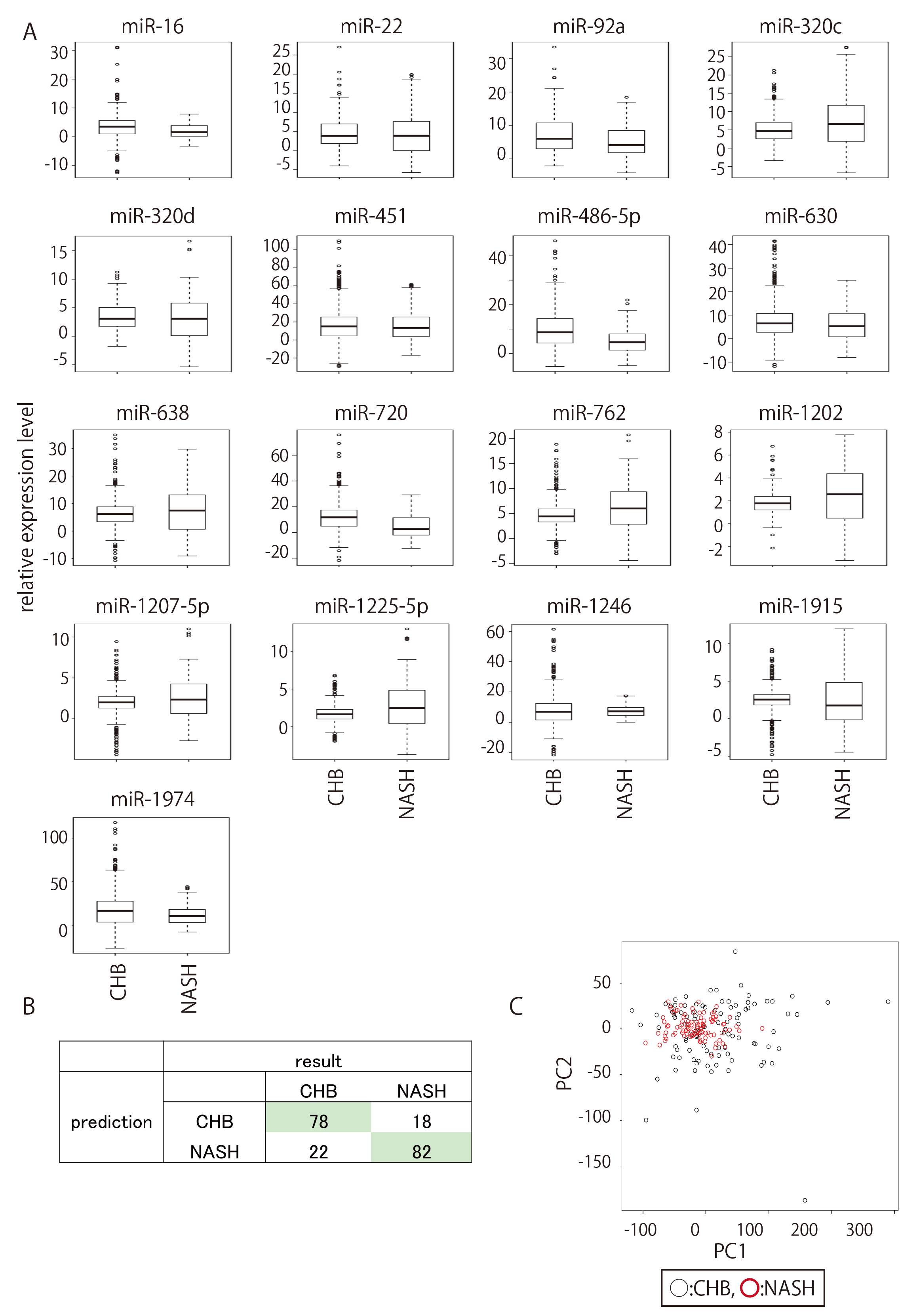

Supplement: Figure S8 — The same as Fig.S3 for CHB and NASH pair. A. Box plots of 17 miRNAs used for the discrimination. B. Distinguishing between CHB and NASH with 80.0% accuracy. C. Two dimensional embedding of CHB and NASH by the first and second principal component scores computed with 17 selected miRNAs (TIF) [file pone.0048366.s008.tif]

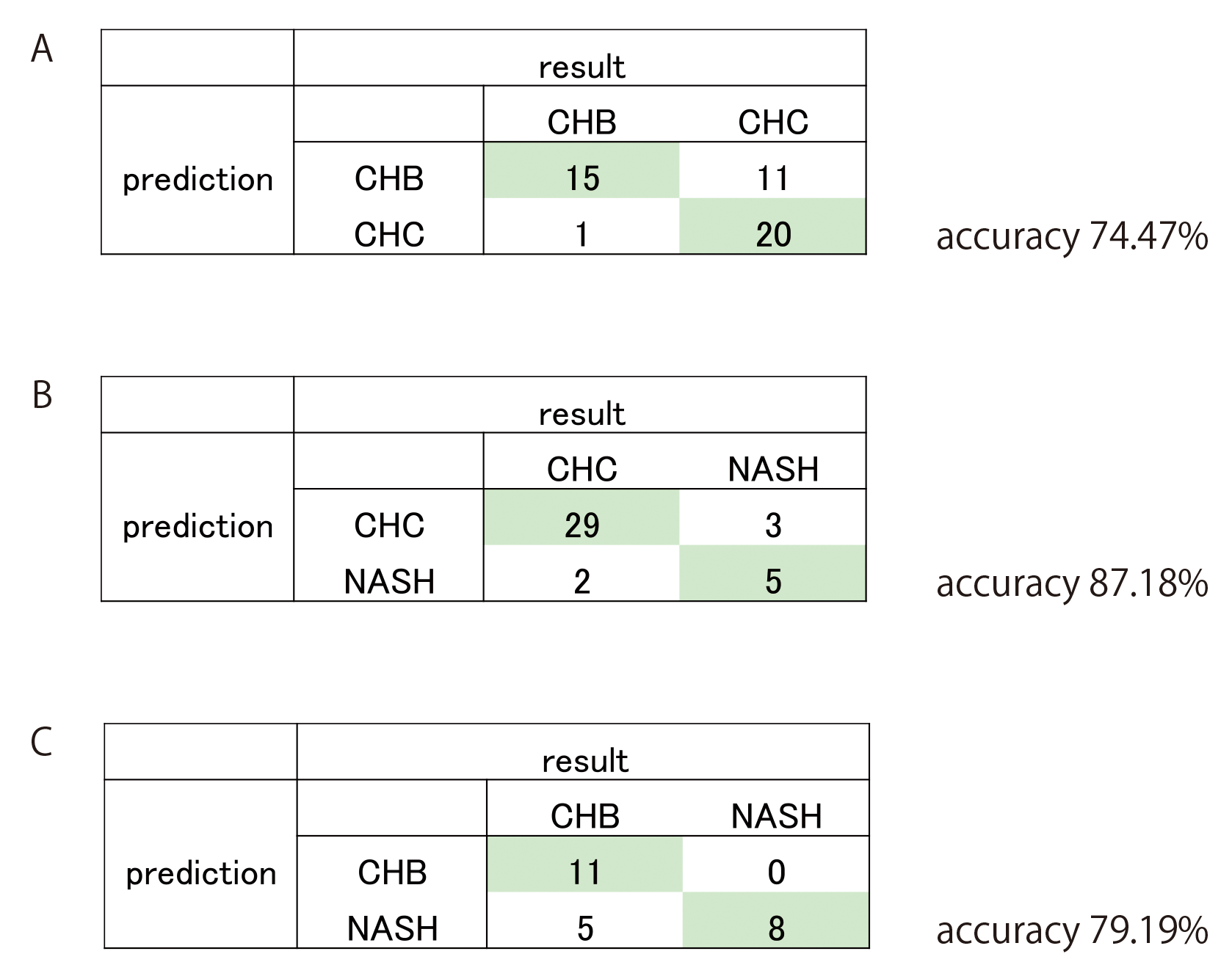

Supplement: Figure S9 — Classification of the independent sample using semi-supervised learning based on the labels in the original cohort. A. Classifying CHB and CHC. Accuracy is 74.47%. B. Classifying CHC and NASH. Accuracy is 87.18%. C. Classifying CHB and NASH. Accuracy is 79.19%. (TIF) [file pone.0048366.s009.tif]

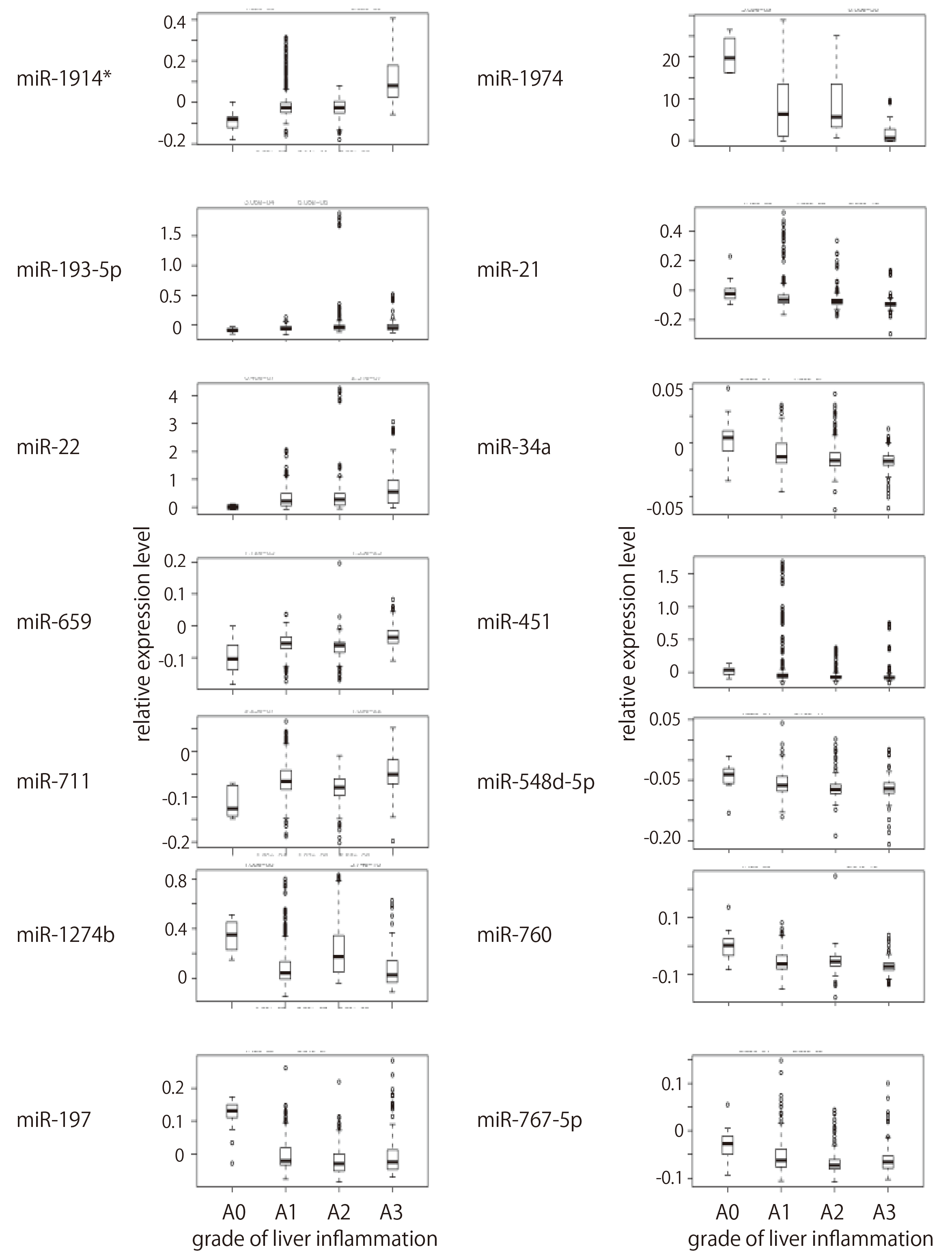

Supplement: Figure S10 — miRNA expression pattern that correlated with the changes in clinical background. miRNAs that were differentially expressed according to the grade of liver inflammation (TIF) [file pone.0048366.s010.tif]

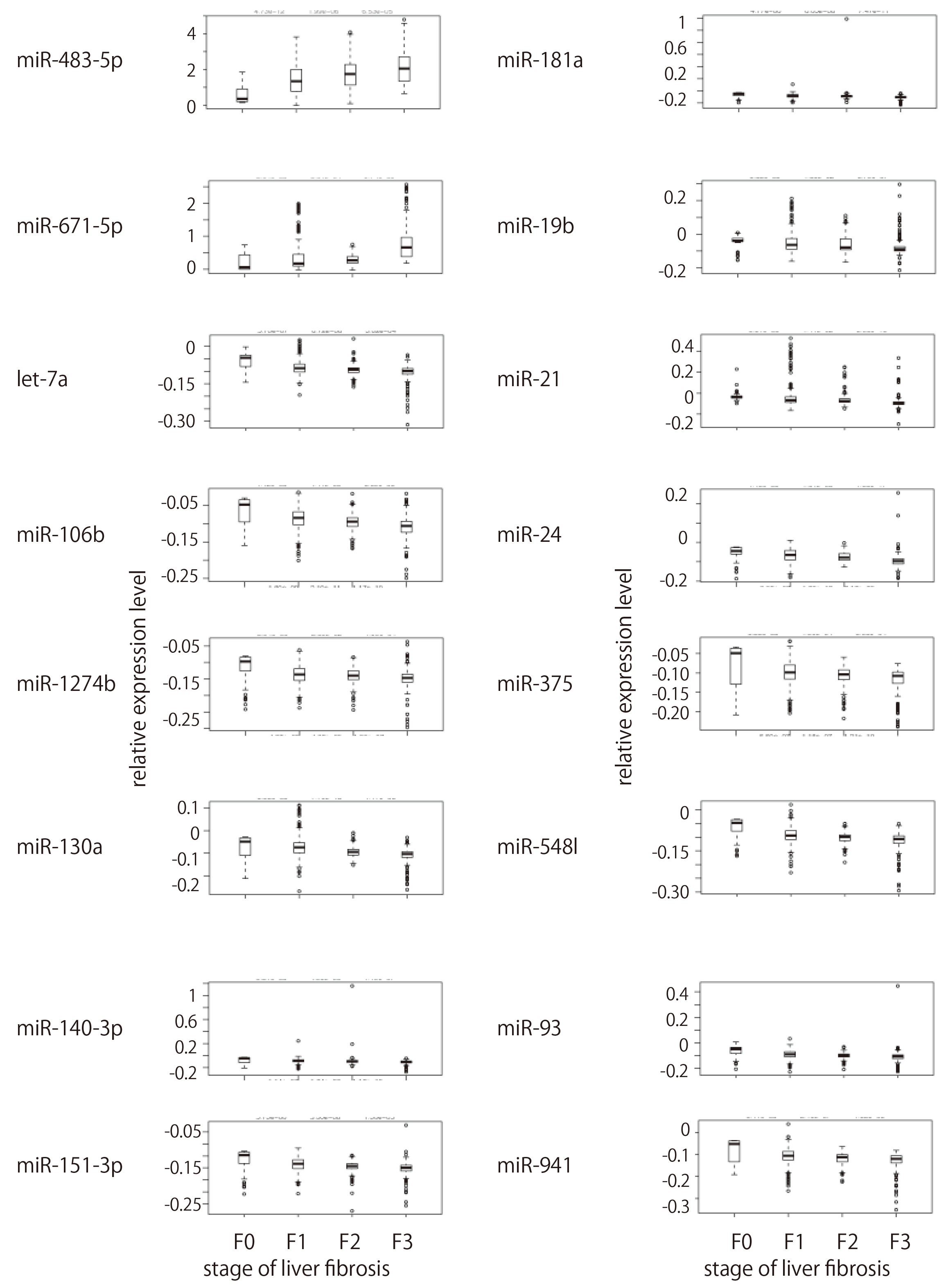

Supplement: Figure S11 — miRNA expression pattern that correlated with the changes in clinical background. miRNAs that were differentially expressed according to liver fibrosis stage (TIF) [file pone.0048366.s011.tif]

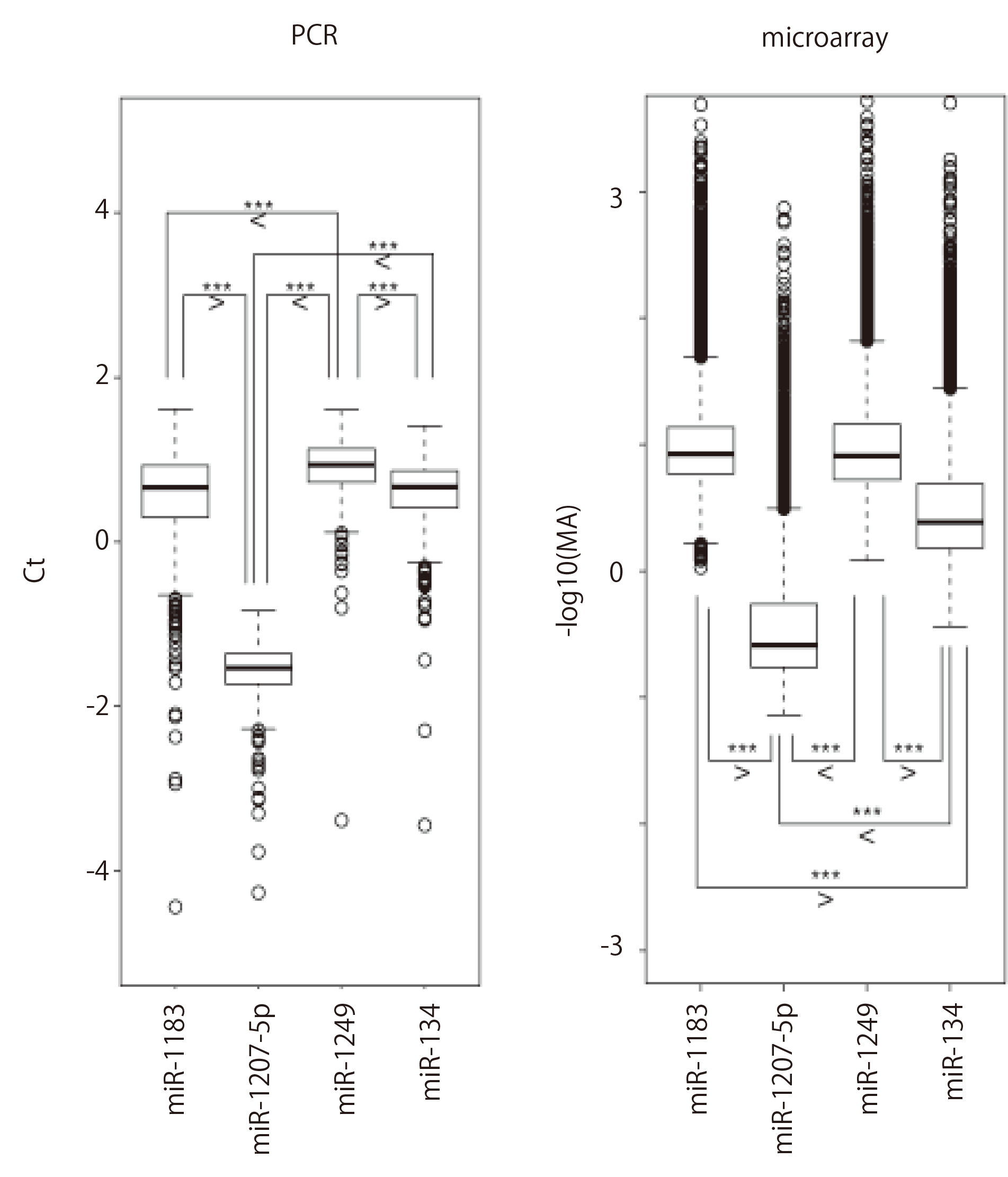

Supplement: Figure S12 — Real-time qPCR validation of microarray analysis “in silico” resampling for disease discrimination studies reflected by BMI. The result of microarray expression analysis of four miRNAs was reproduced using real-time PCR analysis. Pairs with p<0.001 are marked by “***”. (TIF) [file pone.0048366.s012.tif]

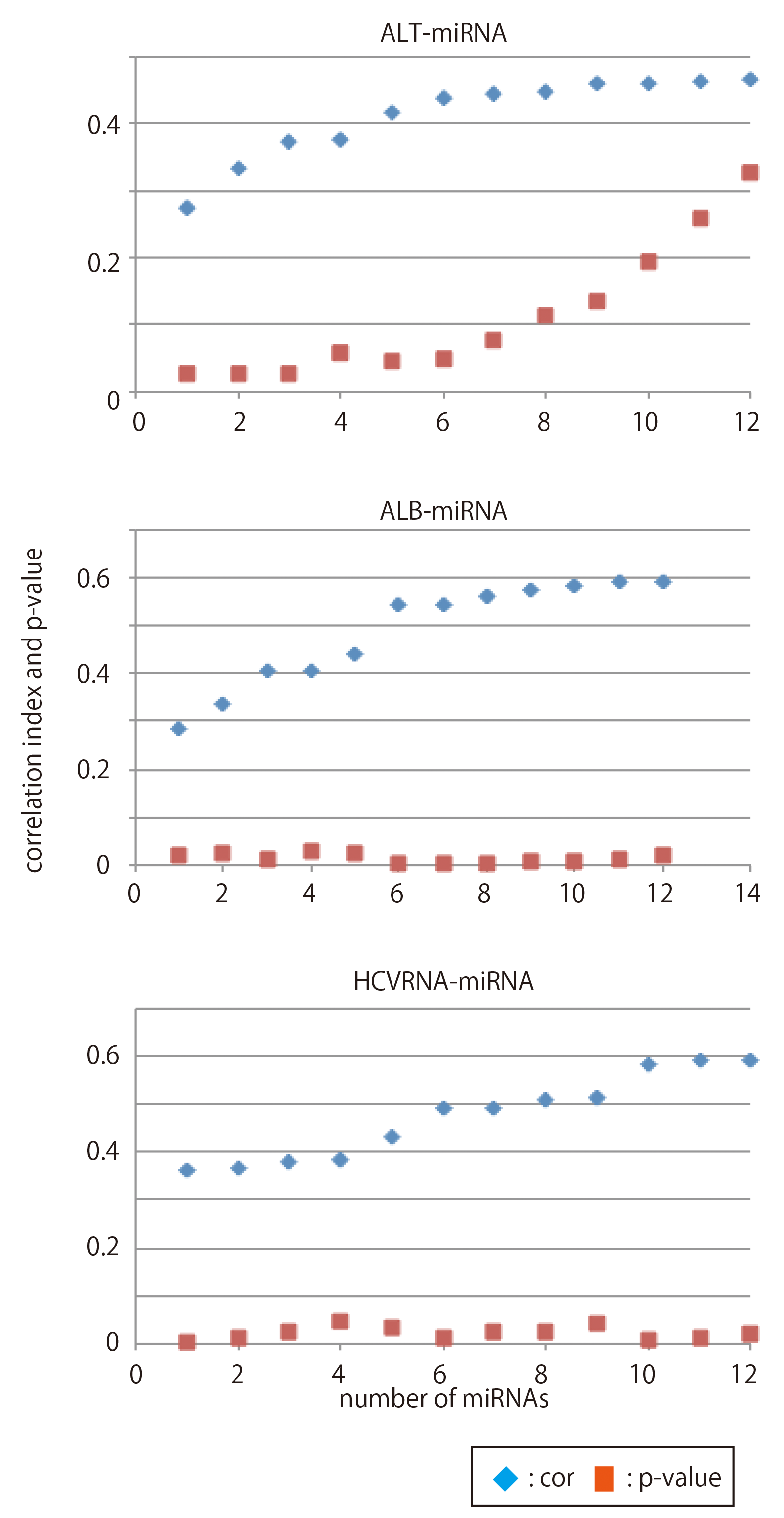

Supplement: Figure S13 — The relationship between the expression levels of several miRNAs and serum ALT, albumin, HCVRNA, respectively. Horizontal axis shows the number of miRNAs used in the analysis. Vertical axis shows the correlation index and p-values. (TIF) [file pone.0048366.s013.tif]

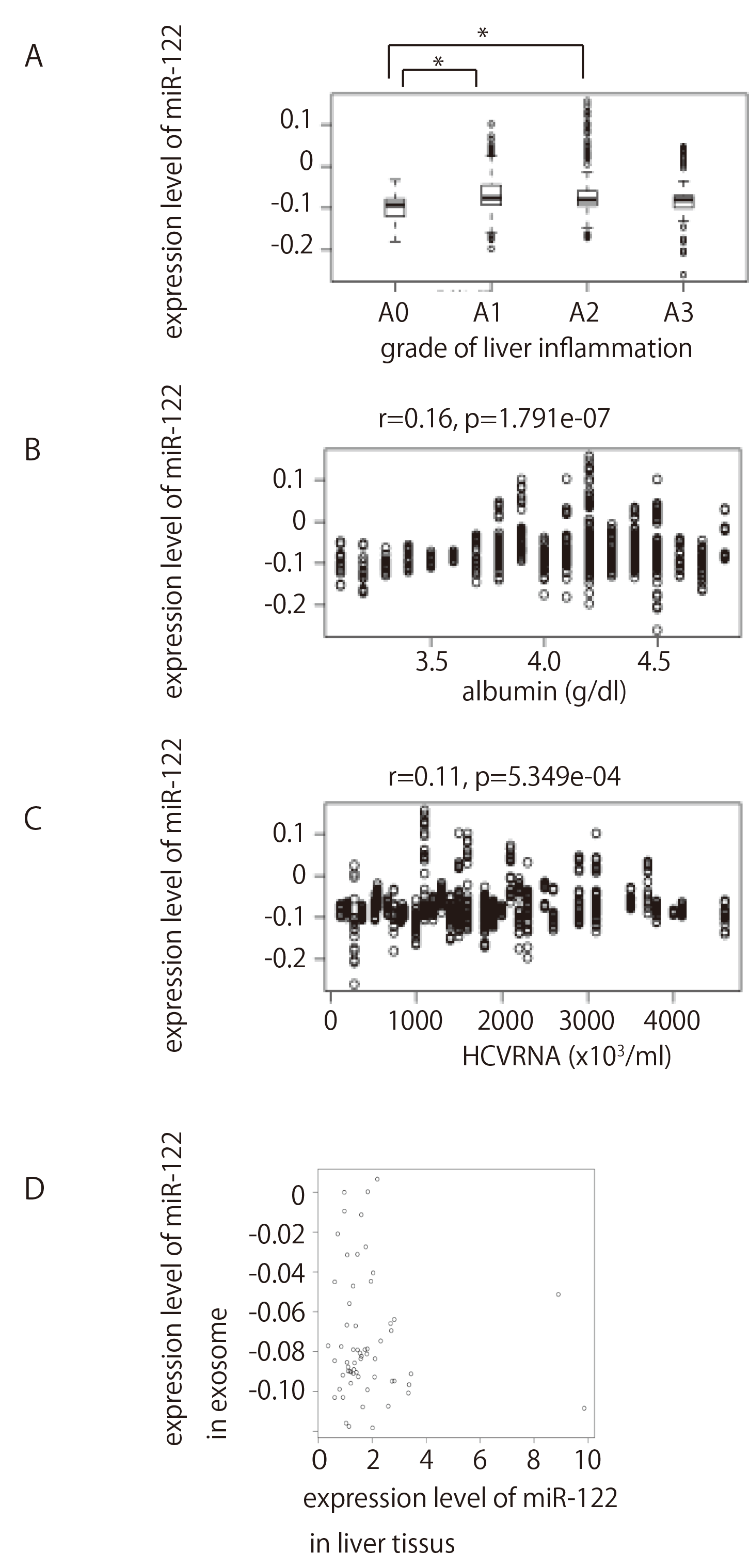

Supplement: Figure S14 — Summary of the relationship between the expression level of miR-122 and several clinical features. A. Expression level of miR-122 positively correlated with an increase in liver inflammatory grade. Asterisk denotes significant differences of p<0.05. B. Expression level of miR-122 positively correlated with the serum level of albumin. C. Expression level of miR-122 positively correlated with the amount of serum HCVRNA. D. Expression level of miR-122 in exosome rich fraction did not significantly correlate with that in liver tissues. (TIF) [file pone.0048366.s014.tif]
