# Supplementary material for: Comprehensive miRNA Expression Analysis in Peripheral Blood Can Diagnose Liver Disease
Source: PLoS One. 2012 Oct 31;7(10):e48366. doi: 10.1371/journal.pone.0048366 (PMC3485241; doi:10.1371/journal.pone.0048366)
Supplement: Table S1 — The list of miRNAs used for classifying arbitrary 2 groups and 4 groups, and their p-values. (DOCX) [file pone.0048366.s015.docx]

Table S1. List of miRNAs used for classifying each group, and their p-values

| **2 groups** |  |  |  |  |  |  |
| --- | --- | --- | --- | --- | --- | --- |
| **CHC/NL** |  | **NASH/NL** |  | **CHB/NL** |  |  |
| **miRNA** | **p-value** | **miRNA** | **p-value** | **miRNA** | **p-value** |  |
| miR-1225-5p | 6.63E-77 | miR-762 | 4.30E-12 | miR-762 | 8.09E-07 |  |
| miR-1275 | 3.30E-32 | miR-320c | 1.57E-02 | miR-451 | 7.58E-03 |  |
| miR-638 | 3.83E-01 | miR-451 | 1.48E-03 | miR-1974 | 8.74E-04 |  |
| miR-762 | 5.44E-54 | miR-1974 | 1.32E-01 | miR-486-5p | 5.95E-03 |  |
| miR-320c | 5.48E-123 | miR-486-5p | 2.10E-02 |  |  |  |
| miR-451 | 2.72E-176 |  |  |  |  |  |
| miR-1974 | 5.32E-05 |  |  |  |  |  |
| miR-1207-5p | 2.14E-94 |  |  |  |  |  |
| miR-1246 | 9.98E-01 |  |  |  |  |  |
|  |  |  |  |  |  |  |
| **CHC/CHB** |  | **CHC/NASH** |  | **CHB/NASH** |  |  |
| **miRNA** | **p-value** | **miRNA** | **p-value** | **miRNA** | **p-value** |  |
| miR-1225-5p | 5.30E-31 | miR-1225-5p | 4.16E-48 | miR-1225-5p | 1.92E-03 |  |
| miR-1275 | 8.51E-11 | miR-1275 | 4.66E-22 | miR-638 | 6.35E-01 |  |
| miR-638 | 1.91E-01 | miR-638 | 1.96E-02 | miR-762 | 7.82E-02 |  |
| miR-762 | 7.36E-01 | miR-762 | 3.00E-08 | miR-320c | 1.53E-04 |  |
| miR-320c | 3.30E-34 | miR-320c | 1.44E-71 | miR-486-5p | 2.10E-03 |  |
| miR-1202 | 1.84E-25 | miR-1202 | 2.70E-26 | miR-451 | 9.26E-01 |  |
| miR-486-5p | 1.41E-36 | miR-486-5p | 2.12E-79 | miR-1915 | 4.04E-02 |  |
| miR-451 | 3.70E-41 | miR-451 | 2.10E-102 | miR-1974 | 9.66E-03 |  |
| miR-1974 | 3.53E-06 | miR-1974 | 5.97E-05 | miR-630 | 6.64E-01 |  |
| miR-1915 | 2.93E-14 | miR-1915 | 1.33E-10 | miR-1207-5p | 3.19E-02 |  |
| miR-630 | 2.22E-23 | miR-630 | 1.84E-30 | miR-16 | 4.65E-02 |  |
| miR-483-5p | 1.64E-32 | miR-483-5p | 3.05E-52 | miR-720 | 5.56E-09 |  |
| miR-1207-5p | 7.33E-27 | miR-1207-5p | 1.91E-02 | miR-1246 | 1.54E-02 |  |
| miR-720 | 1.59E-16 | miR-720 | 4.43E-04 | miR-320d | 2.26E-01 |  |
| miR-1246 | 9.32E-01 | miR-1246 | 1.64E-05 | miR-92a | 9.43E-01 |  |
| miR-320d | 9.56E-01 | miR-320d | 8.79E-02 | miR-22 | 5.78E-01 |  |
| miR-320b | 1.65E-02 | miR-320b | 9.33E-07 | miR-1202 | 4.84E-03 |  |
| miR-92a | 9.20E-30 | miR-92a | 2.51E-78 |  |  |  |
| miR-1268 | 6.30E-05 | miR-1268 | 5.55E-08 |  |  |  |
|  |  | miR-22 | 5.50E-55 |  |  |  |
| **4 groups** |  |  |  |  |  |  |
|  | **CHC/CHB** | **CHC/NASH** | **NASH/NL** | **CHC/NL** | **CHB/NL** | **CHB/NASH** |
| **miRNA** | **p-value** | **p-value** | **p-value** | **p-value** | **p-value** | **p-value** |
| miR-1225-5p | 5.30E-31 | 4.16E-48 | 2.91E-01 | 6.63E-77 | 7.84E-12 | 1.92E-03 |
| miR-1275 | 8.51E-11 | 4.66E-22 | 5.32E-01 | 3.30E-32 | 4.01E-06 | 1.13E-04 |
| miR-638 | 1.91E-01 | 1.96E-02 | 3.18E-02 | 3.83E-01 | 4.26E-02 | 6.35E-01 |
| miR-762 | 7.36E-01 | 3.00E-08 | 4.30E-12 | 5.44E-54 | 8.09E-07 | 7.82E-02 |
| miR-320c | 3.30E-34 | 1.44E-71 | 1.57E-02 | 5.48E-123 | 5.31E-05 | 1.53E-04 |
| mR-451 | 3.70E-41 | 2.10E-102 | 1.48E-03 | 2.72E-176 | 7.58E-03 | 9.26E-01 |
| miR-1974 | 3.53E-06 | 5.97E-05 | 1.32E-01 | 5.32E-05 | 8.74E-04 | 9.66E-03 |
| miR-630 | 2.22E-23 | 1.84E-30 | 8.90E-05 | 2.99E-32 | 1.73E-04 | 6.64E-01 |
| miR-1207-5p | 7.33E-27 | 1.91E-54 | 4.59E-01 | 2.14E-94 | 1.17E-03 | 3.19E-02 |
| miR-720 | 1.59E-16 | 4.43E-04 | 1.07E-01 | 1.09E-02 | 3.94E-13 | 5.56E-09 |
| miR-1246 | 9.32E-01 | 1.64E-05 | 4.68E-03 | 9.98E-01 | 8.08E-01 | 1.54E-02 |
| miR-486-5p | 1.41E-36 | 2.12E-79 | 5.22E-02 | 6.90E-142 | 5.95E-03 | 2.10E-03 |
