# Supplementary material for: Comprehensive miRNA Expression Analysis in Peripheral Blood Can Diagnose Liver Disease
Source: PLoS One. 2012 Oct 31;7(10):e48366. doi: 10.1371/journal.pone.0048366 (PMC3485241; doi:10.1371/journal.pone.0048366)
Supplement: Table S2 — Significantly differentially expressed miRNAs according liver inflammation grade and liver fibrotic stage. (DOCX) [file pone.0048366.s016.docx]

Table S2. Significantly differentially expressed miRNAs according to liver inflammation grade and fibrotic stage

| **liver inflammation** |  |  |  |  |  |  |
| --- | --- | --- | --- | --- | --- | --- |
| **miRNA** | **A0 vs. A1** | **A0 vs. A2** | **A0 vs. A3** | **A1 vs. A2** | **A1 vs. A3** | **A2 vs. A3** |
| down |  |  |  |  |  |  |
| miR-1274b | 1.08E-08 | 1.00E-03 | 2.30E-07 | NS | 8.89E-03 | 5.74E-16 |
| miR-197 | 1.12E-09 | 4.19E-10 | 8.75E-08 | 3.54E-07 | 3.22E-02 | NS |
| miR-1974 | 5.89E-09 | 1.52E-08 | 2.89E-11 | NS | 6.66E-29 | 8.08E-38 |
| miR-21 | 1.42E-03 | 6.50E-05 | 1.81E-07 | 1.58E-05 | 1.28E-20 | 5.08E-12 |
| miR-34a | 3.92E-04 | 8.11E-06 | 9.52E-07 | 7.50E-07 | 5.63E-07 | NS |
| miR-451 | 1.21E-03 | 6.27E-05 | 1.13E-04 | 1.29E-10 | 6.30E-08 | NS |
| miR-548d-5p | 4.22E-04 | 1.42E-06 | 6.86E-06 | 6.72E-11 | 2.05E-05 | NS |
| miR-760 | 1.12E-06 | 1.38E-06 | 1.81E-08 | NS | 8.33E-06 | 2.84E-13 |
| miR-767-3p | 2.29E-04 | 3.82E-06 | 4.10E-05 | 2.50E-09 | 1.25E-02 | NS |
| up |  |  |  |  |  |  |
| miR-1914* | 1.62E-08 | 9.82E-08 | 7.14E-11 | NS | 2.88E-38 | 3.52E-39 |
| miR-193a-5p | 3.06E-04 | 6.56E-06 | 2.60E-05 | 6.08E-06 | 3.82E-04 | NS |
| miR-22 | 6.48E-07 | 1.77E-07 | 9.89E-08 | NS | 3.30E-12 | 2.51E-07 |
| miR-659 | 7.72E-05 | 1.97E-03 | 3.48E-07 | NS | 4.46E-14 | 1.55E-25 |
| miR-711 | 9.25E-07 | 1.89E-04 | 1.93E-08 | NS | 5.58E-06 | 1.68E-22 |
| **liver fibrosis** |  |  |  |  |  |  |
| **miRNA** | **F0 vs. F1** | **F0 vs. F2** | **F0 vs. F3** | **F1 vs. F2** | **F1 vs. F3** | **F2 vs. F3** |
| down |  |  |  |  |  |  |
| let-7a | 5.70E-07 | 4.07E-11 | 2.10E-12 | 8.72E-06 | 3.08E-12 | 3.82E-04 |
| miR-106b | 1.12E-05 | 1.89E-09 | 2.13E-12 | 4.41E-08 | 1.17E-19 | 6.69E-08 |
| miR-1274a | 2.94E-06 | 4.37E-09 | 4.66E-09 | 2.80E-02 | 1.33E-07 | 1.99E-04 |
| miR-130a | 3.95E-03 | 7.13E-07 | 2.24E-09 | 7.70E-15 | 3.07E-26 | 1.17E-06 |
| miR-140-3p | 9.21E-05 | 1.14E-06 | 1.64E-08 | 4.93E-06 | 1.16E-19 | 1.19E-07 |
| miR-151-3p | 5.73E-06 | 2.90E-10 | 1.40E-10 | 5.30E-08 | 4.86E-17 | 1.30E-05 |
| miR-181a | 4.77E-06 | 6.05E-09 | 3.38E-11 | 6.03E-06 | 9.73E-21 | 7.47E-11 |
| miR-19b | 3.55E-03 | 2.19E-03 | 6.22E-09 | 1.26E-02 | 9.82E-15 | 8.79E-07 |
| miR-21 | 8.31E-08 | 1.08E-10 | 4.02E-19 | 1.41E-02 | 1.29E-22 | 3.03E-18 |
| miR-24 | 1.16E-03 | 3.92E-07 | 1.20E-12 | 1.64E-05 | 6.17E-29 | 1.63E-17 |
| miR-548l | 2.92E-07 | 1.82E-11 | 1.32E-12 | 3.31E-07 | 8.82E-15 | 1.50E-04 |
| miR-93 | 2.91E-07 | 3.18E-10 | 5.23E-12 | 5.66E-07 | 2.58E-15 | 1.86E-05 |
| miR-941 | 8.41E-05 | 2.08E-07 | 3.41E-08 | 2.16E-07 | 1.68E-14 | 1.32E-03 |
| up |  |  |  |  |  |  |
| miR-483-5p | 4.73E-12 | 1.07E-14 | 8.47E-20 | 1.90E-06 | 4.49E-16 | 6.53E-05 |
| miR-671-5p | 2.04E-05 | 1.64E-04 | 1.72E-15 | 3.04E-04 | 1.79E-39 | 9.74E-39 |

Abbreviation; NS, no significant difference; up, miRNAs with expression that increased with the advancement of inflammation or fibrosis; down, miRNAs with expression that decreased with the advancement of inflammation or fibrosis
