# Supplementary material for: Comprehensive miRNA Expression Analysis in Peripheral Blood Can Diagnose Liver Disease
Source: PLoS One. 2012 Oct 31;7(10):e48366. doi: 10.1371/journal.pone.0048366 (PMC3485241; doi:10.1371/journal.pone.0048366)
Supplement: Table S3 — The list of miRNAs used to obtain the maximum correlation coefficient between expression level of miRNAs, and clinical characteristics. (DOCX) [file pone.0048366.s017.docx]

Table S3. The list of miRNAs used to obtain the maximum correlation coefficient between miRNA expression level and clinical information

| #ALT |  |  |  |  |
| --- | --- | --- | --- | --- |
|  | miRNAs | cor | p-value |  |
| 1 | miR-659 | 0.27 | 2.81E-02 |  |
| 2 | miR-107 | 0.33 | 2.71E-02 |  |
| 3 | miR-484 | 0.37 | 2.79E-02 |  |
| 4 | let-7a | 0.38 | 5.88E-02 |  |
| 5 | miR-670 | 0.42 | 4.44E-02 |  |
| 6 | miR-1914* | 0.44 | 4.91E-02 |  |
| 7 | miR-451 | 0.44 | 7.53E-02 | |
| 8 | miR-21 | 0.45 | 1.14E-01 | |
| 9 | miR-134 | 0.46 | 1.35E-01 | |
| 10 | miR-1183 | 0.46 | 1.94E-01 | |
| 11 | miR-486-5p | 0.46 | 2.59E-01 | |
| 12 | miR-92a | 0.46 | 3.28E-01 | |
| #Albumin |  |  |  | |
|  | miRNAs | cor | p-value | |
| 1 | miR-151-5p | 0.28 | 2.25E-02 | |
| 2 | miR-485-5p | 0.34 | 2.64E-02 | |
| 3 | miR-15a | 0.4 | 1.31E-02 | |
| 4 | miR-23a | 0.4 | 3.05E-02 | |
| 5 | miR-188-5p | 0.44 | 2.50E-02 | |
| 6 | miR-193a-5p | 0.54 | 2.03E-03 | |
| 7 | miR-144 | 0.55 | 4.41E-03 | |
| 8 | miR-134 | 0.56 | 5.39E-03 | |
| 9 | miR-1225-5p | 0.58 | 6.05E-03 | |
| 10 | miR-1915 | 0.58 | 9.14E-03 | |
| 11 | miR-320c | 0.59 | 1.18E-02 | |
| 12 | miR-1207-5p | 0.59 | 2.04E-02 | |
| #HCVRNA |  |  |  | |
|  | miRNAs | cor | p-value | |
| 1 | miR-130a | 0.36 | 3.10E-03 | |
| 2 | miR-107 | 0.37 | 1.27E-02 | |
| 3 | miR-1229 | 0.38 | 2.45E-02 | |
| 4 | miR-1249 | 0.39 | 4.72E-02 | |
| 5 | miR-1224-5p | 0.43 | 3.26E-02 | |
| 6 | miR-2276 | 0.49 | 1.27E-02 | |
| 7 | miR-21 | 0.49 | 2.32E-02 | |
| 8 | miR-451 | 0.51 | 2.62E-02 | |
| 9 | miR-671-5p | 0.51 | 4.15E-02 | |
| 10 | miR-486-5p | 0.58 | 8.41E-03 | |
| 11 | miR-92a | 0.59 | 1.09E-02 | |
| 12 | miR-188-5p | 0.59 | 1.89E-02 | |
