# Supplementary material for: Comprehensive miRNA Expression Analysis in Peripheral Blood Can Diagnose Liver Disease
Source: PLoS One. 2012 Oct 31;7(10):e48366. doi: 10.1371/journal.pone.0048366 (PMC3485241; doi:10.1371/journal.pone.0048366)
Supplement: Table S4 — List of miRNAs with expression that corresponded in liver tissue and serum. (DOCX) [file pone.0048366.s018.docx]

Table S4. List of miRNAs with expressions that corresponded in liver tissue and serum

| miRNA | cor | p-value |
| --- | --- | --- |
| miR-122 | -0.08 | 5.13E-01 |
|  |  |  |
| miR-134 | -0.25 | 4.58E-02 |
| miR-200b | -0.26 | 4.04E-02 |
| miR-324-3p | -0.25 | 4.92E-02 |
| miR-370 | 0.28 | 2.61E-02 |
