# Supplementary material for: Comprehensive miRNA Expression Analysis in Peripheral Blood Can Diagnose Liver Disease
Source: PLoS One. 2012 Oct 31;7(10):e48366. doi: 10.1371/journal.pone.0048366 (PMC3485241; doi:10.1371/journal.pone.0048366)
Supplement: Table S5 — Clinical background of original samples and independent samples in detail. (DOCX) [file pone.0048366.s019.docx]

Table S5. Clinical background in detail; Original Samples and Independent Samples

| **Original Samples** |  |  |  |  |  |  |  |  |  |
| --- | --- | --- | --- | --- | --- | --- | --- | --- | --- |
| **code No.** | **description** | **age** | **gender** | **AST** | **ALT** | **WBC** | **PLT** | **T-BIL** | **weight** |
| OCH-103 | CHC | 54 | F | 22 | 28 | 4220 | 27.8 | 0.5 | 49 |
| OCH-105 | CHC | 67 | M | 53 | 54 | 3180 | 9.7 | 1 | 66 |
| OCH-107 | CHC | 61 | F | 31 | 41 | 5480 | 15.7 | 0.9 | 65 |
| OCH-108 | CHC | 64 | M | 24 | 18 | 5810 | 19.3 | 1 | 72 |
| OCH-109 | CHC | 53 | F | 93 | 106 | 4810 | 19.7 | 0.6 | 47 |
| OCH-110 | CHC | 63 | F | 42 | 51 | 4150 | 15.9 | 0.6 | 55 |
| OCH-111 | CHC | 73 | M | 44 | 44 | 5160 | 14.4 | 0.7 | 58.5 |
| OCH-112 | CHC | 59 | F | 59 | 44 | 7490 | 13.4 | 0.9 | 55 |
| OCH-113 | CHC | 55 | F | 28 | 23 | 4370 | 17.9 | 0.8 | 51 |
| OCH-114 | CHC | 62 | F | 73 | 84 | 3380 | 9.3 | 0.4 | 63 |
| OCH-115 | CHC | 66 | F | 47 | 53 | 6050 | 15.6 | 0.6 | 45 |
| OCH-116 | CHC | 62 | F | 71 | 85 | 4250 | 9.8 | 0.8 | 56 |
| OCH-117 | CHC | 69 | M | 57 | 60 | 3930 | 10.4 | 1.1 | 67 |
| OCH-118 | CHC | 63 | F | 105 | 124 | 4520 | 10 | 0.8 | 56.5 |
| OCH-119 | CHC | 69 | F | 79 | 73 | 3980 | 8.4 | 1.1 | 53.3 |
| OCH-120 | CHC | 62 | F | 98 | 111 | 5060 | 7.6 | 0.8 | 44 |
| OCH-122 | CHC | 56 | M | 86 | 170 | 7630 | 18.1 | 0.5 | 62 |
| OCH-123 | CHC | 68 | F | 48 | 52 | 4060 | 20.5 | 0.6 | 55 |
| OCH-124 | CHC | 66 | M | 31 | 27 | 4420 | 12.7 | 0.6 | 56 |
| OCH-125 | CHC | 68 | M | 22 | 22 | 5290 | 15.6 | 0.4 | 65 |
| OCH-126 | CHC | 63 | F | 33 | 36 | 5460 | 12.1 | 0.4 | 53 |
| OCH-130 | CHC | 69 | M | 27 | 20 | 6430 | 16.7 | 0.5 | 58 |
| OCH-131 | CHC | 60 | M | 32 | 35 | 6540 | 16.2 | 0.7 | 59.2 |
| OCH-132 | CHC | 64 | M | 52 | 83 | 5090 | 15.5 | 0.5 | 70.8 |
| OCH-136 | CHC | 25 | F | 24 | 27 | 5880 | 18.1 | 0.3 | 58 |
| OCH-137 | CHC | 52 | F | 25 | 30 | 3950 | 24.9 | 0.4 | 50 |
| OCH-139 | CHC | 41 | M | 16 | 22 | 6550 | 24.3 | 1.2 | 87 |
| OCH-140 | CHC | 50 | F | 73 | 78 | 4130 | 13.5 | 0.7 | 60 |
| OCH-141 | CHC | 56 | F | 26 | 23 | 4930 | 20 | 0.4 | 51 |
| OCH-142 | CHC | 50 | F | 28 | 22 | 3620 | 14.7 | 1 | 50 |
| OCH-144 | CHC | 57 | M | 57 | 71 | 3700 | 9.2 | 0.5 | 71 |
| OCH-150 | CHC | 68 | F | 26 | 25 | 5700 | 17 | 0.5 | 51 |
| OCH-152 | CHC | 61 | F | 20 | 20 | 4410 | 16.7 | 0.7 | 54 |
| OCH-154 | CHC | 53 | M | 34 | 56 | 4830 | 19.4 | 0.6 | 63 |
| OCH-157 | CHC | 57 | F | 33 | 43 | 3890 | 12 | 0.5 | 42 |
| OCH-158 | CHC | 51 | F | 24 | 24 | 3420 | 11.7 | 0.9 | 68 |
| OCH-159 | CHC | 60 | M | 39 | 41 | 4300 | 12.4 | 0.5 | 61.1 |
| OCH-186 | CHC | 64 | M | 75 | 85 | 3500 | 14.7 | 1.1 | 62 |
| OCH-187 | CHC | 56 | M | 38 | 63 | 4800 | 18.2 | 0.7 | 68 |
| OCH-188 | CHC | 58 | M | 28 | 41 | 4350 | 15.1 | 0.6 | 54 |
| OCH-189 | CHC | 59 | F | 33 | 28 | 3840 | 16.8 | 0.6 | 52.3 |
| OCH-190 | CHC | 60 | M | 29 | 31 | 3310 | 11.8 | 0.7 | 59 |
| OCH-195 | CHC | 57 | F | 28 | 24 | 9050 | 29.4 | 0.3 | 43 |
| OCH-204 | CHC | 67 | F | 79 | 66 | 4300 | 8.6 | 0.8 | 43 |
| OCH-207 | CHC | 63 | M | 47 | 51 | 4890 | 9.8 | 0.8 | 58 |
| OCH-208 | CHC | 70 | M | 143 | 190 | 3130 | 16 | 0.6 | 43 |
| OCH-209 | CHC | 67 | F | 37 | 52 | 5060 | 16.2 | 0.6 | 47 |
| OCH-212 | CHC | 50 | M | 34 | 30 | 8080 | 27.7 | 0.3 | 69 |
| OCH-213 | CHC | 50 | F | 28 | 28 | 6090 | 22.3 | 0.5 | 60.5 |
| OCH-217 | CHC | 63 | F | 61 | 65 | 4450 | 18.6 | 0.8 | 63 |
| OCH-219 | CHC | 61 | M | 86 | 98 | 3850 | 8.6 | 0.5 | 54.5 |
| OCH-220 | CHC | 70 | F | 143 | 129 | 5310 | 14.2 | 0.7 | 45 |
| OCH-221 | CHC | 60 | F | 30 | 27 | 6610 | 27.5 | 0.4 | 59.5 |
| OCH-222 | CHC | 43 | M | 31 | 50 | 8010 | 25.6 | 0.4 | 73 |
| OCH-223 | CHC | 46 | F | 19 | 21 | 5910 | 30.6 | 0.5 | 48 |
| OCH-228 | CHC | 58 | M | 51 | 77 | 6920 | 13.4 | 0.4 | 60.7 |
| OCH-229 | CHC | 60 | M | 73 | 37 | 3130 | 9.7 | 0.6 | 51.4 |
| OCH-231 | CHC | 56 | M | 57 | 78 | 5850 | 27.8 | 0.6 | 61 |
| OCH-233 | CHC | 52 | M | 28 | 40 | 4840 | 18 | 0.8 | 65 |
| OCH-234 | CHC | 57 | F | 112 | 204 | 8150 | 16.5 | 0.6 | 68 |
| OCH-236 | CHC | 69 | M | 24 | 35 | 3160 | 14 | 0.6 | 54.7 |
| OCH-238 | CHC | 74 | M | 78 | 81 | 8620 | 17.4 | 1.2 | 79.5 |
| OCH-251 | CHC | 66 | M | 34 | 20 | 7740 | 19.8 | 0.5 | 60 |
| OCH-258 | CHC | 58 | F | 100 | 110 | 6490 | 29.4 | 0.9 | 55 |
|  |  |  |  |  |  |  |  |  |  |
| **code No.** | **description** | **age** | **gender** | **AST** | **ALT** | **WBC** | **PLT** | **T-BIL** | **weight** |
| B001 | CHB | 56 | F | 58 | 56 | 2970 | 13.8 | 1 | 57 |
| B002 | CHB | 28 | M | 147 | 395 | 4070 | 11.6 | 0.5 | 55 |
| B003 | CHB | 43 | F | 24 | 20 | 5650 | 9.9 | 1.3 | 58 |
| B004 | CHB | 60 | M | 104 | 200 | 6290 | 23.9 | 0.5 | 65 |
|  |  |  |  |  |  |  |  |  |  |
| **code No.** | **description** | **age** | **gender** | **AST** | **ALT** | **WBC** | **PLT** | **T-BIL** | **weight** |
| N001 | NASH | 68 | F | 38 | 39 | 4830 | 34.5 | 0.8 | 54 |
| N002 | NASH | 59 | F | 62 | 108 | 6010 | 14.6 | 1.3 | 50 |
| N003 | NASH | 36 | M | 40 | 90 | 4960 | 23.1 | 0.7 | 76 |
| N004 | NASH | 58 | M | 60 | 134 | 10340 | 32.4 | 0.5 | 70 |
| N005 | NASH | 55 | M | 26 | 26 | 5050 | 10.2 | 0.7 | 65 |
| N006 | NASH | 45 | F | 45 | 57 | 5130 | 30.9 | 0.8 | 55 |
| N007 | NASH | 64 | M | 77 | 70 | 6770 | 21.5 | 0.4 | 75 |
| NASH26 | NASH | 34 | M | 48 | 89 | 7130 | 29.4 | 0.8 | 95 |
| NASH27 | NASH | 57 | M | 38 | 65 | 8260 | 19.5 | 1 | 102 |
| NASH28 | NASH | 69 | M | 52 | 56 | 5810 | 20.2 | 0.4 | 75 |
| NASH29 | NASH | 31 | M | 50 | 124 | 5831 | 35.2 | 0.9 | 85 |
| NASH31 | NASH | 52 | M | 18 | 36 | 5000 | 24.8 | 0.8 | 72 |
|  |  |  |  |  |  |  |  |  |  |
| **code No.** | **description** | **age** | **gender** |  |  |  |  |  |  |
| NL1 | NL | 43 | F |  |  |  |  |  |  |
| NL3 | NL | 47 | F |  |  |  |  |  |  |
| NL4 | NL | 56 | F |  |  |  |  |  |  |
| NL5 | NL | 27 | M |  |  |  |  |  |  |
| NL6 | NL | 30 | M |  |  |  |  |  |  |
| NL7 | NL | 34 | F |  |  |  |  |  |  |
| NL8 | NL | 35 | M |  |  |  |  |  |  |
| NL9 | NL | 39 | F |  |  |  |  |  |  |
| NL10 | NL | 39 | M |  |  |  |  |  |  |
| NL11 | NL | 51 | F |  |  |  |  |  |  |
| NL12 | NL | 54 | F |  |  |  |  |  |  |
| NL13 | NL | 63 | M |  |  |  |  |  |  |
| NL14 | NL | 63 | M |  |  |  |  |  |  |
| NL15 | NL | 60 | M |  |  |  |  |  |  |
| NL16 | NL | 59 | M |  |  |  |  |  |  |
| NL17 | NL | 57 | M |  |  |  |  |  |  |
| NL18 | NL | 56 | M |  |  |  |  |  |  |
| NL19 | NL | 55 | M |  |  |  |  |  |  |
| NL20 | NL | 55 | F |  |  |  |  |  |  |
| NL27 | NL | 56 | F |  |  |  |  |  |  |
| NL28 | NL | 73 | M |  |  |  |  |  |  |
| NL29 | NL | 58 | F |  |  |  |  |  |  |
| NL31 | NL | 44 | F |  |  |  |  |  |  |
| NL33 | NL | 65 | M |  |  |  |  |  |  |
|  |  |  |  |  |  |  |  |  |  |
| **code No.** | **ALP** | **γGTP** | **HB** | **Albumin** | **inflammation** | **fibrosis** | **genotype** | **HCVRNA** | **outcome** |
| OCH-103 | 299 | 47 | 13.7 | 4.3 | 1 | 1 | 1B | 6.1 | SVR |
| OCH-105 | 291 | 111 | 14 | 3.2 | 2 | 3 | 1B | 6.3 | NR |
| OCH-107 | 352 | 17 | 13.5 | 4.3 | 1 | 1 | 1B | 6 | SVR |
| OCH-108 | 174 | 15 | 14.5 | 4.2 | 2 | 1 | 1B | 6.5 | SVR |
| OCH-109 | 402 | 39 | 13.4 | 4.2 | 1 | 1 | 1B | 6.1 | SVR |
| OCH-110 | 190 | 17 | 14.6 | 4.4 | 1 | 1 | 1B | 6.5 | SVR |
| OCH-111 | 231 | 17 | 13.2 | 3.6 | 3 | 3 | 1B | 6.3 | NR |
| OCH-112 | 377 | 32 | 14.2 | 3.7 | 3 | 3 | 1B | 6.2 | SVR |
| OCH-113 | 160 | 18 | 12.3 | 4.2 | 2 | 3 | 1B | 6.3 | R |
| OCH-114 | 322 | 50 | 15.1 | 4.5 | 3 | 3 | 1B | 5.4 | SVR |
| OCH-115 | 438 | 28 | 15.1 | 4.6 | 2 | 3 | 1B | 6.2 | R |
| OCH-116 | 341 | 33 | 14.4 | 4.1 | 3 | 3 | 1B | 6 | SVR |
| OCH-117 | 344 | 61 | 13.1 | 4 | 3 | 3 | 1B | 5.1 | R |
| OCH-118 | 538 | 182 | 13.4 | 3.3 | 3 | 3 | 1B | 6.2 | NR |
| OCH-119 | 359 | 65 | 13.1 | 4 | 3 | 3 | 1B | 6.2 | NR |
| OCH-120 | 295 | 42 | 13.7 | 3.8 | 3 | 3 | 1B | 6.5 | R |
| OCH-122 | 164 | 201 | 14.4 | 4.7 | 1 | 1 | 1B | 5.7 | NR |
| OCH-123 | 304 | 34 | 12.7 | 4.1 | 1 | 1 | 1B | 5.3 | NR |
| OCH-124 | 293 | 22 | 12.5 | 4.1 | 1 | 1 | 1B | 6.5 | SVR |
| OCH-125 | 215 | 17 | 14.7 | 4 | 1 | 1 | 1B | 6.3 | R |
| OCH-126 | 283 | 29 | 14.8 | 4 | 1 | 1 | 1B | 5.6 | NR |
| OCH-130 | 193 | 26 | 13.4 | 4.4 | 1 | 1 | 1B | 6.5 | SVR |
| OCH-131 | 222 | 21 | 15.5 | 4.2 | 1 | 1 | 1B | 6.5 | SVR |
| OCH-132 | 421 | 120 | 15.1 | 4.4 | 1 | 1 | 1B | 6.6 | NR |
| OCH-136 | 147 | 14 | 14.5 | 4.4 | 1 | 1 | 1B | 6.3 | SVR |
| OCH-137 | 205 | 34 | 13.8 | 4.7 | 1 | 1 | 1B | 6 | NR |
| OCH-139 | 244 | 21 | 16.3 | 4.4 | 1 | 1 | 1B | 6.6 | SVR |
| OCH-140 | 283 | 44 | 13.5 | 4.1 | 1 | 1 | 1B | 6.1 | NR |
| OCH-141 | 214 | 19 | 12 | 4.2 | 1 | 1 | 1B | 6.4 | R |
| OCH-142 | 240 | 13 | 12.4 | 4.5 | 1 | 1 | 1B | 6.5 | NR |
| OCH-144 | 257 | 84 | 12.4 | 4 | 3 | 3 | 1B | 6.3 | NR |
| OCH-150 | 245 | 12 | 12.8 | 4.5 | 1 | 1 | 1B | 5.9 | SVR |
| OCH-152 | 182 | 20 | 12.5 | 4.2 | 1 | 1 | 1B | 6.3 | R |
| OCH-154 | 215 | 29 | 14.9 | 4.3 | 1 | 1 | 1B | 6.4 | SVR |
| OCH-157 | 195 | 28 | 13.6 | 4.4 | 1 | 1 | 1B | 5.7 | SVR |
| OCH-158 | 159 | 21 | 12.5 | 3.8 | 1 | 1 | 1B | 6.7 | SVR |
| OCH-159 | 257 | 22 | 15.7 | 3.7 | 1 | 1 | 1B | 5.7 | R |
| OCH-186 | 378 | 58 | 13.7 | 3.9 | 1 | 1 | 1B | 6.3 | SVR |
| OCH-187 | 177 | 70 | 13.8 | 3.9 | 1 | 1 | 1B | 6.1 | SVR |
| OCH-188 | 137 | 25 | 14.7 | 3.8 | 1 | 1 | 1B | 6.6 | R |
| OCH-189 | 194 | 22 | 10.5 | 4.3 | 1 | 1 | 1B | 6.3 | SVR |
| OCH-190 | 139 | 11 | 14 | 3.8 | 1 | 1 | 1B | 6.3 | SVR |
| OCH-195 | 336 | 40 | 14.4 | 4.7 | 1 | 1 | 1B | 5.9 | SVR |
| OCH-204 | 366 | 122 | 11.1 | 3 | 1 | 2 | 1B | 6.1 | NR |
| OCH-207 | 302 | 49 | 13.4 | 4.2 | 2 | 2 | 1B | 6.1 | NR |
| OCH-208 | 395 | 87 | 15.2 | 3.9 | 2 | 2 | 1B | 6.2 | NR |
| OCH-209 | 227 | 23 | 11.8 | 3.4 | 2 | 2 | 1B | 6.3 | NR |
| OCH-212 | 255 | 39 | 15.7 | 4.6 | 1 | 1 | 2B | 7.2 | NR |
| OCH-213 | 176 | 29 | 12.4 | 4.6 | 1 | 0 | 1B | 6.4 | R |
| OCH-217 | 255 | 30 | 12.7 | 4.2 | 2 | 2 | 1B | 6.5 | R |
| OCH-219 | 305 | 46 | 13.9 | 4.2 | 2 | 2 | 1B | 5.9 | R |
| OCH-220 | 352 | 74 | 13.4 | 3.8 | 2 | 2 | 1B | 5.8 | R |
| OCH-221 | 238 | 18 | 12.9 | 4.5 | 2 | 2 | 1B | 6.4 | R |
| OCH-222 | 262 | 185 | 16.7 | 4.4 | 1 | 0 | 1B | 6.4 | SVR |
| OCH-223 | 177 | 31 | 14.2 | 4.1 | 0 | 0 | 1B | 5.9 | NR |
| OCH-228 | 282 | 52 | 14.9 | 3.9 | 2 | 2 | 1B | 6.4 | SVR |
| OCH-229 | 231 | 30 | 14 | 4.1 | 2 | 2 | 1B | 5.3 | SVR |
| OCH-231 | 153 | 21 | 13.3 | 3.5 | 2 | 2 | 1B | 6.3 | SVR |
| OCH-233 | 217 | 25 | 14.7 | 4.4 | 1 | 2 | 1B/2A | 6.2 | SVR |
| OCH-234 | 238 | 61 | 15.3 | 4.4 | 2 | 2 | 1B | 6.6 | SVR |
| OCH-236 | 160 | 15 | 14.3 | 4.1 | 2 | 2 | 1B | 6.2 | SVR |
| OCH-238 | 273 | 129 | 15.7 | 4.2 | 2 | 2 | 1B | 6 | SVR |
| OCH-251 | 492 | 28 | 13.6 | 4.3 | 1 | 1 | 1B | 6.7 | SVR |
| OCH-258 | 323 | 77 | 13.8 | 4.3 | 2 | 2 | 1B | 6.8 | SVR |
|  |  |  |  |  |  |  |  |  |  |
| **code No.** | **ALP** | **γGTP** | **HB** | **Albumin** | **inflammation** | **fibrosis** | **genotype** | **HBVDNA** |  |
| B001 | 223 | 55 | 13.9 | 3.7 | 3 | 3 | C | 4.2 |  |
| B002 | 221 | 24 | 15.2 | 4.1 | 2 | 1 | C | 8.1 |  |
| B003 | 194 | 31 | 14.1 | 4.8 | 1 | 1 | C | 3.5 |  |
| B004 | 255 | 199 | 14.7 | 4.2 | 2 | 2 | C | 4.3 |  |
|  |  |  |  |  |  |  |  |  |  |
| **code No.** | **ALP** | **γGTP** | **HB** | **Albumin** |  |  |  |  |  |
| N001 | 195 | 33 | 13.7 | 4.6 |  |  |  |  |  |
| N002 | 270 | 86 | 12.4 | 4.1 |  |  |  |  |  |
| N003 | 161 | 74 | 15.1 | 4.7 |  |  |  |  |  |
| N004 | 202 | 70 | 15.7 | 4.7 |  |  |  |  |  |
| N005 | 243 | 59 | 14.3 | 4 |  |  |  |  |  |
| N006 | 260 | 33 | 11.2 | 4.1 |  |  |  |  |  |
| N007 | 284 | 93 | 14.7 | 4 |  |  |  |  |  |
| N026 | 236 | 67 | 16.6 | 4.9 |  |  |  |  |  |
| N027 | 225 | 40 | 15.5 | 4.6 |  |  |  |  |  |
| N028 | 234 | 60 | 15.3 | 4.4 |  |  |  |  |  |
| N029 | 211 | 55 | 16.2 | 4.6 |  |  |  |  |  |
| N031 | 271 | 31 | 15.2 | 4.9 |  |  |  |  |  |
| **Independent samples** |  |  |  |  |  |  |  |  |  |
| **Code No.** | **description** | **age** | **gender** | **AST** | **ALT** | **WBC** | **PLT** | **T-BIL** | **weight** |
| OCH-602 | CHC | 65 | F | 39 | 36 | 4170 | 17.7 | 1 | 68 |
| OCH-003 | CHC | 68 | F | 35 | 29 | 7650 | 15 | 0.9 | 52 |
| OCH-603 | CHC | 57 | F | 34 | 33 | 4580 | 16.7 | 0.6 | 47.8 |
| OCH-604 | CHC | 66 | F | 47 | 45 | 2570 | 13.7 | 0.8 | 42 |
| OCH-605 | CHC | 58 | F | 20 | 20 | 4410 | 16.7 | 0.7 | 57.9 |
| OCH-606 | CHC | 65 | M | 28 | 38 | 4470 | 15.1 | 0.6 | 65 |
| OCH-607 | CHC | 64 | F | 65 | 46 | 3580 | 7.1 | 1.1 | 59 |
| OCH-608 | CHC | 62 | M | 41 | 40 | 4030 | 15.8 | 0.4 | 67.8 |
| OCH-609 | CHC | 73 | M | 20 | 27 | 7440 | 12.2 | 0.6 | 71 |
| OCH-610 | CHC | 46 | F | 19 | 15 | 5860 | 16.8 | 0.4 | NI |
| OCH-611 | CHC | 71 | M | 24 | 17 | 6160 | 14.5 | 1.6 | 60 |
| OCH-612 | CHC | 76 | M | 26 | 23 | 4010 | 11.9 | 0.7 | 55 |
| OCH-613 | CHC | 80 | M | 28 | 35 | 3750 | 20 | 0.7 | 65 |
| OCH-614 | CHC | 47 | F | 43 | 58 | 5960 | 21.4 | 0.3 | 42 |
| OCH-615 | CHC | 58 | M | 34 | 44 | 5600 | 15 | 0.4 | 63.4 |
| OCH-616 | CHC | 61 | M | 12 | 9 | 6030 | 20.4 | 0.6 | 52.5 |
| OCH-617 | CHC | 70 | M | 78 | 89 | 3860 | 15.8 | 1.1 | 71.7 |
| OCH-618 | CHC | 72 | M | 19 | 22 | 5410 | 17.9 | 0.7 | 65 |
| OCH-619 | CHC | 83 | M | 42 | 25 | 3670 | 12.4 | 0.5 | NI |
| OCH-206 | CHC | 75 | F | 24 | 30 | 5390 | 12.3 | 0.9 | 54 |
| OCH-620 | CHC | 59 | F | 51 | 42 | 4980 | 11.5 | 1.1 | 58 |
| OCH-215 | CHC | 68 | F | 33 | 32 | 3390 | 17.2 | 0.7 | 50 |
| OCH-621 | CHC | 54 | M | 57 | 71 | 3700 | 9.2 | 0.5 | 69.8 |
| OCH-135 | CHC | 54 | F | 23 | 21 | 3010 | 23.2 | 0.4 | 57 |
| OCH-136 | CHC | 73 | F | 23 | 26 | 3450 | 19.3 | 0.8 | 43 |
| OCH-165 | CHC | 54 | F | 48 | 47 | 5990 | 16.9 | 0.7 | 52 |
| OCH-169 | CHC | 56 | F | 105 | 112 | 6600 | 16.9 | 0.8 | 62.2 |
| OCH-622 | CHC | 69 | F | 75 | 64 | 6100 | 11.1 | 0.7 | NI |
| OCH-106 | CHC | 70 | F | 26 | 21 | 3530 | 15.1 | 0.7 | 56.5 |
| OCH-623 | CHC | 74 | M | 19 | 20 | 8750 | 20.7 | 0.4 | NI |
| OCH-144 | CHC | 50 | F | 32 | 44 | 6000 | 25.2 | 0.9 | 60 |
|  |  |  |  |  |  |  |  |  |  |
| **Code. No** | **description** | **age** | **gender** | **AST** | **ALT** | **WBC** | **PLT** | **T-BIL** | **weight** |
| B005 | CHB | 67 | F | 26 | 19 | 4090 | 16.1 | 0.9 | 51 |
| B006 | CHB | 73 | M | 23 | 21 | 4540 | 20 | 0.8 | 84 |
| B007 | CHB | 40 | F | 35 | 41 | 4310 | 26.4 | 0.9 | 53 |
| B008 | CHB | 65 | F | 19 | 15 | 3760 | 26.5 | 1.5 | NI |
| B009 | CHB | 55 | M | 23 | 16 | 4970 | 21 | 0.6 | NI |
| B010 | CHB | 66 | M | 26 | 24 | 4570 | 19.6 | 0.8 | 56 |
| B011 | CHB | 76 | M | 57 | 79 | 4690 | 11.6 | 0.9 | 65 |
| B012 | CHB | 40 | M | 17 | 22 | 3100 | 25.2 | 1.2 | 58 |
| B013 | CHB | 35 | F | 18 | 11 | 5830 | 20.7 | 0.8 | NI |
| B014 | CHB | 72 | F | 22 | 16 | 5090 | 21.6 | 0.8 | 52 |
| B015 | CHB | 33 | M | 21 | 22 | 4220 | 23.3 | 0.8 | NI |
| B016 | CHB | 42 | F | 25 | 16 | 4760 | 27.8 | 0.7 | NI |
| B017 | CHB | 72 | F | 27 | 19 | 2930 | 13.3 | 0.8 | 46 |
| B018 | CHB | 72 | F | 21 | 14 | 3840 | 13.1 | 0.7 | 42 |
| B019 | CHB | 54 | F | 24 | 16 | 3480 | 21.3 | 0.6 | 45 |
| B020 | CHB | 38 | F | 17 | 19 | 4830 | 23.7 | 1 | NI |
|  |  |  |  |  |  |  |  |  |  |
| **Code No.** | **description** | **age** | **gender** | **AST** | **ALT** | **WBC** | **PLT** | **T-BIL** | **weight** |
| NASH40 | NASH | 31 | F | 58 | 71 | 5300 | 30.3 | 0.7 | 65.05 |
| NASH41 | NASH | 61 | F | 190 | 286 | 5300 | 16.5 | 0.6 | 67.5 |
| NASH42 | NASH | 59 | F | 72 | 80 | 3000 | 11.2 | 1 | 54 |
| NASH43 | NASH | 39 | F | 87 | 135 | 6500 | 28.8 | 0.8 | 79.45 |
| NASH44 | NASH | 58 | M | 40 | 86 | 8900 | 23.8 | 0.2 | 52.95 |
| NASH45 | NASH | 61 | F | 47 | 69 | 5000 | 16.3 | 0.8 | 61.95 |
| NASH46 | NASH | 61 | M | 107 | 99 | 6300 | 17.8 | 1 | 77.85 |
| NASH47 | NASH | 68 | F | 46 | 45 | 3800 | 9.9 | 0.7 | 72.05 |
|  |  |  |  |  |  |  |  |  |  |
| **Code No.** | **ALP** | **γGTP** | **HB** | **ALB** | **inflammation** | **fibrosis** | **genotype** | **HCVRNA** | **outcome** |
| OCH-602 | 305 | 56 | 13.3 | 4.4 | NI | NI | 1B | 7.1 | NI |
| OCH-003 | 315 | 18 | 11.8 | 4 | 1 | 1 | 1B | 5.7 | relapse |
| OCH-603 | 177 | 20 | 12.3 | 4.1 | 1 | 2 | 1B | 6.1 | relapse |
| OCH-604 | 283 | 17 | 13.4 | 4.5 | NI | NI | 1B | 6.3 | NI |
| OCH-605 | 182 | 20 | 12.5 | 4.2 | 2 | 3 | 1B | 6.3 | NR |
| OCH-606 | 412 | 22 | 13.5 | 3.8 | 0 | 0 | 1B | 5.8 | NI |
| OCH-607 | 337 | 21 | 11.5 | 4.1 | 2 | 2 | 1B | 6.7 | NI |
| OCH-608 | 170 | 25 | 14.8 | 4.6 | 2 | 3 | 1B | 6.9 | NI |
| OCH-609 | 277 | 106 | 12.2 | 4.1 | NI | NI | 1B | 5.9 | NI |
| OCH-610 | 171 | 8 | 12.2 | 4.2 | NI | NI | 1B | 6.9 | NI |
| OCH-611 | 319 | 17 | 14.5 | 4.4 | NI | NI | 1B | 6.9 | NI |
| OCH-612 | 271 | 23 | 11.9 | 3.9 | NI | NI | NI | 6.8 | NI |
| OCH-613 | 278 | 34 | 11.6 | 3.8 | NI | NI | 1B | 7.2 | NI |
| OCH-614 | 225 | 169 | 14.9 | 4 | 2 | 2 | 1B | 6.3 | NR |
| OCH-615 | 324 | 53 | 14.8 | 4 | 2 | 3 | 1B | 6.4 | NI |
| OCH-616 | 309 | 18 | 15.2 | 4.7 | NI | NI | 2A | 5.8 | NP |
| OCH-617 | 233 | 149 | 14.6 | 3.3 | 1 | 1 | 2A | 5.8 | drop |
| OCH-618 | 162 | 35 | 12.5 | 4.1 | 1 | 1 | 1B | 6.8 | NP |
| OCH-619 | 325 | 13 | 10.9 | 3.3 | NI | NI | 1B | 5.8 | NP |
| OCH-206 | 200 | 32 | 12.3 | 4.2 | 1 | 1 | 1B | 6.6 | relapse |
| OCH-620 | 449 | 35 | 11.5 | 3.8 | 2 | 2 | 1B | 6.2 | NR |
| OCH-215 | 225 | 20 | 14.3 | 4.6 | 1 | 1 | 1B | 6.4 | relapse |
| OCH-621 | 257 | 84 | 12.4 | 4 | 1 | 1 | 1B | 6.3 | NR |
| OCH-135 | 152 | 8 | 12.3 | 4.3 | NI | NI | 2A | 4 | NP |
| OCH-136 | 236 | 12 | 12.5 | 4.1 | NI | NI | 1B | 6.3 | NP |
| OCH-165 | 223 | 31 | 14.5 | 4.1 | 2 | 1 | 1B | 6.5 | relapse |
| OCH-169 | 437 | 74 | 13.2 | 3.8 | NI | NI | 1B | 6.5 | NP |
| OCH-622 | 397 | 65 | 12.1 | 3.7 | NI | NI | 1B | 6.8 | NP |
| OCH-106 | 464 | 17 | 13.9 | 4.8 | 1 | 1 | 2A | 6.2 | relapse |
| OCH-623 | 223 | 11 | 13.3 | 4.2 | NI | NI | 1B | 6.1 | NP |
| OCH-144 | 182 | 36 | 13.8 | 4.8 | 1 | 1 | 1B | 6.8 | NP |
|  |  |  |  |  |  |  |  |  |  |
| **Code. No** | **ALP** | **γGTP** | **HB** | **ALB** | **inflammation** | **fibrosis** | **genotype** | **HBVDNA** |  |
| B005 | 334 | 18 | 13.2 | 4.4 | NI | NI | NI | 2.4 |  |
| B006 | 165 | 57 | 15.5 | 4.7 | NI | NI | C | 2.2 |  |
| B007 | 263 | 34 | 12.5 | 4.5 | NI | NI | C | 5.9 |  |
| B008 | 183 | 24 | 14.5 | 5 | NI | NI | C | 3.8 |  |
| B009 | 255 | 30 | 16 | 4.7 | NI | NI | C | 3.8 |  |
| B010 | 205 | 38 | 15.4 | 4.7 | NI | NI | C | 2.7 |  |
| B011 | 186 | 45 | 14.9 | 4.1 | NI | NI | C | 5.8 |  |
| B012 | 189 | 54 | 14.8 | 4.8 | NI | NI | A | 4.1 |  |
| B013 | 134 | 10 | 12.3 | 4.2 | NI | NI | C | 7 |  |
| B014 | 184 | 10 | 13.1 | 4.6 | NI | NI | B | 3.4 |  |
| B015 | 150 | 16 | 15.2 | 4.5 | NI | NI | C | 4.2 |  |
| B016 | 111 | 13 | 9.2 | 4.2 | NI | NI | NI | 3.5 |  |
| B017 | 157 | 19 | 12.4 | 4.3 | NI | NI | C | 4 |  |
| B018 | 185 | 11 | 11.8 | 4.7 | NI | NI | C | 9.2 |  |
| B019 | 163 | 15 | 11.5 | 4.6 | NI | NI | C | 3.7 |  |
| B020 | 351 | 13 | 12.9 | 4.6 | NI | NI | C | 3.7 |  |
|  |  |  |  |  |  |  |  |  |  |
| **Code No.** | **ALP** | **γGTP** | **HB** | **ALB** |  |  |  |  |  |
| NASH40 | 205 | 168 | 12.7 | 4.2 |  |  |  |  |  |
| NASH41 | 261 | 295 | 13.8 | 3.5 |  |  |  |  |  |
| NASH42 | 371 | 32 | 11 | 3.5 |  |  |  |  |  |
| NASH43 | 253 | 75 | 14.3 | 4 |  |  |  |  |  |
| NASH44 | 256 | 107 | 15.8 | 3.5 |  |  |  |  |  |
| NASH45 | 485 | 167 | 13.8 | 4.4 |  |  |  |  |  |
| NASH46 | 205 | 121 | 14.4 | 3.8 |  |  |  |  |  |
| NASH47 | 193 | 76 | 13 | 3.9 |  |  |  |  |  |

Abbreviations. CHC, chronic hepatitis C; CHB, chronic hepatitis B; NASH, non alcoholic steatohepatitis, NL, normal liver (healthy control); AST, aspartate aminotransferase; ALT, alanine aminotransferase; WBC, white blood cell; ALP, alkaline phosphatase; γGTP, gamma-glutamyltranspeptidase; inflammation, grade of inflammation estimated by Metavir score; fibrosis, stage of liver fibrosis estimated by Metavir score; outcome, outcome of the peginterferon and ribavirin combination treatment; SVR, sustained virological response; relapse; NR, non responder; NI, no information; NP not performed pegylatedinterferon and ribavirin combination therapy
