# Supplementary material for: Comprehensive miRNA Expression Analysis in Peripheral Blood Can Diagnose Liver Disease
Source: PLoS One. 2012 Oct 31;7(10):e48366. doi: 10.1371/journal.pone.0048366 (PMC3485241; doi:10.1371/journal.pone.0048366)
Supplement: Table S6 — Accuracy of LDA for “in silico” resampling. (DOCX) [file pone.0048366.s020.docx]

Table S6 Accuracy of LDA for “in silico” resampling

| **miRNA** | **miRNA_Probe** | **accuracy** | **miRNA** | **miRNA_Probe** | **accuracy** |
| --- | --- | --- | --- | --- | --- |
| miR-154* | A_25_P00010115 | 0.512921348 | miR-523 | A_25_P00010566 | 0.545505618 |
| miR-154* | A_25_P00010116 | 0.544382022 | miR-608 | A_25_P00010265 | 0.568539326 |
| miR-30b | A_25_P00010390 | 0.539325843 | miR-608 | A_25_P00010264 | 0.557865169 |
| miR-30b | A_25_P00010391 | 0.573033708 | miR-203 | A_25_P00010628 | 0.534269663 |
| miR-379 | A_25_P00010956 | 0.537640449 | miR-203 | A_25_P00010629 | 0.553370787 |
| miR-379 | A_25_P00010957 | 0.554494382 | miR-532 | A_25_P00010270 | 0.534269663 |
| miR-517b | A_25_P00011941 | 0.545505618 | miR-532 | A_25_P00010271 | 0.562921348 |
| miR-517b | A_25_P00011215 | 0.562921348 | miR-644 | A_25_P00011404 | 0.548876404 |
| miR-634 | A_25_P00010912 | 0.582022472 | miR-644 | A_25_P00011403 | 0.561235955 |
| miR-634 | A_25_P00010911 | 0.54494382 | miR-644 | A_25_P00011405 | 0.585955056 |
| miR-634 | A_25_P00010910 | 0.541011236 | miR-644 | A_25_P00011624 | 0.538764045 |
| miR-634 | A_25_P00010913 | 0.551123596 | miR-211 | A_25_P00010406 | 0.561235955 |
| miR-539 | A_25_P00010147 | 0.550561798 | miR-211 | A_25_P00010405 | 0.543258427 |
| miR-539 | A_25_P00010149 | 0.525842697 | miR-302c | A_25_P00010537 | 0.526404494 |
| miR-539 | A_25_P00010150 | 0.53988764 | miR-302c | A_25_P00010538 | 0.540449438 |
| miR-539 | A_25_P00010148 | 0.553932584 | miR-302c | A_25_P00010536 | 0.556179775 |
| miR-369-3p | A_25_P00010023 | 0.535393258 | miR-219 | A_25_P00010343 | 0.537078652 |
| miR-662 | A_25_P00010666 | 0.53988764 | miR-219 | A_25_P00010342 | 0.538764045 |
| miR-662 | A_25_P00010667 | 0.547191011 | miR-200b | A_25_P00010909 | 0.563483146 |
| miR-662 | A_25_P00010665 | 0.555617978 | miR-200b | A_25_P00010908 | 0.556741573 |
| miR-662 | A_25_P00010668 | 0.520224719 | miR-512-3p | A_25_P00010709 | 0.537078652 |
| miR-577 | A_25_P00010514 | 0.569662921 | miR-512-3p | A_25_P00010710 | 0.548314607 |
| miR-454-3p | A_25_P00011223 | 0.56011236 | miR-149 | A_25_P00010849 | 0.551123596 |
| miR-454-3p | A_25_P00010009 | 0.562359551 | miR-149 | A_25_P00010850 | 0.557865169 |
| miR-454-3p | A_25_P00011940 | 0.56741573 | miR-221 | A_25_P00010690 | 0.560674157 |
| miR-454-3p | A_25_P00010008 | 0.541011236 | miR-221 | A_25_P00010689 | 0.551123596 |
| miR-490 | A_25_P00010508 | 0.543820225 | miR-632 | A_25_P00011019 | 0.539325843 |
| miR-490 | A_25_P00010507 | 0.530898876 | miR-632 | A_25_P00011020 | 0.573595506 |
| miR-519e* | A_25_P00010697 | 0.580898876 | miR-632 | A_25_P00011021 | 0.563483146 |
| miR-519e* | A_25_P00010695 | 0.550561798 | miR-7 | A_25_P00010458 | 0.520786517 |
| miR-519e* | A_25_P00010696 | 0.533707865 | miR-7 | A_25_P00010457 | 0.541573034 |
| miR-32 | A_25_P00010752 | 0.556741573 | miR-508 | A_25_P00011040 | 0.556179775 |
| miR-651 | A_25_P00011329 | 0.530337079 | miR-508 | A_25_P00011041 | 0.536516854 |
| miR-651 | A_25_P00011330 | 0.549438202 | miR-508 | A_25_P00011039 | 0.547191011 |
| miR-651 | A_25_P00011606 | 0.523033708 | miR-425-3p | A_25_P00010860 | 0.551123596 |
| miR-651 | A_25_P00011327 | 0.564044944 | miR-425-3p | A_25_P00010861 | 0.553370787 |
| miR-125b | A_25_P00010980 | 0.557303371 | miR-425-3p | A_25_P00010862 | 0.556741573 |
| miR-125b | A_25_P00010979 | 0.534831461 | miR-27b | A_25_P00010762 | 0.56011236 |
| miR-545 | A_25_P00010477 | 0.515730337 | miR-27b | A_25_P00010761 | 0.565730337 |
| miR-545 | A_25_P00010476 | 0.558426966 | miR-592 | A_25_P00010207 | 0.547752809 |
| miR-545 | A_25_P00010479 | 0.539325843 | miR-592 | A_25_P00010206 | 0.529213483 |
| miR-545 | A_25_P00010478 | 0.565730337 | miR-769-5p | A_25_P00011955 | 0.53258427 |
| miR-30e-3p | A_25_P00010838 | 0.534269663 | miR-769-5p | A_25_P00011305 | 0.552808989 |
| miR-30e-3p | A_25_P00010837 | 0.564606742 | miR-769-5p | A_25_P00011954 | 0.558988764 |
| miR-182* | A_25_P00010967 | 0.551685393 | miR-769-5p | A_25_P00011306 | 0.564044944 |
| miR-182* | A_25_P00010968 | 0.547752809 | miR-581 | A_25_P00011284 | 0.543820225 |
| miR-182* | A_25_P00010965 | 0.531460674 | miR-581 | A_25_P00011592 | 0.545505618 |
| miR-182* | A_25_P00010966 | 0.536516854 | miR-581 | A_25_P00011285 | 0.541573034 |
| miR-26b | A_25_P00010084 | 0.550561798 | miR-581 | A_25_P00011283 | 0.541011236 |
| miR-26b | A_25_P00010085 | 0.546067416 | miR-601 | A_25_P00010641 | 0.513483146 |
| miR-326 | A_25_P00010670 | 0.549438202 | miR-601 | A_25_P00010640 | 0.523595506 |
| miR-326 | A_25_P00010671 | 0.571910112 | miR-601 | A_25_P00010642 | 0.551123596 |
| miR-326 | A_25_P00010669 | 0.544382022 | miR-601 | A_25_P00010643 | 0.537078652 |
| miR-335 | A_25_P00010213 | 0.533146067 | miR-520d* | A_25_P00010494 | 0.569662921 |
| miR-335 | A_25_P00010214 | 0.54494382 | miR-520d* | A_25_P00010495 | 0.552247191 |
| miR-335 | A_25_P00010212 | 0.552808989 | miR-654 | A_25_P00011961 | 0.564606742 |
| miR-135b | A_25_P00010486 | 0.554494382 | miR-654 | A_25_P00011392 | 0.547191011 |
| miR-586 | A_25_P00011626 | 0.533707865 | miR-654 | A_25_P00011960 | 0.550561798 |
| miR-586 | A_25_P00011418 | 0.546629213 | miR-654 | A_25_P00010760 | 0.556179775 |
| miR-586 | A_25_P00011420 | 0.537078652 | miR-200a* | A_25_P00011011 | 0.538202247 |
| miR-586 | A_25_P00011419 | 0.557865169 | miR-200a* | A_25_P00011012 | 0.511797753 |
| miR-337 | A_25_P00010029 | 0.570786517 | miR-200a* | A_25_P00011010 | 0.540449438 |
| miR-337 | A_25_P00010030 | 0.546629213 | miR-643 | A_25_P00011363 | 0.551685393 |
| miR-337 | A_25_P00010032 | 0.562359551 | miR-643 | A_25_P00011362 | 0.556179775 |
| miR-337 | A_25_P00010031 | 0.553932584 | miR-643 | A_25_P00011616 | 0.573595506 |
| miR-496 | A_25_P00010721 | 0.530898876 | miR-643 | A_25_P00011361 | 0.543258427 |
| miR-496 | A_25_P00010720 | 0.526966292 | miR-154 | A_25_P00010584 | 0.538202247 |
| miR-125a | A_25_P00010481 | 0.47752809 | miR-154 | A_25_P00010583 | 0.556179775 |
| miR-125a | A_25_P00010480 | 0.521910112 | miR-668 | A_25_P00011971 | 0.563483146 |
| miR-193a | A_25_P00010674 | 0.535393258 | miR-668 | A_25_P00011970 | 0.544382022 |
| miR-193a | A_25_P00010675 | 0.606741573 | miR-668 | A_25_P00011432 | 0.526966292 |
| miR-191 | A_25_P00010392 | 0.55505618 | miR-668 | A_25_P00011433 | 0.561797753 |
| miR-191 | A_25_P00010393 | 0.594382022 | miR-10b | A_25_P00010136 | 0.525842697 |
| miR-191 | A_25_P00010394 | 0.583146067 | miR-10b | A_25_P00010135 | 0.575280899 |
| miR-659 | A_25_P00011100 | 0.55505618 | miR-210 | A_25_P00010995 | 0.593820225 |
| miR-659 | A_25_P00011099 | 0.578651685 | miR-210 | A_25_P00010996 | 0.587078652 |
| miR-659 | A_25_P00011098 | 0.516853933 | miR-620 | A_25_P00010450 | 0.561235955 |
| miR-574 | A_25_P00010930 | 0.517977528 | miR-217 | A_25_P00010425 | 0.529213483 |
| miR-574 | A_25_P00010932 | 0.561235955 | miR-217 | A_25_P00010424 | 0.565730337 |
| miR-574 | A_25_P00010931 | 0.557865169 | miR-217 | A_25_P00010423 | 0.54494382 |
| miR-20a | A_25_P00010613 | 0.528651685 | miR-217 | A_25_P00010426 | 0.533707865 |
| miR-20a | A_25_P00010612 | 0.538764045 | miR-544 | A_25_P00010312 | 0.563483146 |
| miR-30e-5p | A_25_P00011013 | 0.557303371 | miR-544 | A_25_P00010309 | 0.542696629 |
| miR-30e-5p | A_25_P00011014 | 0.576966292 | miR-544 | A_25_P00010310 | 0.530898876 |
| miR-30e-5p | A_25_P00011015 | 0.598314607 | miR-544 | A_25_P00010311 | 0.566853933 |
| miR-422b | A_25_P00010858 | 0.528089888 | miR-616 | A_25_P00011240 | 0.547752809 |
| miR-422b | A_25_P00010859 | 0.508426966 | miR-616 | A_25_P00011241 | 0.535955056 |
| miR-377 | A_25_P00010125 | 0.596629213 | miR-616 | A_25_P00011585 | 0.556741573 |
| miR-377 | A_25_P00010126 | 0.545505618 | miR-616 | A_25_P00011242 | 0.554494382 |
| miR-432* | A_25_P00010356 | 0.532022472 | miR-320 | A_25_P00011116 | 0.534831461 |
| miR-432* | A_25_P00010355 | 0.566292135 | miR-320 | A_25_P00011115 | 0.565730337 |
| miR-432* | A_25_P00010354 | 0.538764045 | miR-299-5p | A_25_P00010445 | 0.552808989 |
| miR-432* | A_25_P00010357 | 0.550561798 | miR-299-5p | A_25_P00010446 | 0.564044944 |
| miR-150 | A_25_P00010490 | 0.537078652 | miR-631 | A_25_P00010826 | 0.534269663 |
| miR-150 | A_25_P00010489 | 0.56741573 | miR-631 | A_25_P00010825 | 0.551685393 |
| miR-450 | A_25_P00010972 | 0.50505618 | miR-200c | A_25_P00010546 | 0.532022472 |
| miR-340 | A_25_P00010867 | 0.595505618 | miR-200c | A_25_P00010545 | 0.571910112 |
| miR-340 | A_25_P00010866 | 0.55 | miR-519e | A_25_P00010165 | 0.548314607 |
| miR-433 | A_25_P00010158 | 0.531460674 | miR-519e | A_25_P00010166 | 0.55 |
| miR-433 | A_25_P00010159 | 0.541011236 | miR-380-5p | A_25_P00010790 | 0.550561798 |
| miR-296 | A_25_P00010197 | 0.515730337 | miR-380-5p | A_25_P00010792 | 0.563483146 |
| miR-296 | A_25_P00010196 | 0.524157303 | miR-380-5p | A_25_P00010793 | 0.553932584 |
| miR-604 | A_25_P00010916 | 0.548876404 | miR-380-5p | A_25_P00010791 | 0.576966292 |
| miR-604 | A_25_P00010915 | 0.530337079 | miR-548a | A_25_P00010747 | 0.519662921 |
| miR-604 | A_25_P00010914 | 0.524157303 | miR-548a | A_25_P00010748 | 0.547752809 |
| miR-767-5p | A_25_P00011348 | 0.530337079 | miR-548a | A_25_P00010746 | 0.537640449 |
| miR-767-5p | A_25_P00011347 | 0.534831461 | miR-548a | A_25_P00010749 | 0.56011236 |
| miR-767-5p | A_25_P00011345 | 0.530898876 | miR-122a | A_25_P00010128 | 0.565168539 |
| miR-767-5p | A_25_P00011346 | 0.533707865 | miR-122a | A_25_P00010127 | 0.562359551 |
| miR-624 | A_25_P00011055 | 0.571348315 | miR-584 | A_25_P00010635 | 0.564606742 |
| miR-624 | A_25_P00011054 | 0.557303371 | miR-584 | A_25_P00010634 | 0.53988764 |
| miR-624 | A_25_P00011056 | 0.540449438 | miR-584 | A_25_P00010636 | 0.534831461 |
| miR-624 | A_25_P00011053 | 0.561235955 | miR-521 | A_25_P00010172 | 0.546629213 |
| miR-622 | A_25_P00010807 | 0.478651685 | miR-521 | A_25_P00010173 | 0.543258427 |
| miR-622 | A_25_P00010806 | 0.547191011 | miR-619 | A_25_P00011293 | 0.517977528 |
| miR-622 | A_25_P00010805 | 0.529213483 | miR-619 | A_25_P00011291 | 0.538202247 |
| miR-633 | A_25_P00011123 | 0.552808989 | miR-619 | A_25_P00011290 | 0.548314607 |
| miR-633 | A_25_P00011641 | 0.552247191 | miR-619 | A_25_P00011594 | 0.566292135 |
| miR-633 | A_25_P00011124 | 0.550561798 | miR-188 | A_25_P00010230 | 0.549438202 |
| miR-633 | A_25_P00011640 | 0.581460674 | miR-188 | A_25_P00010231 | 0.566292135 |
| miR-609 | A_25_P00010362 | 0.549438202 | miR-188 | A_25_P00010232 | 0.550561798 |
| miR-609 | A_25_P00011597 | 0.533146067 | miR-23a | A_25_P00010844 | 0.575280899 |
| miR-609 | A_25_P00011304 | 0.538202247 | miR-23a | A_25_P00010843 | 0.55 |
| miR-609 | A_25_P00011303 | 0.546067416 | miR-34a | A_25_P00010046 | 0.537640449 |
| miR-23b | A_25_P00010881 | 0.557303371 | miR-34a | A_25_P00010045 | 0.536516854 |
| miR-23b | A_25_P00010882 | 0.555617978 | miR-363 | A_25_P00010953 | 0.521910112 |
| miR-452* | A_25_P00010686 | 0.552808989 | miR-363 | A_25_P00010954 | 0.552808989 |
| miR-452* | A_25_P00010684 | 0.555617978 | miR-548d | A_25_P00010737 | 0.53988764 |
| miR-452* | A_25_P00010685 | 0.538764045 | miR-548d | A_25_P00010736 | 0.536516854 |
| miR-519d | A_25_P00010174 | 0.557865169 | miR-548d | A_25_P00010739 | 0.547191011 |
| miR-519d | A_25_P00010175 | 0.534831461 | miR-548d | A_25_P00010738 | 0.543820225 |
| miR-202* | A_25_P00010016 | 0.571348315 | miR-500 | A_25_P00010502 | 0.537640449 |
| miR-605 | A_25_P00010397 | 0.570224719 | miR-500 | A_25_P00010501 | 0.552247191 |
| miR-605 | A_25_P00010398 | 0.547752809 | miR-451 | A_25_P00010014 | 0.54494382 |
| miR-516-3p | A_25_P00010233 | 0.539325843 | miR-451 | A_25_P00010015 | 0.507303371 |
| miR-516-3p | A_25_P00010235 | 0.519662921 | miR-449 | A_25_P00010220 | 0.57752809 |
| miR-516-3p | A_25_P00010234 | 0.561235955 | miR-449 | A_25_P00010222 | 0.55 |
| miR-518f | A_25_P00011028 | 0.552808989 | miR-449 | A_25_P00010221 | 0.517977528 |
| miR-518f | A_25_P00011029 | 0.536516854 | miR-449 | A_25_P00010219 | 0.543258427 |
| miR-155 | A_25_P00010553 | 0.524157303 | miR-589 | A_25_P00011052 | 0.549438202 |
| miR-155 | A_25_P00010555 | 0.56741573 | miR-589 | A_25_P00011051 | 0.552247191 |
| miR-155 | A_25_P00010554 | 0.569101124 | miR-589 | A_25_P00011050 | 0.550561798 |
| miR-556 | A_25_P00011340 | 0.515730337 | miR-589 | A_25_P00011049 | 0.534831461 |
| miR-556 | A_25_P00010518 | 0.54494382 | miR-525 | A_25_P00010330 | 0.53988764 |
| miR-556 | A_25_P00011339 | 0.524719101 | miR-525 | A_25_P00010331 | 0.535955056 |
| miR-556 | A_25_P00010517 | 0.561235955 | miR-636 | A_25_P00010663 | 0.602247191 |
| miR-492 | A_25_P00010094 | 0.538764045 | miR-636 | A_25_P00010661 | 0.558988764 |
| miR-492 | A_25_P00010093 | 0.552808989 | miR-636 | A_25_P00010664 | 0.537640449 |
| miR-493-5p | A_25_P00010117 | 0.575280899 | miR-636 | A_25_P00010662 | 0.556741573 |
| miR-493-5p | A_25_P00010119 | 0.532022472 | miR-153 | A_25_P00010988 | 0.551123596 |
| miR-493-5p | A_25_P00010118 | 0.533707865 | miR-189 | A_25_P00010296 | 0.528089888 |
| miR-493-5p | A_25_P00010120 | 0.578651685 | miR-189 | A_25_P00010295 | 0.558426966 |
| miR-526b* | A_25_P00010784 | 0.566292135 | miR-486 | A_25_P00010644 | 0.55 |
| miR-526b* | A_25_P00010783 | 0.533707865 | miR-486 | A_25_P00010645 | 0.573033708 |
| miR-801 | A_25_P00011440 | 0.495505618 | miR-617 | A_25_P00010772 | 0.553932584 |
| miR-801 | A_25_P00011974 | 0.547191011 | miR-617 | A_25_P00010773 | 0.541573034 |
| miR-801 | A_25_P00011441 | 0.533146067 | miR-617 | A_25_P00010771 | 0.556179775 |
| miR-801 | A_25_P00011975 | 0.48988764 | miR-372 | A_25_P00010259 | 0.547752809 |
| miR-517a | A_25_P00010052 | 0.541011236 | miR-372 | A_25_P00010258 | 0.53988764 |
| miR-517a | A_25_P00010051 | 0.533707865 | miR-95 | A_25_P00011085 | 0.571910112 |
| miR-599 | A_25_P00010948 | 0.548876404 | miR-95 | A_25_P00011086 | 0.529775281 |
| miR-599 | A_25_P00011636 | 0.540449438 | miR-186 | A_25_P00010101 | 0.547752809 |
| miR-599 | A_25_P00011462 | 0.53988764 | miR-186 | A_25_P00010102 | 0.564606742 |
| miR-599 | A_25_P00011461 | 0.570786517 | miR-106b | A_25_P00010433 | 0.534831461 |
| miR-192 | A_25_P00010869 | 0.564044944 | miR-106b | A_25_P00010434 | 0.563483146 |
| miR-192 | A_25_P00010868 | 0.602247191 | miR-512-5p | A_25_P00010693 | 0.571910112 |
| miR-148a | A_25_P00010131 | 0.598876404 | miR-512-5p | A_25_P00010694 | 0.517977528 |
| miR-148a | A_25_P00010132 | 0.538202247 | miR-302b* | A_25_P00010324 | 0.533707865 |
| miR-362 | A_25_P00010284 | 0.538764045 | miR-302b* | A_25_P00010321 | 0.552247191 |
| miR-362 | A_25_P00010283 | 0.516292135 | miR-302b* | A_25_P00010322 | 0.548314607 |
| miR-484 | A_25_P00010431 | 0.557303371 | miR-302b* | A_25_P00010323 | 0.526966292 |
| miR-484 | A_25_P00010432 | 0.553370787 | miR-638 | A_25_P00010403 | 0.590449438 |
| miR-607 | A_25_P00010935 | 0.512359551 | miR-638 | A_25_P00010402 | 0.529775281 |
| miR-607 | A_25_P00011448 | 0.538764045 | miR-638 | A_25_P00010404 | 0.575842697 |
| miR-607 | A_25_P00010936 | 0.541011236 | miR-638 | A_25_P00010401 | 0.526404494 |
| miR-607 | A_25_P00011449 | 0.558426966 | miR-515-3p | A_25_P00010260 | 0.574157303 |
| miR-21 | A_25_P00010975 | 0.569101124 | miR-515-3p | A_25_P00010261 | 0.53988764 |
| miR-21 | A_25_P00010976 | 0.576966292 | miR-645 | A_25_P00010991 | 0.574157303 |
| miR-134 | A_25_P00010570 | 0.548876404 | miR-645 | A_25_P00010992 | 0.543258427 |
| miR-134 | A_25_P00010569 | 0.552247191 | miR-645 | A_25_P00010994 | 0.547191011 |
| miR-138 | A_25_P00010733 | 0.55505618 | miR-645 | A_25_P00010993 | 0.518539326 |
| miR-376b | A_25_P00010061 | 0.561797753 | miR-625 | A_25_P00010396 | 0.550561798 |
| miR-376b | A_25_P00010063 | 0.561797753 | miR-625 | A_25_P00010395 | 0.56011236 |
| miR-376b | A_25_P00010062 | 0.53988764 | miR-139 | A_25_P00010349 | 0.592696629 |
| miR-495 | A_25_P00010017 | 0.569101124 | miR-139 | A_25_P00010350 | 0.569662921 |
| miR-495 | A_25_P00010018 | 0.565168539 | miR-342 | A_25_P00010712 | 0.552247191 |
| miR-103 | A_25_P00011004 | 0.525280899 | miR-342 | A_25_P00010711 | 0.557865169 |
| miR-103 | A_25_P00011005 | 0.566292135 | miR-220 | A_25_P00010039 | 0.575280899 |
| miR-519b | A_25_P00010011 | 0.571910112 | miR-220 | A_25_P00010037 | 0.552247191 |
| miR-519b | A_25_P00010010 | 0.569101124 | miR-220 | A_25_P00010040 | 0.540449438 |
| miR-199b | A_25_P00010698 | 0.588202247 | miR-220 | A_25_P00010038 | 0.534831461 |
| miR-199b | A_25_P00010699 | 0.557865169 | miR-520h | A_25_P00010294 | 0.570224719 |
| miR-137 | A_25_P00010617 | 0.553370787 | miR-520h | A_25_P00010293 | 0.554494382 |
| miR-137 | A_25_P00010616 | 0.54494382 | miR-520a* | A_25_P00010329 | 0.569662921 |
| miR-302a | A_25_P00010982 | 0.541011236 | miR-520a* | A_25_P00010328 | 0.492134831 |
| miR-302a | A_25_P00010983 | 0.548314607 | hsa-let-7b | A_25_P00010070 | 0.535955056 |
| miR-526c | A_25_P00010057 | 0.551685393 | hsa-let-7b | A_25_P00010071 | 0.526966292 |
| miR-526c | A_25_P00010056 | 0.532022472 | miR-555 | A_25_P00011320 | 0.552808989 |
| miR-526c | A_25_P00010055 | 0.530337079 | miR-555 | A_25_P00011319 | 0.521348315 |
| miR-30d | A_25_P00010683 | 0.597191011 | miR-555 | A_25_P00011601 | 0.545505618 |
| miR-30d | A_25_P00010682 | 0.561235955 | miR-555 | A_25_P00011318 | 0.53988764 |
| miR-181a* | A_25_P00010884 | 0.556179775 | miR-629 | A_25_P00010833 | 0.533146067 |
| miR-181a* | A_25_P00010886 | 0.545505618 | miR-629 | A_25_P00010834 | 0.542696629 |
| miR-181a* | A_25_P00010883 | 0.566292135 | miR-629 | A_25_P00010831 | 0.555617978 |
| miR-181a* | A_25_P00010885 | 0.561235955 | miR-629 | A_25_P00010832 | 0.547752809 |
| miR-411 | A_25_P00010604 | 0.556741573 | miR-324-3p | A_25_P00010540 | 0.546629213 |
| miR-411 | A_25_P00010605 | 0.563483146 | miR-324-3p | A_25_P00010539 | 0.547191011 |
| miR-302c* | A_25_P00010506 | 0.56011236 | miR-431 | A_25_P00011084 | 0.543820225 |
| miR-302c* | A_25_P00010505 | 0.547191011 | miR-431 | A_25_P00011083 | 0.526966292 |
| miR-9* | A_25_P00010315 | 0.55 | miR-558 | A_25_P00010452 | 0.536516854 |
| miR-9* | A_25_P00010318 | 0.525280899 | miR-558 | A_25_P00011605 | 0.539325843 |
| miR-9* | A_25_P00010317 | 0.536516854 | miR-558 | A_25_P00011326 | 0.551123596 |
| miR-9* | A_25_P00010316 | 0.561797753 | miR-558 | A_25_P00011325 | 0.556741573 |
| miR-518d | A_25_P00010803 | 0.50505618 | miR-573 | A_25_P00011367 | 0.542134831 |
| miR-518d | A_25_P00010804 | 0.551123596 | miR-573 | A_25_P00011366 | 0.54494382 |
| miR-1 | A_25_P00010955 | 0.596629213 | miR-573 | A_25_P00011364 | 0.550561798 |
| miR-675 | A_25_P00011608 | 0.586516854 | miR-573 | A_25_P00011618 | 0.548876404 |
| miR-675 | A_25_P00011611 | 0.549438202 | miR-16 | A_25_P00010599 | 0.553932584 |
| miR-675 | A_25_P00011609 | 0.57247191 | miR-16 | A_25_P00010598 | 0.570786517 |
| miR-675 | A_25_P00011610 | 0.532022472 | miR-570 | A_25_P00011463 | 0.52247191 |
| miR-141 | A_25_P00010548 | 0.551685393 | miR-570 | A_25_P00011466 | 0.538202247 |
| miR-141 | A_25_P00010547 | 0.573033708 | miR-570 | A_25_P00011464 | 0.524719101 |
| miR-671 | A_25_P00011270 | 0.576966292 | miR-570 | A_25_P00011637 | 0.545505618 |
| miR-671 | A_25_P00011269 | 0.561797753 | miR-765 | A_25_P00010520 | 0.541011236 |
| miR-671 | A_25_P00011590 | 0.529213483 | miR-765 | A_25_P00011959 | 0.549438202 |
| miR-671 | A_25_P00011267 | 0.526404494 | miR-765 | A_25_P00011342 | 0.557865169 |
| miR-367 | A_25_P00010985 | 0.57752809 | miR-765 | A_25_P00011341 | 0.535393258 |
| miR-367 | A_25_P00010984 | 0.538764045 | miR-612 | A_25_P00011238 | 0.529775281 |
| miR-22 | A_25_P00010204 | 0.568539326 | miR-612 | A_25_P00011239 | 0.544382022 |
| miR-22 | A_25_P00010205 | 0.582022472 | miR-612 | A_25_P00011237 | 0.546067416 |
| hsa-let-7c | A_25_P00010073 | 0.546067416 | miR-612 | A_25_P00011236 | 0.528089888 |
| hsa-let-7c | A_25_P00010072 | 0.538202247 | miR-15a | A_25_P00010467 | 0.556179775 |
| miR-381 | A_25_P00010183 | 0.536516854 | miR-15a | A_25_P00010468 | 0.546067416 |
| miR-381 | A_25_P00010182 | 0.545505618 | miR-20b | A_25_P00010615 | 0.576966292 |
| miR-582 | A_25_P00010411 | 0.537078652 | miR-20b | A_25_P00010614 | 0.579775281 |
| miR-582 | A_25_P00010412 | 0.526966292 | miR-663 | A_25_P00010799 | 0.515168539 |
| miR-602 | A_25_P00010872 | 0.548314607 | miR-663 | A_25_P00010798 | 0.558426966 |
| miR-602 | A_25_P00010874 | 0.546629213 | miR-663 | A_25_P00010800 | 0.558988764 |
| miR-602 | A_25_P00010873 | 0.592696629 | miR-331 | A_25_P00011117 | 0.574157303 |
| miR-378 | A_25_P00010152 | 0.537078652 | miR-331 | A_25_P00011118 | 0.603932584 |
| miR-378 | A_25_P00010151 | 0.578089888 | miR-628 | A_25_P00010593 | 0.53988764 |
| miR-768-5p | A_25_P00011956 | 0.520786517 | miR-628 | A_25_P00010592 | 0.538202247 |
| miR-768-5p | A_25_P00011314 | 0.509550562 | miR-628 | A_25_P00010591 | 0.541011236 |
| miR-768-5p | A_25_P00011315 | 0.52752809 | miR-628 | A_25_P00010590 | 0.565730337 |
| miR-768-5p | A_25_P00011957 | 0.542134831 | miR-182 | A_25_P00011104 | 0.538764045 |
| miR-509 | A_25_P00011035 | 0.544382022 | miR-182 | A_25_P00011103 | 0.56011236 |
| miR-509 | A_25_P00011034 | 0.512359551 | miR-216 | A_25_P00010482 | 0.555617978 |
| miR-648 | A_25_P00010223 | 0.533146067 | miR-216 | A_25_P00010484 | 0.523033708 |
| miR-648 | A_25_P00010224 | 0.53258427 | miR-216 | A_25_P00010485 | 0.552247191 |
| miR-648 | A_25_P00010225 | 0.567977528 | miR-216 | A_25_P00010483 | 0.547191011 |
| miR-614 | A_25_P00010535 | 0.53258427 | miR-133a | A_25_P00010191 | 0.556179775 |
| miR-614 | A_25_P00010534 | 0.516292135 | miR-133a | A_25_P00010190 | 0.567977528 |
| miR-614 | A_25_P00010533 | 0.547752809 | miR-571 | A_25_P00010632 | 0.51741573 |
| miR-9 | A_25_P00011001 | 0.557303371 | miR-571 | A_25_P00010633 | 0.541011236 |
| miR-9 | A_25_P00011002 | 0.540449438 | miR-424 | A_25_P00011109 | 0.543820225 |
| miR-9 | A_25_P00011003 | 0.540449438 | miR-424 | A_25_P00011110 | 0.541573034 |
| miR-519c | A_25_P00010428 | 0.538764045 | miR-767-3p | A_25_P00011300 | 0.542696629 |
| miR-519c | A_25_P00010427 | 0.561797753 | miR-767-3p | A_25_P00011302 | 0.561797753 |
| miR-652 | A_25_P00011977 | 0.546067416 | miR-767-3p | A_25_P00011299 | 0.56011236 |
| miR-652 | A_25_P00011467 | 0.553932584 | miR-767-3p | A_25_P00011301 | 0.536516854 |
| miR-652 | A_25_P00011082 | 0.533707865 | miR-302a* | A_25_P00010024 | 0.583146067 |
| miR-652 | A_25_P00011976 | 0.56011236 | miR-302a* | A_25_P00010025 | 0.533707865 |
| miR-24 | A_25_P00010676 | 0.562359551 | miR-302a* | A_25_P00010026 | 0.538202247 |
| miR-24 | A_25_P00010677 | 0.542696629 | miR-224 | A_25_P00010949 | 0.565730337 |
| miR-380-3p | A_25_P00010100 | 0.557865169 | miR-224 | A_25_P00010950 | 0.532022472 |
| miR-380-3p | A_25_P00010099 | 0.561797753 | miR-193b | A_25_P00010027 | 0.575842697 |
| miR-363* | A_25_P00010042 | 0.535955056 | miR-193b | A_25_P00010028 | 0.569101124 |
| miR-363* | A_25_P00010041 | 0.542134831 | miR-105 | A_25_P00010193 | 0.538202247 |
| miR-363* | A_25_P00010044 | 0.542134831 | miR-105 | A_25_P00010194 | 0.543258427 |
| miR-363* | A_25_P00010043 | 0.519101124 | miR-105 | A_25_P00010192 | 0.570786517 |
| miR-520d | A_25_P00010075 | 0.542134831 | miR-105 | A_25_P00010195 | 0.548314607 |
| miR-520d | A_25_P00010074 | 0.537078652 | miR-606 | A_25_P00011603 | 0.547191011 |
| miR-507 | A_25_P00011111 | 0.564606742 | miR-606 | A_25_P00011604 | 0.571348315 |
| miR-507 | A_25_P00011114 | 0.547191011 | miR-606 | A_25_P00010435 | 0.576966292 |
| miR-507 | A_25_P00011112 | 0.554494382 | miR-606 | A_25_P00010436 | 0.524157303 |
| miR-507 | A_25_P00011113 | 0.561235955 | miR-517* | A_25_P00010352 | 0.565168539 |
| miR-199a* | A_25_P00010069 | 0.536516854 | miR-517* | A_25_P00010351 | 0.557303371 |
| miR-199a* | A_25_P00010068 | 0.533707865 | miR-517* | A_25_P00010353 | 0.561797753 |
| miR-365 | A_25_P00010416 | 0.54494382 | miR-561 | A_25_P00011294 | 0.547752809 |
| miR-365 | A_25_P00010415 | 0.550561798 | miR-561 | A_25_P00011595 | 0.547191011 |
| miR-30c | A_25_P00010815 | 0.528651685 | miR-561 | A_25_P00011295 | 0.542134831 |
| miR-30c | A_25_P00010816 | 0.546629213 | miR-561 | A_25_P00011296 | 0.554494382 |
| miR-640 | A_25_P00010373 | 0.569101124 | miR-503 | A_25_P00010657 | 0.528089888 |
| miR-640 | A_25_P00010372 | 0.558426966 | miR-503 | A_25_P00010658 | 0.541011236 |
| miR-640 | A_25_P00010371 | 0.543820225 | hsa-let-7g | A_25_P00010305 | 0.557303371 |
| miR-202 | A_25_P00011128 | 0.566853933 | hsa-let-7g | A_25_P00010306 | 0.548314607 |
| miR-202 | A_25_P00011130 | 0.553932584 | miR-501 | A_25_P00011043 | 0.542696629 |
| miR-202 | A_25_P00011129 | 0.550561798 | miR-501 | A_25_P00011042 | 0.540449438 |
| miR-215 | A_25_P00010926 | 0.510674157 | miR-501 | A_25_P00011044 | 0.552247191 |
| miR-215 | A_25_P00010927 | 0.553370787 | miR-501 | A_25_P00011045 | 0.566853933 |
| miR-212 | A_25_P00010853 | 0.566292135 | miR-576 | A_25_P00011607 | 0.558988764 |
| miR-212 | A_25_P00010854 | 0.558988764 | miR-576 | A_25_P00011334 | 0.550561798 |
| miR-766 | A_25_P00011965 | 0.538202247 | miR-576 | A_25_P00011331 | 0.555617978 |
| miR-766 | A_25_P00011964 | 0.534269663 | miR-576 | A_25_P00011333 | 0.575280899 |
| miR-766 | A_25_P00011411 | 0.524719101 | miR-325 | A_25_P00010179 | 0.53988764 |
| miR-766 | A_25_P00011410 | 0.569662921 | miR-325 | A_25_P00010178 | 0.539325843 |
| hsa-let-7a | A_25_P00011584 | 0.561235955 | miR-325 | A_25_P00010180 | 0.552808989 |
| hsa-let-7a | A_25_P00010086 | 0.535955056 | miR-325 | A_25_P00010181 | 0.588764045 |
| miR-214 | A_25_P00010659 | 0.582022472 | miR-222 | A_25_P00010719 | 0.589325843 |
| miR-214 | A_25_P00010660 | 0.528651685 | miR-222 | A_25_P00010718 | 0.562921348 |
| miR-485-3p | A_25_P00010361 | 0.56741573 | miR-655 | A_25_P00011229 | 0.552247191 |
| miR-485-3p | A_25_P00010360 | 0.551685393 | miR-655 | A_25_P00010021 | 0.539325843 |
| miR-19b | A_25_P00011000 | 0.529213483 | miR-655 | A_25_P00011228 | 0.523033708 |
| miR-19b | A_25_P00010999 | 0.542134831 | miR-655 | A_25_P00011227 | 0.53988764 |
| miR-196a | A_25_P00010526 | 0.549438202 | miR-18a* | A_25_P00010338 | 0.534269663 |
| miR-196a | A_25_P00010525 | 0.537640449 | miR-18a* | A_25_P00010339 | 0.574157303 |
| miR-374 | A_25_P00010492 | 0.537078652 | miR-18a* | A_25_P00010340 | 0.541573034 |
| miR-374 | A_25_P00010493 | 0.592134831 | miR-18a* | A_25_P00010341 | 0.553370787 |
| miR-374 | A_25_P00010491 | 0.582022472 | miR-96 | A_25_P00010735 | 0.582022472 |
| miR-98 | A_25_P00010047 | 0.564606742 | miR-96 | A_25_P00010734 | 0.549438202 |
| miR-98 | A_25_P00010048 | 0.553932584 | miR-639 | A_25_P00011943 | 0.533146067 |
| miR-373* | A_25_P00010842 | 0.571348315 | miR-639 | A_25_P00011944 | 0.519662921 |
| miR-373* | A_25_P00010841 | 0.559550562 | miR-639 | A_25_P00011255 | 0.545505618 |
| miR-453 | A_25_P00010581 | 0.555617978 | miR-639 | A_25_P00011256 | 0.537640449 |
| miR-453 | A_25_P00010579 | 0.542696629 | miR-346 | A_25_P00010368 | 0.554494382 |
| miR-453 | A_25_P00010582 | 0.55 | miR-346 | A_25_P00010369 | 0.546067416 |
| miR-453 | A_25_P00010580 | 0.558426966 | miR-346 | A_25_P00010370 | 0.548876404 |
| miR-661 | A_25_P00011951 | 0.554494382 | miR-195 | A_25_P00010770 | 0.568539326 |
| miR-661 | A_25_P00011953 | 0.55 | miR-195 | A_25_P00010769 | 0.541011236 |
| miR-661 | A_25_P00011275 | 0.553932584 | miR-562 | A_25_P00011388 | 0.552808989 |
| miR-661 | A_25_P00011952 | 0.545505618 | miR-562 | A_25_P00011390 | 0.550561798 |
| miR-369-5p | A_25_P00010789 | 0.552808989 | miR-562 | A_25_P00011389 | 0.531460674 |
| miR-369-5p | A_25_P00010788 | 0.557303371 | miR-562 | A_25_P00011391 | 0.585955056 |
| miR-30a-5p | A_25_P00010681 | 0.567977528 | miR-147 | A_25_P00010763 | 0.534831461 |
| miR-30a-5p | A_25_P00010680 | 0.53988764 | miR-147 | A_25_P00010764 | 0.56011236 |
| miR-518c* | A_25_P00010498 | 0.574157303 | miR-127 | A_25_P00010382 | 0.558988764 |
| miR-518c* | A_25_P00010496 | 0.592696629 | miR-127 | A_25_P00010381 | 0.536516854 |
| miR-518c* | A_25_P00010497 | 0.541573034 | miR-497 | A_25_P00010130 | 0.58258427 |
| miR-329 | A_25_P00010019 | 0.569662921 | miR-497 | A_25_P00010129 | 0.542134831 |
| miR-329 | A_25_P00010020 | 0.53988764 | miR-641 | A_25_P00011383 | 0.530337079 |
| miR-205 | A_25_P00010504 | 0.548876404 | miR-641 | A_25_P00011621 | 0.566853933 |
| miR-205 | A_25_P00010503 | 0.557865169 | miR-641 | A_25_P00011381 | 0.57247191 |
| miR-146b | A_25_P00010387 | 0.541573034 | miR-641 | A_25_P00011380 | 0.556179775 |
| miR-146b | A_25_P00010386 | 0.547191011 | miR-569 | A_25_P00011596 | 0.593820225 |
| miR-588 | A_25_P00011456 | 0.560674157 | miR-569 | A_25_P00010313 | 0.566853933 |
| miR-588 | A_25_P00011454 | 0.546629213 | miR-569 | A_25_P00011297 | 0.596629213 |
| miR-588 | A_25_P00011634 | 0.558988764 | miR-569 | A_25_P00011298 | 0.565730337 |
| miR-588 | A_25_P00011455 | 0.570786517 | miR-181d | A_25_P00010077 | 0.52247191 |
| miR-518c | A_25_P00010488 | 0.546067416 | miR-181d | A_25_P00010076 | 0.538764045 |
| miR-518c | A_25_P00010487 | 0.54494382 | miR-345 | A_25_P00010778 | 0.541573034 |
| miR-132 | A_25_P00010588 | 0.551123596 | miR-345 | A_25_P00010777 | 0.535393258 |
| miR-132 | A_25_P00010589 | 0.519101124 | miR-610 | A_25_P00011023 | 0.561235955 |
| miR-557 | A_25_P00010344 | 0.563483146 | miR-610 | A_25_P00011022 | 0.562921348 |
| miR-557 | A_25_P00010345 | 0.550561798 | miR-610 | A_25_P00011025 | 0.552808989 |
| miR-502 | A_25_P00010959 | 0.567977528 | miR-610 | A_25_P00011024 | 0.542134831 |
| miR-502 | A_25_P00010958 | 0.537078652 | miR-184 | A_25_P00010236 | 0.528089888 |
| miR-376a | A_25_P00010274 | 0.571910112 | miR-184 | A_25_P00010237 | 0.537640449 |
| miR-376a | A_25_P00010276 | 0.553370787 | miR-494 | A_25_P00010098 | 0.463483146 |
| miR-376a | A_25_P00010275 | 0.566853933 | miR-494 | A_25_P00010097 | 0.532022472 |
| miR-575 | A_25_P00010810 | 0.553932584 | miR-568 | A_25_P00010933 | 0.583146067 |
| miR-575 | A_25_P00010808 | 0.55 | miR-568 | A_25_P00010934 | 0.574719101 |
| miR-575 | A_25_P00010809 | 0.566292135 | miR-549 | A_25_P00010366 | 0.565168539 |
| miR-376a* | A_25_P00010637 | 0.575842697 | miR-549 | A_25_P00010365 | 0.545505618 |
| miR-376a* | A_25_P00010638 | 0.562359551 | miR-549 | A_25_P00010367 | 0.538764045 |
| miR-376a* | A_25_P00010639 | 0.562921348 | miR-549 | A_25_P00010364 | 0.526966292 |
| miR-587 | A_25_P00011445 | 0.550561798 | miR-384 | A_25_P00010971 | 0.547752809 |
| miR-587 | A_25_P00011447 | 0.556741573 | miR-657 | A_25_P00011615 | 0.552247191 |
| miR-587 | A_25_P00011633 | 0.558426966 | miR-657 | A_25_P00011357 | 0.560674157 |
| miR-587 | A_25_P00011446 | 0.521910112 | miR-657 | A_25_P00011358 | 0.547752809 |
| miR-124a | A_25_P00011090 | 0.56741573 | miR-657 | A_25_P00011359 | 0.55 |
| miR-124a | A_25_P00011089 | 0.545505618 | miR-200a | A_25_P00010208 | 0.547752809 |
| miR-526a | A_25_P00010334 | 0.592696629 | miR-200a | A_25_P00010209 | 0.530337079 |
| miR-526a | A_25_P00010333 | 0.554494382 | miR-567 | A_25_P00010942 | 0.579213483 |
| miR-526a | A_25_P00010332 | 0.547752809 | miR-567 | A_25_P00010944 | 0.530337079 |
| miR-768-3p | A_25_P00011972 | 0.547191011 | miR-567 | A_25_P00010943 | 0.54494382 |
| miR-768-3p | A_25_P00011973 | 0.559550562 | miR-567 | A_25_P00010941 | 0.571348315 |
| miR-768-3p | A_25_P00011438 | 0.588764045 | miR-626 | A_25_P00011235 | 0.571910112 |
| miR-768-3p | A_25_P00011437 | 0.548876404 | miR-626 | A_25_P00010095 | 0.535393258 |
| miR-181a | A_25_P00010285 | 0.579775281 | miR-626 | A_25_P00011234 | 0.562921348 |
| miR-181a | A_25_P00010286 | 0.544382022 | miR-626 | A_25_P00010096 | 0.553932584 |
| miR-578 | A_25_P00011251 | 0.555617978 | miR-635 | A_25_P00011415 | 0.546629213 |
| miR-578 | A_25_P00011250 | 0.526404494 | miR-635 | A_25_P00011967 | 0.554494382 |
| miR-578 | A_25_P00011249 | 0.557865169 | miR-635 | A_25_P00011966 | 0.562921348 |
| miR-578 | A_25_P00011586 | 0.553370787 | miR-635 | A_25_P00011414 | 0.558426966 |
| miR-597 | A_25_P00011262 | 0.57247191 | miR-25 | A_25_P00010990 | 0.575280899 |
| miR-597 | A_25_P00011259 | 0.549438202 | miR-25 | A_25_P00010989 | 0.576404494 |
| miR-597 | A_25_P00011260 | 0.543258427 | miR-199a | A_25_P00010700 | 0.501123596 |
| miR-597 | A_25_P00011261 | 0.550561798 | miR-199a | A_25_P00010701 | 0.553370787 |
| miR-410 | A_25_P00010198 | 0.537078652 | miR-135a | A_25_P00010981 | 0.596067416 |
| miR-410 | A_25_P00010199 | 0.558426966 | miR-339 | A_25_P00011066 | 0.592696629 |
| miR-649 | A_25_P00011373 | 0.547752809 | miR-339 | A_25_P00011067 | 0.534831461 |
| miR-649 | A_25_P00011372 | 0.558988764 | miR-637 | A_25_P00011271 | 0.530898876 |
| miR-649 | A_25_P00011375 | 0.56011236 | miR-637 | A_25_P00011949 | 0.538764045 |
| miR-649 | A_25_P00011374 | 0.565730337 | miR-637 | A_25_P00011950 | 0.545505618 |
| miR-152 | A_25_P00010549 | 0.549438202 | miR-637 | A_25_P00011948 | 0.549438202 |
| miR-152 | A_25_P00010550 | 0.556179775 | miR-99a | A_25_P00010471 | 0.529775281 |
| miR-595 | A_25_P00010346 | 0.562921348 | miR-99a | A_25_P00010472 | 0.555617978 |
| miR-595 | A_25_P00010347 | 0.553932584 | miR-99a | A_25_P00010473 | 0.538202247 |
| miR-595 | A_25_P00010348 | 0.541573034 | miR-429 | A_25_P00010273 | 0.587078652 |
| miR-33 | A_25_P00010466 | 0.538764045 | miR-429 | A_25_P00010272 | 0.515730337 |
| miR-140 | A_25_P00010606 | 0.604494382 | miR-758 | A_25_P00011429 | 0.592696629 |
| miR-140 | A_25_P00010607 | 0.543820225 | miR-758 | A_25_P00011430 | 0.545505618 |
| miR-526b | A_25_P00010059 | 0.547752809 | miR-758 | A_25_P00011968 | 0.536516854 |
| miR-526b | A_25_P00010058 | 0.547191011 | miR-758 | A_25_P00011969 | 0.537078652 |
| miR-526b | A_25_P00010060 | 0.566853933 | miR-623 | A_25_P00010229 | 0.541011236 |
| miR-487a | A_25_P00010091 | 0.565168539 | miR-623 | A_25_P00010226 | 0.571348315 |
| miR-487a | A_25_P00010092 | 0.552808989 | miR-623 | A_25_P00010227 | 0.563483146 |
| miR-487a | A_25_P00010090 | 0.553370787 | miR-623 | A_25_P00010228 | 0.529213483 |
| miR-580 | A_25_P00011353 | 0.534269663 | miR-208 | A_25_P00010137 | 0.547752809 |
| miR-580 | A_25_P00011614 | 0.553932584 | miR-208 | A_25_P00010138 | 0.546067416 |
| miR-580 | A_25_P00011354 | 0.594382022 | miR-596 | A_25_P00011351 | 0.562921348 |
| miR-580 | A_25_P00011355 | 0.55 | miR-596 | A_25_P00011350 | 0.551685393 |
| miR-600 | A_25_P00011376 | 0.523033708 | miR-596 | A_25_P00011613 | 0.525842697 |
| miR-600 | A_25_P00011619 | 0.551123596 | miR-596 | A_25_P00011352 | 0.545505618 |
| miR-600 | A_25_P00011377 | 0.564044944 | miR-493-3p | A_25_P00010672 | 0.536516854 |
| miR-600 | A_25_P00011379 | 0.544382022 | miR-493-3p | A_25_P00010673 | 0.548314607 |
| miR-454-5p | A_25_P00011396 | 0.550561798 | miR-18b | A_25_P00010951 | 0.530898876 |
| miR-454-5p | A_25_P00010765 | 0.579213483 | miR-18b | A_25_P00010952 | 0.556741573 |
| miR-454-5p | A_25_P00011397 | 0.563483146 | miR-647 | A_25_P00011371 | 0.561235955 |
| miR-454-5p | A_25_P00010766 | 0.54494382 | miR-647 | A_25_P00011368 | 0.556741573 |
| miR-425-5p | A_25_P00010977 | 0.599438202 | miR-647 | A_25_P00011370 | 0.563483146 |
| miR-425-5p | A_25_P00010978 | 0.574157303 | miR-647 | A_25_P00011369 | 0.532022472 |
| miR-487b | A_25_P00010113 | 0.578651685 | miR-196b | A_25_P00010523 | 0.512921348 |
| miR-487b | A_25_P00010114 | 0.544382022 | miR-196b | A_25_P00010524 | 0.563483146 |
| miR-658 | A_25_P00011265 | 0.524157303 | miR-34c | A_25_P00010755 | 0.567977528 |
| miR-658 | A_25_P00011946 | 0.529213483 | miR-34c | A_25_P00010756 | 0.559550562 |
| miR-658 | A_25_P00011947 | 0.529775281 | miR-151 | A_25_P00010578 | 0.578651685 |
| miR-658 | A_25_P00011945 | 0.556741573 | miR-151 | A_25_P00010577 | 0.574157303 |
| miR-548b | A_25_P00010122 | 0.574157303 | miR-524* | A_25_P00010717 | 0.562359551 |
| miR-548b | A_25_P00010124 | 0.569662921 | miR-524* | A_25_P00010715 | 0.557865169 |
| miR-548b | A_25_P00010123 | 0.536516854 | miR-524* | A_25_P00010716 | 0.551123596 |
| miR-548b | A_25_P00010121 | 0.553932584 | miR-653 | A_25_P00011450 | 0.55 |
| miR-194 | A_25_P00011007 | 0.544382022 | miR-653 | A_25_P00010938 | 0.53988764 |
| miR-194 | A_25_P00011006 | 0.552247191 | miR-653 | A_25_P00011452 | 0.520786517 |
| miR-598 | A_25_P00011058 | 0.528089888 | miR-653 | A_25_P00011451 | 0.530337079 |
| miR-598 | A_25_P00011059 | 0.515730337 | miR-551a | A_25_P00011474 | 0.533707865 |
| miR-598 | A_25_P00011057 | 0.549438202 | miR-551a | A_25_P00011638 | 0.543820225 |
| miR-126* | A_25_P00010600 | 0.562359551 | miR-551a | A_25_P00011473 | 0.561797753 |
| miR-142-3p | A_25_P00011016 | 0.588764045 | miR-551a | A_25_P00011471 | 0.543258427 |
| hsa-let-7d | A_25_P00010282 | 0.529213483 | miR-518a | A_25_P00011008 | 0.543820225 |
| hsa-let-7d | A_25_P00010281 | 0.542696629 | miR-518a | A_25_P00011009 | 0.55505618 |
| miR-198 | A_25_P00010576 | 0.562921348 | miR-361 | A_25_P00010894 | 0.583146067 |
| miR-198 | A_25_P00010575 | 0.574719101 | miR-361 | A_25_P00010895 | 0.560674157 |
| miR-769-3p | A_25_P00011230 | 0.55 | miR-542-5p | A_25_P00010647 | 0.545505618 |
| miR-769-3p | A_25_P00011231 | 0.55 | miR-542-5p | A_25_P00010646 | 0.561797753 |
| miR-769-3p | A_25_P00011233 | 0.575280899 | miR-338 | A_25_P00010974 | 0.575280899 |
| miR-769-3p | A_25_P00011232 | 0.557865169 | miR-338 | A_25_P00010973 | 0.557303371 |
| miR-301 | A_25_P00010839 | 0.559550562 | miR-126 | A_25_P00010768 | 0.562359551 |
| miR-301 | A_25_P00010840 | 0.553370787 | miR-126 | A_25_P00010767 | 0.563483146 |
| miR-375 | A_25_P00010987 | 0.588764045 | miR-522 | A_25_P00010050 | 0.546629213 |
| miR-375 | A_25_P00010986 | 0.561235955 | miR-522 | A_25_P00010049 | 0.52752809 |
| miR-506 | A_25_P00011036 | 0.585393258 | miR-145 | A_25_P00010109 | 0.558988764 |
| miR-506 | A_25_P00011038 | 0.537640449 | miR-145 | A_25_P00010110 | 0.558426966 |
| miR-506 | A_25_P00011037 | 0.552247191 | miR-128a | A_25_P00010003 | 0.56741573 |
| miR-324-5p | A_25_P00010154 | 0.536516854 | miR-128a | A_25_P00010004 | 0.569662921 |
| miR-324-5p | A_25_P00010153 | 0.547191011 | miR-579 | A_25_P00011321 | 0.510674157 |
| miR-187 | A_25_P00010602 | 0.533707865 | miR-579 | A_25_P00011958 | 0.569662921 |
| miR-187 | A_25_P00010603 | 0.546629213 | miR-579 | A_25_P00011323 | 0.585393258 |
| miR-187 | A_25_P00010601 | 0.557303371 | miR-579 | A_25_P00011322 | 0.563483146 |
| miR-421 | A_25_P00010794 | 0.571910112 | miR-17-3p | A_25_P00010141 | 0.611235955 |
| miR-421 | A_25_P00010795 | 0.561797753 | miR-17-3p | A_25_P00010142 | 0.551685393 |
| miR-499 | A_25_P00011108 | 0.540449438 | miR-128b | A_25_P00010692 | 0.565730337 |
| miR-499 | A_25_P00011107 | 0.535955056 | miR-128b | A_25_P00010691 | 0.542134831 |
| hsa-let-7i | A_25_P00010185 | 0.526404494 | miR-31 | A_25_P00010510 | 0.520786517 |
| hsa-let-7i | A_25_P00010184 | 0.562921348 | miR-31 | A_25_P00010509 | 0.584269663 |
| miR-92 | A_25_P00010512 | 0.562921348 | miR-615 | A_25_P00010374 | 0.531460674 |
| miR-92 | A_25_P00010513 | 0.551123596 | miR-615 | A_25_P00010375 | 0.550561798 |
| miR-92 | A_25_P00010511 | 0.532022472 | miR-615 | A_25_P00010377 | 0.556179775 |
| miR-491 | A_25_P00011027 | 0.543258427 | miR-615 | A_25_P00010376 | 0.542134831 |
| miR-491 | A_25_P00011026 | 0.544382022 | miR-519a | A_25_P00010890 | 0.561797753 |
| miR-498 | A_25_P00010687 | 0.558988764 | miR-519a | A_25_P00010889 | 0.564606742 |
| miR-498 | A_25_P00010688 | 0.549438202 | miR-542-3p | A_25_P00011126 | 0.553932584 |
| miR-513 | A_25_P00010413 | 0.595505618 | miR-542-3p | A_25_P00011127 | 0.557303371 |
| miR-513 | A_25_P00010414 | 0.430337079 | miR-542-3p | A_25_P00011125 | 0.565168539 |
| miR-642 | A_25_P00011338 | 0.552808989 | miR-30a-3p | A_25_P00010827 | 0.585393258 |
| miR-642 | A_25_P00011335 | 0.542696629 | miR-30a-3p | A_25_P00010828 | 0.540449438 |
| miR-642 | A_25_P00011337 | 0.593258427 | miR-520a | A_25_P00010201 | 0.553370787 |
| miR-642 | A_25_P00011336 | 0.529213483 | miR-520a | A_25_P00010200 | 0.516853933 |
| miR-489 | A_25_P00010829 | 0.531460674 | miR-204 | A_25_P00010399 | 0.548876404 |
| miR-489 | A_25_P00010830 | 0.6 | miR-204 | A_25_P00010400 | 0.56011236 |
| miR-422a | A_25_P00010856 | 0.543258427 | hsa-let-7e | A_25_P00010280 | 0.567977528 |
| miR-422a | A_25_P00010857 | 0.537640449 | hsa-let-7e | A_25_P00010279 | 0.544382022 |
| miR-422a | A_25_P00010855 | 0.554494382 | miR-17-5p | A_25_P00010277 | 0.580898876 |
| miR-548c | A_25_P00010742 | 0.555617978 | miR-17-5p | A_25_P00010278 | 0.569662921 |
| miR-548c | A_25_P00010743 | 0.535393258 | miR-613 | A_25_P00011422 | 0.575842697 |
| miR-548c | A_25_P00010741 | 0.56011236 | miR-613 | A_25_P00011627 | 0.547191011 |
| miR-548c | A_25_P00010740 | 0.558988764 | miR-613 | A_25_P00011421 | 0.569101124 |
| miR-299-3p | A_25_P00010103 | 0.538202247 | miR-613 | A_25_P00011628 | 0.528651685 |
| miR-299-3p | A_25_P00010104 | 0.565730337 | miR-550 | A_25_P00010448 | 0.537640449 |
| miR-101 | A_25_P00010678 | 0.576966292 | miR-550 | A_25_P00010447 | 0.584269663 |
| miR-101 | A_25_P00010679 | 0.596067416 | miR-550 | A_25_P00010449 | 0.533707865 |
| miR-99b | A_25_P00010596 | 0.551123596 | miR-183 | A_25_P00010515 | 0.53258427 |
| miR-99b | A_25_P00010597 | 0.525280899 | miR-183 | A_25_P00010516 | 0.547752809 |
| miR-181c | A_25_P00010287 | 0.520786517 | miR-223 | A_25_P00010880 | 0.513483146 |
| miR-181c | A_25_P00010288 | 0.536516854 | miR-223 | A_25_P00010879 | 0.554494382 |
| miR-660 | A_25_P00010461 | 0.552808989 | miR-449b | A_25_P00010776 | 0.552808989 |
| miR-660 | A_25_P00010460 | 0.564606742 | miR-449b | A_25_P00010774 | 0.551685393 |
| miR-660 | A_25_P00010459 | 0.558988764 | miR-449b | A_25_P00010775 | 0.537640449 |
| miR-27a | A_25_P00010797 | 0.550561798 | miR-412 | A_25_P00010267 | 0.557303371 |
| miR-27a | A_25_P00010796 | 0.611797753 | miR-412 | A_25_P00010269 | 0.534831461 |
| miR-93 | A_25_P00010611 | 0.546629213 | miR-412 | A_25_P00010266 | 0.55 |
| miR-93 | A_25_P00010610 | 0.552247191 | miR-412 | A_25_P00010268 | 0.537078652 |
| miR-185 | A_25_P00010703 | 0.562359551 | miR-520b | A_25_P00010565 | 0.573033708 |
| miR-185 | A_25_P00010702 | 0.548876404 | miR-520b | A_25_P00010563 | 0.535955056 |
| miR-26a | A_25_P00010779 | 0.575842697 | miR-520b | A_25_P00010564 | 0.548314607 |
| miR-26a | A_25_P00010780 | 0.596629213 | miR-328 | A_25_P00010263 | 0.553932584 |
| miR-373 | A_25_P00010157 | 0.581460674 | miR-328 | A_25_P00010262 | 0.562921348 |
| miR-373 | A_25_P00010156 | 0.551123596 | miR-218 | A_25_P00010211 | 0.561235955 |
| miR-373 | A_25_P00010155 | 0.544382022 | miR-218 | A_25_P00010210 | 0.542696629 |
| miR-518f* | A_25_P00010359 | 0.518539326 | hsa-let-7f | A_25_P00010089 | 0.542134831 |
| miR-518f* | A_25_P00010358 | 0.573033708 | hsa-let-7f | A_25_P00010088 | 0.542696629 |
| miR-28 | A_25_P00010630 | 0.570786517 | miR-565 | A_25_P00010005 | 0.55 |
| miR-28 | A_25_P00010631 | 0.529213483 | miR-565 | A_25_P00010007 | 0.557865169 |
| miR-130a | A_25_P00010440 | 0.526966292 | miR-565 | A_25_P00010006 | 0.562921348 |
| miR-130a | A_25_P00010439 | 0.552247191 | miR-423 | A_25_P00010651 | 0.549438202 |
| miR-191* | A_25_P00010877 | 0.55505618 | miR-423 | A_25_P00010650 | 0.566853933 |
| miR-191* | A_25_P00010875 | 0.591573034 | miR-129 | A_25_P00010754 | 0.54494382 |
| miR-191* | A_25_P00010878 | 0.534269663 | miR-129 | A_25_P00010753 | 0.542696629 |
| miR-191* | A_25_P00010876 | 0.589325843 | miR-802 | A_25_P00011244 | 0.553370787 |
| miR-516-5p | A_25_P00010036 | 0.51741573 | miR-802 | A_25_P00011247 | 0.580898876 |
| miR-516-5p | A_25_P00010035 | 0.525842697 | miR-802 | A_25_P00011245 | 0.531460674 |
| miR-197 | A_25_P00010836 | 0.571910112 | miR-802 | A_25_P00011246 | 0.550561798 |
| miR-197 | A_25_P00010835 | 0.506179775 | miR-515-5p | A_25_P00010499 | 0.569662921 |
| miR-563 | A_25_P00011629 | 0.550561798 | miR-515-5p | A_25_P00010500 | 0.546067416 |
| miR-563 | A_25_P00011425 | 0.525842697 | miR-646 | A_25_P00011963 | 0.535955056 |
| miR-563 | A_25_P00011426 | 0.534831461 | miR-646 | A_25_P00010781 | 0.548876404 |
| miR-563 | A_25_P00011427 | 0.55 | miR-646 | A_25_P00011962 | 0.543820225 |
| miR-524 | A_25_P00010291 | 0.558426966 | miR-646 | A_25_P00011400 | 0.565730337 |
| miR-524 | A_25_P00010292 | 0.552808989 | miR-627 | A_25_P00011030 | 0.561235955 |
| miR-590 | A_25_P00010655 | 0.562921348 | miR-627 | A_25_P00011031 | 0.574719101 |
| miR-590 | A_25_P00010654 | 0.553932584 | miR-627 | A_25_P00011033 | 0.541011236 |
| miR-590 | A_25_P00010656 | 0.561235955 | miR-627 | A_25_P00011032 | 0.534831461 |
| miR-551b | A_25_P00010648 | 0.557865169 | miR-650 | A_25_P00010918 | 0.562359551 |
| miR-551b | A_25_P00010649 | 0.579775281 | miR-650 | A_25_P00010917 | 0.529213483 |
| miR-455 | A_25_P00010594 | 0.563483146 | miR-650 | A_25_P00010919 | 0.54494382 |
| miR-455 | A_25_P00010595 | 0.563483146 | miR-564 | A_25_P00010786 | 0.545505618 |
| miR-452 | A_25_P00010924 | 0.568539326 | miR-564 | A_25_P00010785 | 0.558426966 |
| miR-452 | A_25_P00010925 | 0.538202247 | miR-564 | A_25_P00010787 | 0.556179775 |
| miR-10a | A_25_P00010469 | 0.536516854 | miR-323 | A_25_P00010379 | 0.52752809 |
| miR-10a | A_25_P00010470 | 0.618539326 | miR-323 | A_25_P00010378 | 0.528089888 |
| miR-370 | A_25_P00010542 | 0.490449438 | miR-525* | A_25_P00010812 | 0.541011236 |
| miR-370 | A_25_P00010541 | 0.517977528 | miR-525* | A_25_P00010811 | 0.53258427 |
| miR-370 | A_25_P00010543 | 0.579775281 | miR-483 | A_25_P00010335 | 0.553370787 |
| miR-370 | A_25_P00010544 | 0.498876404 | miR-483 | A_25_P00010337 | 0.560674157 |
| miR-190 | A_25_P00010239 | 0.551685393 | miR-483 | A_25_P00010336 | 0.553370787 |
| miR-409-5p | A_25_P00011073 | 0.542134831 | miR-382 | A_25_P00010585 | 0.585955056 |
| miR-409-5p | A_25_P00011072 | 0.540449438 | miR-382 | A_25_P00010586 | 0.55505618 |
| miR-409-5p | A_25_P00011074 | 0.552247191 | miR-382 | A_25_P00010587 | 0.537640449 |
| miR-371 | A_25_P00010161 | 0.55 | miR-618 | A_25_P00011591 | 0.579213483 |
| miR-371 | A_25_P00010160 | 0.557303371 | miR-618 | A_25_P00011282 | 0.532022472 |
| miR-146a | A_25_P00010079 | 0.564044944 | miR-618 | A_25_P00011280 | 0.53988764 |
| miR-146a | A_25_P00010078 | 0.562359551 | miR-618 | A_25_P00011281 | 0.54494382 |
| miR-15b | A_25_P00011102 | 0.55505618 | miR-29a | A_25_P00010082 | 0.565168539 |
| miR-15b | A_25_P00011101 | 0.546067416 | miR-29a | A_25_P00010083 | 0.526966292 |
| miR-92b | A_25_P00010729 | 0.55 | miR-144 | A_25_P00010626 | 0.557865169 |
| miR-92b | A_25_P00010730 | 0.536516854 | miR-144 | A_25_P00010627 | 0.543258427 |
| miR-92b | A_25_P00010728 | 0.566292135 | miR-552 | A_25_P00011121 | 0.562921348 |
| miR-566 | A_25_P00010946 | 0.556741573 | miR-552 | A_25_P00011479 | 0.550561798 |
| miR-566 | A_25_P00011458 | 0.569662921 | miR-552 | A_25_P00011480 | 0.543820225 |
| miR-566 | A_25_P00010945 | 0.550561798 | miR-552 | A_25_P00011481 | 0.548876404 |
| miR-566 | A_25_P00011457 | 0.552247191 | miR-432 | A_25_P00010530 | 0.530337079 |
| miR-136 | A_25_P00011017 | 0.552247191 | miR-432 | A_25_P00010532 | 0.578651685 |
| miR-136 | A_25_P00011018 | 0.554494382 | miR-432 | A_25_P00010531 | 0.542696629 |
| miR-206 | A_25_P00010528 | 0.564044944 | miR-505 | A_25_P00010726 | 0.565168539 |
| miR-206 | A_25_P00010527 | 0.534269663 | miR-505 | A_25_P00010727 | 0.566292135 |
| miR-206 | A_25_P00010529 | 0.580898876 | miR-505 | A_25_P00010725 | 0.548314607 |
| miR-583 | A_25_P00010893 | 0.534269663 | miR-505 | A_25_P00010724 | 0.526966292 |
| miR-583 | A_25_P00010892 | 0.562921348 | miR-19a | A_25_P00010998 | 0.574719101 |
| miR-583 | A_25_P00010891 | 0.569662921 | miR-19a | A_25_P00010997 | 0.580337079 |
| miR-553 | A_25_P00011220 | 0.52247191 | miR-630 | A_25_P00010249 | 0.542134831 |
| miR-553 | A_25_P00011221 | 0.52752809 | miR-630 | A_25_P00010247 | 0.518539326 |
| miR-553 | A_25_P00011219 | 0.546629213 | miR-630 | A_25_P00010248 | 0.561797753 |
| miR-553 | A_25_P00011222 | 0.507865169 | miR-630 | A_25_P00010246 | 0.548876404 |
| miR-591 | A_25_P00011252 | 0.537078652 | miR-33b | A_25_P00011078 | 0.538764045 |
| miR-591 | A_25_P00011254 | 0.553932584 | miR-33b | A_25_P00011076 | 0.561235955 |
| miR-591 | A_25_P00011587 | 0.598314607 | miR-33b | A_25_P00011075 | 0.549438202 |
| miR-591 | A_25_P00011253 | 0.56741573 | miR-33b | A_25_P00011077 | 0.575842697 |
| miR-383 | A_25_P00010385 | 0.539325843 | miR-142-5p | A_25_P00010896 | 0.56011236 |
| miR-383 | A_25_P00010383 | 0.524719101 | miR-572 | A_25_P00011096 | 0.585955056 |
| miR-383 | A_25_P00010384 | 0.571910112 | miR-572 | A_25_P00011095 | 0.575842697 |
| miR-514 | A_25_P00010624 | 0.542134831 | miR-572 | A_25_P00011097 | 0.563483146 |
| miR-514 | A_25_P00010625 | 0.526966292 | miR-517c | A_25_P00010168 | 0.53988764 |
| miR-514 | A_25_P00010622 | 0.557865169 | miR-517c | A_25_P00010167 | 0.580898876 |
| miR-514 | A_25_P00010623 | 0.528651685 | miR-302b | A_25_P00010618 | 0.546629213 |
| miR-148b | A_25_P00010133 | 0.568539326 | miR-302b | A_25_P00010621 | 0.542134831 |
| miR-148b | A_25_P00010134 | 0.539325843 | miR-302b | A_25_P00010620 | 0.563483146 |
| miR-554 | A_25_P00011289 | 0.537640449 | miR-302b | A_25_P00010619 | 0.547191011 |
| miR-554 | A_25_P00011287 | 0.538764045 | miR-520g | A_25_P00010170 | 0.558426966 |
| miR-554 | A_25_P00011288 | 0.562359551 | miR-520g | A_25_P00010171 | 0.551123596 |
| miR-554 | A_25_P00011593 | 0.545505618 | miR-520g | A_25_P00010169 | 0.538764045 |
| miR-518b | A_25_P00010244 | 0.548876404 | miR-585 | A_25_P00010962 | 0.59494382 |
| miR-518b | A_25_P00010245 | 0.553932584 | miR-585 | A_25_P00010960 | 0.523595506 |
| miR-106a | A_25_P00010801 | 0.565730337 | miR-585 | A_25_P00010961 | 0.529775281 |
| miR-106a | A_25_P00010802 | 0.525842697 | miR-448 | A_25_P00010443 | 0.533707865 |
| miR-611 | A_25_P00010901 | 0.537078652 | miR-448 | A_25_P00010441 | 0.534831461 |
| miR-611 | A_25_P00010902 | 0.565730337 | miR-448 | A_25_P00010442 | 0.550561798 |
| miR-611 | A_25_P00010903 | 0.531460674 | miR-448 | A_25_P00010444 | 0.541011236 |
| miR-518e | A_25_P00010177 | 0.556179775 | miR-485-5p | A_25_P00010707 | 0.565168539 |
| miR-518e | A_25_P00010176 | 0.538764045 | miR-485-5p | A_25_P00010706 | 0.519101124 |
| miR-770-5p | A_25_P00011978 | 0.562921348 | miR-485-5p | A_25_P00010708 | 0.567977528 |
| miR-770-5p | A_25_P00011477 | 0.528089888 | miR-511 | A_25_P00011061 | 0.55 |
| miR-770-5p | A_25_P00011476 | 0.535955056 | miR-511 | A_25_P00011062 | 0.556741573 |
| miR-770-5p | A_25_P00011979 | 0.56011236 | miR-511 | A_25_P00011063 | 0.537640449 |
| miR-510 | A_25_P00011105 | 0.565168539 | miR-511 | A_25_P00011060 | 0.533707865 |
| miR-510 | A_25_P00011106 | 0.538764045 | miR-488 | A_25_P00011119 | 0.546629213 |
| miR-593 | A_25_P00011406 | 0.551685393 | miR-488 | A_25_P00011120 | 0.543258427 |
| miR-593 | A_25_P00011408 | 0.541011236 | miR-621 | A_25_P00011599 | 0.544382022 |
| miR-593 | A_25_P00011409 | 0.541573034 | miR-621 | A_25_P00011311 | 0.55505618 |
| miR-593 | A_25_P00011625 | 0.539325843 | miR-621 | A_25_P00011312 | 0.567977528 |
| miR-656 | A_25_P00010380 | 0.531460674 | miR-621 | A_25_P00011309 | 0.56741573 |
| miR-520e | A_25_P00010561 | 0.570786517 | miR-302d | A_25_P00010163 | 0.534269663 |
| miR-520e | A_25_P00010560 | 0.529775281 | miR-302d | A_25_P00010164 | 0.528651685 |
| miR-520e | A_25_P00010562 | 0.554494382 | miR-302d | A_25_P00010162 | 0.56741573 |
| miR-34b | A_25_P00010465 | 0.587078652 | miR-559 | A_25_P00011643 | 0.55 |
| miR-34b | A_25_P00010464 | 0.603932584 | miR-559 | A_25_P00011642 | 0.547752809 |
| miR-130b | A_25_P00010438 | 0.579213483 | miR-559 | A_25_P00011131 | 0.556179775 |
| miR-130b | A_25_P00010437 | 0.539325843 | miR-559 | A_25_P00011132 | 0.566853933 |
| miR-29c | A_25_P00010238 | 0.558988764 | miR-29b | A_25_P00010054 | 0.580898876 |
| miR-100 | A_25_P00010475 | 0.493820225 | miR-29b | A_25_P00010053 | 0.534269663 |
| miR-100 | A_25_P00010474 | 0.562921348 | miR-520c | A_25_P00010080 | 0.569101124 |
| miR-143 | A_25_P00011065 | 0.544382022 | miR-520c | A_25_P00010081 | 0.536516854 |
| miR-143 | A_25_P00011064 | 0.565730337 | miR-107 | A_25_P00011069 | 0.535393258 |
| miR-520f | A_25_P00011217 | 0.574719101 | miR-107 | A_25_P00011068 | 0.563483146 |
| miR-520f | A_25_P00011942 | 0.552247191 | miR-368 | A_25_P00010013 | 0.548876404 |
| miR-409-3p | A_25_P00010408 | 0.556179775 | miR-368 | A_25_P00010012 | 0.537078652 |
| miR-409-3p | A_25_P00010407 | 0.535393258 | miR-527 | A_25_P00010327 | 0.55505618 |
| miR-181b | A_25_P00010552 | 0.556179775 | miR-527 | A_25_P00010325 | 0.557303371 |
| miR-181b | A_25_P00010551 | 0.571910112 | miR-527 | A_25_P00010326 | 0.546629213 |
| miR-560 | A_25_P00010863 | 0.549438202 | miR-133b | A_25_P00010964 | 0.554494382 |
| miR-560 | A_25_P00010865 | 0.551685393 | miR-133b | A_25_P00010963 | 0.547752809 |
| miR-560 | A_25_P00010864 | 0.524719101 | miR-504 | A_25_P00010418 | 0.552247191 |
| miR-603 | A_25_P00011386 | 0.517977528 | miR-504 | A_25_P00010417 | 0.544382022 |
| miR-603 | A_25_P00011384 | 0.529213483 | miR-18a | A_25_P00010970 | 0.551123596 |
| miR-603 | A_25_P00011385 | 0.537078652 | miR-18a | A_25_P00010969 | 0.58988764 |
| miR-603 | A_25_P00011387 | 0.548314607 | miR-330 | A_25_P00011047 | 0.569662921 |
| miR-523 | A_25_P00010567 | 0.548314607 | miR-330 | A_25_P00011046 | 0.570224719 |
| miR-523 | A_25_P00010568 | 0.552808989 | miR-330 | A_25_P00011048 | 0.552247191 |
