# Supplementary material for: Comprehensive miRNA Expression Analysis in Peripheral Blood Can Diagnose Liver Disease
Source: PLoS One. 2012 Oct 31;7(10):e48366. doi: 10.1371/journal.pone.0048366 (PMC3485241; doi:10.1371/journal.pone.0048366)
Supplement: Supplemental Information — (DOCX) [file pone.0048366.s021.docx]

Supporting Information

1 “*in silico”*patients resampling

Insufficient number of patients in a sample can cause the following two types of problems:

1*. Sampling bias:* Although the number of patients in a sample is sufficiently large, the sample may have been collected in a way that invariably excludes some members of the intended population.

2. *Sampling error:* Although randomly selected and therefore lack bias, the characteristics of the sample may deviate from that of the total population being represented by the sample.

An example of sampling bias is age. If patients are older than the normal control, any observed differences between patients and healthy controls may be due to age and not the disease being researched. There is often a minimum number of patients necessary for most research. If for example an investigation is being done of the yearly progress of a disease over ten years, at least twenty patients would be needed since both a male and female patient are necessary for each of the ten years. If a study has a single patient for each year, e.g. a male patient for the first year, a female patient for the second year, the sample would not be sufficient to produce reliable results, therefore the outcome would inevitably be biased.

In order to eliminate possible sample bias or error it is necessary throughout the duration of a study to enroll a sample that is sufficiently large. If this is not possible, a method known as “*in silico*” resampling can be used to weaken both sample bias and error that could possibly result from using a small sample.

Suppose we have *N*patients with *M*types of clinical information
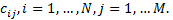

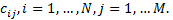
then the “*in silico*” resampling for the *l*th patient can be done by picking one
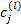

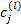
 for each *j* from
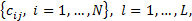

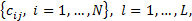
 where *L* is the number of resampled patients.

In our study, in order to infer the miRNA expression attributed to the *l*th resampled patient, we used the MCMC regress function in the MCMC pack^[[1]](#footnote-1)^ to conduct a Markov Chain Monte Carlo (MCMC) regression.

Before the inference, we modeled the relationship between miRNA expression and clinical information. We assumed that the *i*th patient's *k*th miRNA expression,
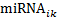

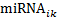
, is related to clinical information as follows:


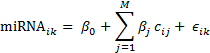


The MCMC regression generates
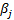

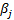
and
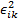

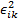
 that obey the following distributions:


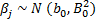


and


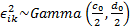

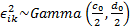
,

Where *N* and *Gamma* are the Gaussian and the Gamma distribution respectively. Using MCMC, we generated the parameter set,
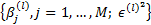

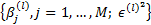
, to represent the miRNA expression of the *l*th resampled patient,
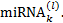

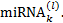
Then
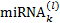

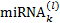
was generated using the following formula:


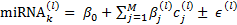

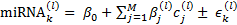

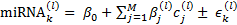
,

where
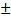

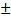
 takes
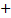

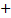
 or
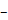

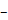
 with equal probability.

1.1 “*in silico”* resampling for disease discriminant studies

In discriminating among CHC, NASH, CHB and normal controls, we also used “*in silico*” resampling. The clinical information considered for the modeling were age, gender and body mass index (BMI). The *i*th patient's miRNA expression was modeled as:


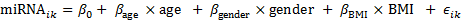


where age and BMI are actual values and gender is 1 (male) or 0 (female). For each miRNA selected to discriminate between two patient groups, MCMC was applied. We then used one hundred resampled patients to generate miRNA expression in each group. This means, the clinical information of the virtual patients in each group was the same. Then linear discriminant analysis (LDA) with principal component analysis (PCA) was applied to discriminate and distribute two hundred patients into two groups.

The results of the “*in silico*” resampling are shown in Supp. Figures S3-S9. Fig. S3 shows the discriminant analysis for CHC, CHB, NASH and NL.

In Fig. S3B we can see that the accuracy is 95.25 %, which is comparable with Fig. 3A. As mentioned above, “*in silico”* resampling generates miRNA expression for a clinical cohort with common age, gender and BMI; this suggests that these four diseased/healthy groups would be well distinguishable even if this analysis were to be repeated using a larger patient sample with common clinical traits.

Reflecting the smallest number of samples of CHB, distribution of “*in silico*” resampling of CHB is scattered, but is still determined from CHC, NASH and NL. Using an optimal number of principal components produced the fairly good results that are shown in Fig. S3B.

Similar observations can be seen in other figures such as S4-S9. As shown in figures S4-S6, discriminating between CHB and CHC, CHC and NASH, and CHC and NL was done with 100% accuracy. These are comparative with or even better than the corresponding accuracies in Fig. 3 and Fig. 2B, i.e., 98.5 %, 97.2 %, and 97.6 %. This means that the relatively large number of CHC patients enabled the “*in silico*" resampling to accurately reproduce the miRNA expression for the corresponding clinical trait. In figures S7-S9, discriminating between CHB and NL, NASH and NL, and CHB and NASH, was done with 90.5 %, 95 %, and 95.5% accuracy respectively. These are also comparable to, or even better than the corresponding accuracies in Figs. 3 of 70.8 %, 77.8 %, and 81.8 %. Since “*in silico*" does not have any mechanism to make discriminating between two diseases easier, these results suggest that accuracy will increase if more patients are considered.

1.2 “*in silico*” resampling for qPCR validation of microarray results

In the validation of the microarray results by qPCR, we once again used “*in silico*” resampling. In addition to clinical information employed in the previous section, we also used inflammation and fibrosis stage. Meaning, miRNA expression by qPCR or microarray was modeled as:


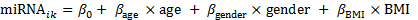


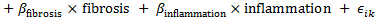


where both inflammation grades and fibrosis stages are 0, 1, 2, and 3. Based on this model, both qPCR and 16 microarray probes values were generated from one thousand resampled patients. The coincidence between qPCR and microarray expression averaged over 16 probes was checked. The performance by “*in silico*” resampling is shown in Suppl. Figure S11.

It is rather clear that “*in silico*” resampling successfully reproduced the miRNA expression of each clinical trait. This suggests that even if more patients were included in the sample the results here would not change.

1.3 The accuracy of “*in silico”* resampling

Although it is apparently successful, there may still be doubts about the accuracy of “*in silico”*resampling. Thus, we confirmed the accuracy of “*in silico”* using known samples. Raw data of 99 CHC liver miRNA expression patterns were downloaded from GEO ID: GSE16922. Ten samples were selected randomly ten times. Each time a selection was made, we attempted to reproduce the miRNA expression for the remaining 89 CHCusing“*in silico”* resampling.

We then checked if it were possible to discriminate between “*in silico”*virtual samples and true samples using linear discriminant analysis. Even without applying cross validation, we obtained an average accuracy across all miRNAs of 0.55. This means that resampled miRNA expressions cannot be statistically distinguished from true miRNA expressions. Thus we can conclude that “*in silico”* can statistically reproduce a sample population from a segment of an available sample. In table S6, we have listed the accuracy for each probe.

2 Canonical correlation coefficients between miRNA expression and clinical information

In this study, individual correlation coefficient between miRNA and clinical information (ALT, albumin, and HCVRNA) was not large enough, although associated *P*-values were very small. Therefore, we will demonstrate how to increase this correlation coefficient when the canonical correlation coefficient is considered instead of the correlation coefficient.

2.1 Demonstrating the usefulness of canonical correlation coefficients

Suppose that the *i*th patient has the *j*th clinical information
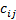

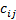
,and the *k*th miRNA expression
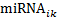

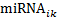
, then the correlation coefficient between the *j*th clinical information and the *k*th miRNA expression for all patients would be:


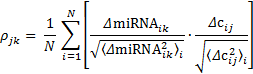


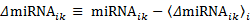


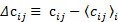


where


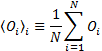


In this study,
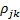

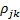
s were at most 0.3 (not shown here), which is not sufficiently large. However, the introduction of the canonical correlation coefficient can compensate for this. The canonical correlation coefficient denotes the correlation between the set of
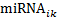

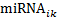
s and a specific
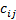

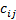
,


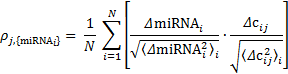


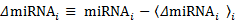


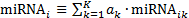

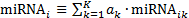
,

where
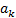

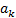
s are taken to maximize
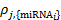

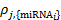
and *K* is the number miRNAs under consideration.

In order to demonstrate how
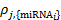

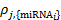
 can increase even if
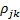

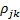
s is not large, we performed the following numerical simulation.
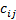

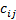
 was replaced with an artificial series with values
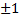

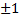
 and
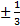

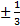
.
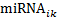

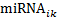
was replaced with
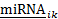

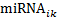

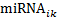
s, the summation of
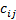

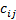
and uniform random number
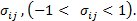

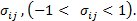


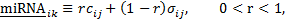


If
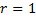

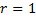
 then
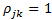

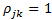
, since
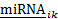

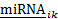
is identical to
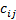

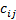
. On the other hand,
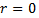

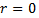
results in
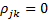

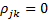
, because
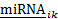

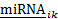
is the pure random number. Thus, *r*controls the correlation between
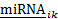

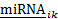
and
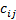

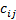
. The number of patients *N* was 64, the same as the number of CHC patients.
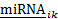

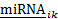
s was generated one thousand times and the resulting
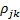

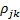
s was averaged. *R* was assigned a value of 0.15, because the average value of
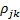

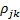
was as large as 0.20 when r=0.15. A value of=0.20 is most suitable since it reflects the typical value of the correlation coefficient between miRNA expression and clinical information in this study.

Next, by generating ten , we computed the canonical correlation coefficient between and . The canonical correlation was computation one thousand times and the resultings was averaged. The averaged value was 0.57, which is three times larger than . This demonstrates that the canonical correlation coefficient can be substantially greater than the individual correlation coefficient between and and can depict correlation hidden behind any added noise.

2.2 Canonical correlation coefficients between miRNAs and clinical information

We computed the canonical correlation coefficients for ALT, albumin, and HCVRNA and the *K*miRNAs. *K*miRNAs had the largest absolute value of the correlation coefficients, , and *K* was taken from 1 to 12.

Associated *P*-values were also computed. The results are shown in Fig. 6 and Table 4. In each table in Table 4, the second column shows the miRNAs in descending order based upon the absolute value of the correlation coefficients of the clinical information, i.e., ALT, albumin, and HCVRNA. The third column shows the canonical correlation coefficient using the miRNAs included in the second column, and the fourth column are associated *P*-values. Although the canonical correlation coefficient monotonically increases as the number of miRNAs used increases, *P*-values do not always decrease since the increase in the canonical correlation coefficients should be greater than that resulting from any additional noise component. *P*-values do not decrease as the number of miRNAs increase. Thus, the optimal number of miRNAs is one that maximizes the value of the correlation coefficient with a *P*-value less than 0.05.

3.semi-supervised learning for the second cohort

As mentioned in our paper, we prepared an independent sample set to 　validate our methods.　For this purpose, we employed semi-supervised learning [2]. At first, two disease samples, e.g., CHB and CHC, were extracted from both the original and independent cohort. PCA was then applied after miRNAs other than those shown in the corresponding (“CHC/CHB”) section of Table 2 were discarded. Next, LDA was applied to the optimal number of PCs with only the labels (CHB or CHC) for the original sample. Finally, the classifications in the independent sample were inferred from the resulting discriminant function. The optimal number of PCs was determined so that the best results could be obtained.

2. Chapelle O, Scholkopf B, Zien A (2006) Semi-supervised learning. Cambridge, Mass.: MIT Press. x, 508 p. p.

1. http://mcmcpack.wustl.edu/, all parameters other than or are default values.andare set to be 1.0. [↑](#footnote-ref-1)
